# Adaptive Immunity to Dengue Virus: Slippery Slope or Solid Ground for Rational Vaccine Design?

**DOI:** 10.3390/pathogens9060470

**Published:** 2020-06-15

**Authors:** Lucas Wilken, Guus F. Rimmelzwaan

**Affiliations:** Research Centre for Emerging Infections and Zoonoses (RIZ), University of Veterinary Medicine Hannover, Foundation (TiHo), Bünteweg 17, 30559 Hannover, Germany; lucas.wilken@tiho-hannover.de

**Keywords:** dengue virus, vaccine, antibodies, T cells, correlates of protection, immunopathogenesis

## Abstract

The four serotypes of dengue virus are the most widespread causes of arboviral disease, currently placing half of the human population at risk of infection. Pre-existing immunity to one dengue virus serotype can predispose to severe disease following secondary infection with a different serotype. The phenomenon of immune enhancement has complicated vaccine development and likely explains the poor long-term safety profile of a recently licenced dengue vaccine. Therefore, alternative vaccine strategies should be considered. This review summarises studies dissecting the adaptive immune responses to dengue virus infection and (experimental) vaccination. In particular, we discuss the roles of (*i*) neutralising antibodies, (*ii*) antibodies to non-structural protein 1, and (*iii*) T cells in protection and pathogenesis. We also address how these findings could translate into next-generation vaccine approaches that mitigate the risk of enhanced dengue disease. Finally, we argue that the development of a safe and efficacious dengue vaccine is an attainable goal.

## 1. Background

### 1.1. Dengue Epidemiology, Clinical Disease and Immunopathogenesis

Dengue virus (DENV) is the most prevalent mosquito-borne viral pathogen, currently placing half of the human population at risk of infection [1], with an estimated annual global incidence of 390 million cases [2]. DENV is a member of the genus *Flavivirus* in the family *Flaviviridae*, alongside other important human pathogens such as Zika virus (ZIKV), yellow fever virus (YFV), West Nile virus (WNV), and Japanese encephalitis virus (JEV). There are four antigenically distinct serotypes, DENV1–4, that differ by 30–35% at the amino acid level, with each being further divided into multiple genotypes [3]. DENV1–4 co-circulate, mainly, in the tropical and subtropical regions of the world, following the distribution of their vectors *Aedes aegypti* and *Aedes albopictus* [4]. The geographic range of these mosquitoes is, however, dramatically expanding, driven by the globalisation of trade and travel, rapid unplanned urbanisation, and climate change [5]. For example, *Ae. albopictus* has established itself in Southern Europe where, following importation of DENV-infected travellers, several cases of autochthonous transmission have been reported [6].

Estimates suggest that a quarter of all DENV infections become clinically apparent [2]. The most common form of disease, dengue fever (DF), is a mild flu-like syndrome characterised by the rapid onset of fever in combination with severe headache, arthralgia, myalgia, retro-orbital pain, and a rash [7]. Patients with dengue haemorrhagic fever (DHF), the more severe form of disease, show all the symptoms of DF in combination with thrombocytopenia, coagulopathy and, most importantly, plasma leakage—to which the risk of hypotension and circulatory collapse (dengue shock syndrome (DSS)) is associated [8]. Severe dengue accounts for two million cases each year, of which 12,500 have fatal outcomes [9].

Primary DENV infection usually results in long-term protection against the infecting (homologous) serotype [10,11]—although there have been cases of symptomatic reinfections [12,13]—but only short-term cross-protection against other (heterologous) serotypes [10,14,15]. When short-term cross-protection wanes, patients with secondary DENV infections are at higher risk of severe disease [16,17,18,19], revealing a role of pre-existing immunity in dengue pathogenesis. Two opposing concepts of immunopathogenesis came into existence: the leading hypothesis, termed antibody-dependent enhancement (ADE), posits that cross-reactive antibodies from the previous DENV infection bind, but cannot neutralise, the heterologous virus and facilitate its uptake into Fc gamma receptor (FcγR)–bearing cells, thereby increasing viral load and ultimately disease severity [20,21]. Supporting evidence comes from cell culture [22,23,24], animal models [24,25,26,27], and cohort studies [28,29,30,31]. The other hypothesis is based on the phenomenon of ‘original antigenic sin’, whereby previous exposure to a cross-reactive antigen shapes the subsequent adaptive immune response to a related antigen [32]. It suggests that cross-reactive T cells generated during primary DENV infection are selectively expanded during secondary DENV infection, but that these demonstrate only low avidity for the heterologous infecting serotype, leading to delayed viral clearance and aberrant cytokine responses that exacerbate disease severity [33,34]. More recent studies, however, strongly support a protective rather than a pathogenic role for cross-reactive T cells [35].

### 1.2. Biology of DENV

**Figure 1:**
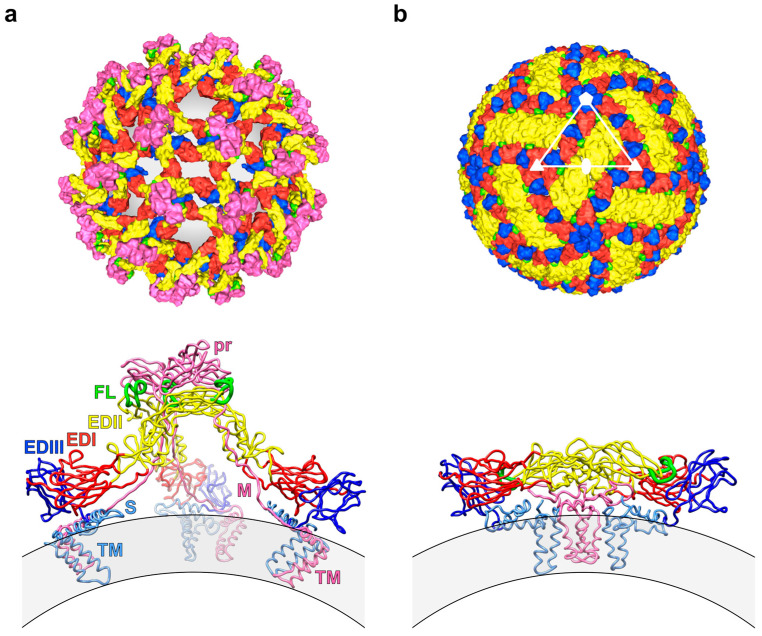
Structural architecture of immature and mature dengue virions. (**a**) Upper panel: Cryo-electron microscopy (cryo-EM) structure of the immature dengue virus 1 (DENV1) particle carrying 60 trimeric precursor membrane (prM)–E spikes (PDB 4B03) in surface representation. Lower panel: Side view of a single trimeric prM–E spike in ribbon form. (**b**) Upper panel: Cryo-EM structure of the mature DENV1 particle with 90 E protein dimers (PDB 4CCT) in surface representation. An icosahedral asymmetric unit is indicated by a white triangle and the icosahedral vertices are marked by white symbols: two-fold, ellipse; three-fold, triangle; and five-fold, pentagon. Lower panel: Side view of a single E protein dimer and the underlying M proteins in ribbon form. Colours correspond between the upper and lower panels. The host-derived lipid bilayer is depicted in grey. Molecular graphics were prepared with the Protein Imager [74] (upper panels) or UCSF Chimera [75] (lower panels). E protein domain I (EDI); E protein domain II (EDII); E protein domain III (EDIII); fusion loop (FL); stem region (S); transmembrane anchor (TM); precursor peptide (pr); membrane protein (M).

DENV is a small enveloped virus with a positive-sense single-stranded RNA genome encoding a single polyprotein that is processed co- and post-translationally by viral and host proteases into three structural proteins—capsid (C) protein, precursor membrane (prM) or membrane (M) protein, and envelope (E) protein—as well as seven non-structural proteins (termed NS1, NS2A, NS2B, NS3, NS4A, NS4B, and NS5). The C protein associates with the viral genome, forming a nucleocapsid that is surrounded by a host-derived lipid bilayer, into which the prM and E proteins are embedded in immature virions, or the M and E proteins in mature virions (Figure 1).

Cryo-electron microscopy (cryo-EM) structures of the mature dengue virion revealed a smooth surface constituted by 180 copies each of M and E proteins, anchored to the underlying lipid bilayer through their transmembrane helices (Figure 1b). The surface proteins are arranged in a pseudo-icosahedral fashion, with each of the 60 asymmetric units consisting of three pairs of M and E proteins. The three individual E proteins in an asymmetric unit exist in distinct chemical environments defined by their proximity to the two-, three-, or five-fold vertices [36,37,38,39]. The E protein monomer consists of three structural domains (E protein domains I, II and III (EDI, EDII and EDIII)—containing two N-linked glycosylation sites (Asn67 and Asn153) [40]—, and two of these protomers associate into a head-to-tail homodimer [41,42,43]. Three E protein dimers lie in parallel to each other, building a raft, and 30 of these rafts are arranged in a characteristic ‘herringbone’ pattern [36,37,38,39].

Viral attachment to target cells—primarily of the myeloid lineage—is thought to occur through EDIII [44,45,46,47,48] and the Asn67-linked glycan in EDII [49,50]. Several cell surface molecules—including heparan sulphate [44], dendritic cell–specific ICAM3-grabbing non-integrin (DC-SIGN) [51,52], mannose receptor [50] and phosphatidylserine receptors [53]—have been implicated in DENV binding, but a single receptor that is necessary for entry has not yet been defined. After attachment, DENV enters cells by clathrin-dependent, receptor-mediated endocytosis [54]. The acidification of the endosome causes the E protein dimers to dissociate and reorganise into trimers, exposing the hydrophobic fusion loop (FL) of EDII at their tips [55,56]. The FL then inserts into the endosomal membrane, resulting in the fusion of viral and endosomal membranes [55,56] and the subsequent delivery of the nucleocapsid into the cytoplasm [57].

Following uncoating, translation and genome replication, the prM and E proteins are embedded into the endoplasmic reticulum (ER) membrane and enclose the newly formed nucleocapsid as it buds into the lumen of the ER, forming an immature viral particle [58,59]. The immature dengue virion has a rough surface with 60 spikes, each formed by three prM–E heterodimers in which the precursor (pr) peptide of the prM protein caps the FL located at the distal end of each E protein monomer [38,60,61] (Figure 1a). During export, the low-pH environment of the trans-Golgi network (TGN) triggers the rearrangement of prM–E heterodimers into a flattened conformation, exposing a cleavage site at the pr-M junction that is recognised by the host protease furin [62,63]. The cleaved pr peptide remains associated with the E protein under acidic conditions, preventing premature fusion [64,65]. Upon release into the extracellular milieu, the neutral pH induces the dissociation of the pr peptide from the viral particle, completing the virion maturation process [65].

The cleavage of prM protein is often inefficient, with infected cells secreting a heterogeneous mixture of fully immature, partially mature and fully mature virions [66,67,68,69,70]. The efficiency of this cleavage event appears to differ between cell types, as primary human cells such as dendritic cells yield viral particles with much lower levels of prM protein than those derived from insect or mammalian cell lines (e.g., Vero cells) [70,71]. Fully immature virions are inherently non-infectious, as the presence of prM protein prevents the structural rearrangement of E proteins required for membrane fusion [63,64,67,72]. Partially mature virions, however, contain two discrete regions of immature and mature structure, with the latter being devoid of prM protein and therefore potentially capable of initiating infection [68,73].

### 1.3. Dengue Vaccines

Developing a safe and efficacious vaccine against dengue remains a challenging task. As discussed above, immunity induced by exposure to one serotype does not confer long-term protection against secondary infection with one of the other three serotypes and is potentially capable of enhancing this infection. It is generally believed that a vaccine will need to induce durable, protective responses against all four serotypes; thus, the use of tetravalent vaccines is considered necessary. The field has been dominated by three tetravalent live-attenuated dengue vaccines, which are discussed below.

**Figure 2:**
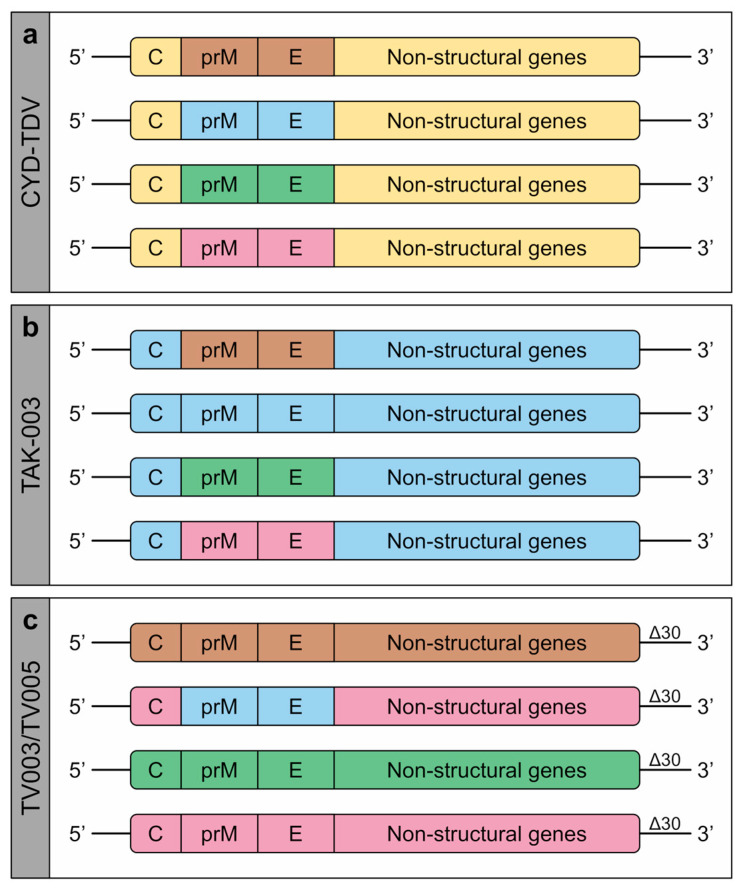
Tetravalent live-attenuated dengue vaccines. (**a**) CYD-TDV employs the YFV-17D vaccine strain (shown in yellow) as a genetic backbone for the expression of the prM and E genes of DENV1 (brown), DENV2 (blue), DENV3 (green), and DENV4 (pink). (**b**) TAK-003 consists of an attenuated DENV2 strain and three chimeric viruses expressing the prM and E genes of DENV1, 3 and 4 in the context of the DENV2 genetic backbone. (**c**) TV003/TV005 is composed of three full-length viruses containing all wild-type structural and non-structural genes, and one chimeric virus, in which the prM and E genes of DENV4 are substituted by those of DENV2. These viruses are attenuated by a common 30-nt deletion (Δ30) in the 3′ UTR of the viral genome.

CYD-TDV (formally known as Dengvaxia; Sanofi Pasteur)—currently licenced in 20 endemic countries—is a chimeric vaccine using the YFV-17D vaccine strain as a genetic backbone for the expression of the prM and E genes of each DENV serotype [76] (Figure 2a). In phase I clinical trials, seroconversion to all four serotypes was observed in 100% of participants after three doses [77,78]. However, in phase IIb and III clinical trials, CYD-TDV showed poor efficacy against DENV2 (34.7%) and only moderate efficacy against DENV1, 3 and 4 (54.5%, 65.2%, and 72.4%, respectively) [79,80,81]. Long-term safety analyses found an increased relative risk of dengue, leading to hospitalisation among participants under the age of 9 years (1.58) and for those aged 2–5 as high as 7.45 [82]. Their young age suggests a lower likelihood of previous exposure to DENV, and it is thought that CYD-TDV mimics a primary infection in naïve individuals, sensitising them to more severe disease upon subsequent infection [83,84]. CYD-TDV has therefore been restricted for use in individuals aged 9 years and older. More recently, a case–cohort study compiling data from these clinical trials reported a higher risk of hospitalisation and severe dengue among individuals that were seronegative at baseline, irrespective of age [85]. Following a nationwide paediatric vaccination campaign, CYD-TDV was suspended in the Philippines in 2017, due to a high incidence of severe dengue among vaccinees [86]. The Strategic Advisory Group of Experts (SAGE) on Immunisation has since recommended a pre-vaccination screening to determine the serostatus of recipients, where feasible, or otherwise only vaccinating populations with documented seroprevalence rates above 80% in the age group of 9 years and older [87].

TAK-003 (formally known as DENVax; Takeda) is a vaccine candidate consisting of the attenuated DENV2 strain PDK-53 [88] and three chimeric viruses expressing the prM and E genes of DENV1, 3 and 4 in the context of the DENV2 PDK-53 genetic backbone [89] (Figure 2b). Its immunogenicity has been evaluated in phase I and II clinical trials, where 62% of study participants seroconverted to all four serotypes and 96% to at least three serotypes after two doses, with the highest seroconversion rate to DENV2 (>95%) and the lowest to DENV4 (87.5%) [90,91]. A phase III clinical trial assessing vaccine efficacy is currently ongoing. Primary efficacy data have recently been reported, showing 80.2% overall efficacy, 95.4% efficacy against dengue leading to hospitalisation, and 74.9% efficacy in seronegative participants. However, efficacy varied according to serotype and was found to be highest against DENV2 (97.7%), lower against DENV1 (73.7%) and DENV3 (62.6%), and inconclusive against DENV4 [92]. Similar results were also observed after six months of additional follow-up [93]. Long-term efficacy and safety data for TAK-003 are expected in 2021.

TV003/TV005 (formally known as LATV Δ30; National Institutes of Health (NIH)) is a tetravalent vaccine candidate attenuated by a common 30-nucleotide deletion in the 3′ untranslated region (UTR) of the viral genome [94]. Three components (rDEN1Δ30, rDEN3Δ30 and rDEN4Δ30) are full-length viruses containing all wild-type structural and non-structural genes, and one component (rDEN2/4Δ30) is a chimeric virus, in which the prM and E genes of DENV4 are substituted by those of DENV2 [95] (Figure 2c). TV003 and TV005 are two different formulations, with the latter containing an increased dose of the DENV2 component. In phase I clinical trials, a single dose of TV003 induced seroconversion to all four serotypes in 74% and to at least three serotypes in 92% of individuals, but imparted sterilising immunity against a second vaccine dose [96,97,98]. Despite a low seroconversion rate to DENV2 (76%), vaccinees were completely protected against a controlled DENV2 challenge at six months post-immunisation [99]. A phase II clinical trial was recently completed, and a phase III clinical trial is currently ongoing, with results expected in 2025.

### 1.4. Scope of This Review

Thus far, tetravalent live-attenuated dengue vaccines have produced disappointing—if not alarming—results in phase III clinical trials and post-licensure, regarding efficacy/effectiveness and long-term safety. This emphasises the need to reconsider current vaccine strategies.

Although CYD-TDV vaccination elicited high titres of neutralising antibodies in seronegative individuals, as measured in vitro, these did not correlate with protection in vivo [100]. It seems that the quality, rather than the magnitude, of the neutralising antibody response against DENV is the crucial factor [101], which is the focus of Section 2 of this review.

Moreover, the poor efficacy of CYD-TDV might also be attributable to its lack of expressing DENV NS1, which was shown to elicit protective antibodies in mice [102] and has since emerged as an alternative vaccine candidate. We therefore also discuss novel insights into the functional properties of NS1-specific antibodies in Section 3 of this review.

Finally, CYD-TDV contains the C and non-structural proteins of YFV but not those of DENV1–4, and in TAK-003, which was less efficacious against DENV1 and DENV3, these are derived only from DENV2. There is accumulating evidence that these viral proteins are important targets of a protective DENV-specific T-cell response [103]. Therefore, Section 4 of this review is dedicated to recent findings about the T-cell responses induced by natural infection and vaccination.

## 2. Neutralising Antibodies against DENV

### 2.1. The Neutralising Antibody Response to DENV Infection

During primary infection, the activation of DENV-specific naïve B cells gives rise to both antibody-secreting long-lived plasma cells (LLPCs), which reside primarily in the bone marrow, and memory B cells (MBCs), which circulate through the blood and secondary lymphoid organs. Extensive analyses of monoclonal antibodies (mAbs) and polyclonal sera of individuals with history of a primary DENV infection revealed that the majority of antibodies is cross-reactive and weakly neutralising, and that only a minor proportion of antibodies is responsible for durable, strong serotype-specific neutralisation [104,105,106,107,108,109,110,111,112,113,114,115]. Transient immunity to heterologous serotypes observed after primary infection is thought to depend on the concentration of cross-reactive antibodies in serum. At high concentrations, cross-reactive antibodies induce the formation of large viral aggregates able to cross-link inhibitory FcγRIIB, thereby blocking infection and avoiding ADE [116]; however, as their levels are declining over time, there is an increased risk of ADE due to sub-neutralising antibody concentrations [30]. Upon secondary infection with a heterologous DENV serotype, cross-reactive MBCs generated during primary infection preferentially expand and dominate over serotype-specific responses [70,111,112,115,117,118,119,120,121,122,123]. The resulting cross-reactive antibodies were shown to have higher binding avidities and neutralising potencies than those found in primary DENV infection, being able to neutralise not only the current and previous infecting serotype but also serotypes to which individuals have not yet been exposed (‘non-exposed’ serotypes) [112,113,117,118,124,125,126]. It is thought that these strongly neutralising cross-reactive antibodies contribute to protection against the non-exposed serotypes, as suggested by the low incidence of symptomatic tertiary and quaternary infections [127,128,129].

### 2.2. Impact of the Structural Heterogeneity and Dynamics of DENV on Antibody-Mediated Neutralisation

**Figure 3:**
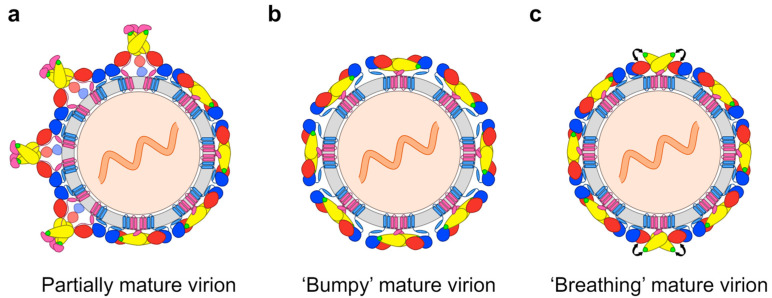
The changing antigenic landscape of dengue virions. Schematic representations of the diverse viral morphologies arising from inefficient prM protein cleavage (only one type of prM protein–containing particle is shown for simplicity) (**a**), exposure to temperatures of 34 °C and above—unique to DENV2—(**b**), or the sampling of multiple E protein conformations at equilibrium (**c**), each of which individually influences antibody-mediated neutralisation of DENV, by virtue of modulating epitope accessibility. The curved double-headed black arrows in **c** indicate viral breathing motions, which in this example transiently expose the otherwise buried FL. Colour coding for the viral surface proteins is identical to that in Figure 1. Lipid bilayer and nucleocapsid core are shown in grey and orange, respectively.

Antibody-mediated neutralisation of flaviviruses is a ‘multiple-hit’ phenomenon occurring when antibodies bind to virions at a stoichiometry that exceeds a certain threshold, with the most potent antibodies neutralising when approximately 30 binding sites have been occupied [130]. Antibodies engaging virions at a stoichiometry that falls below this threshold cannot neutralise them and instead facilitate their uptake into FcγR-bearing cells [130]. DENV has been shown to adopt diverse morphologies (see Figure 3)—with different antigenic properties—that significantly influence the stoichiometry of antibody binding and, thus, neutralising activities.

Firstly, as described above, DENV is released from infected cells as a mixed population of structurally distinct viral particles with varying levels of prM protein, resulting from an incomplete maturation process [66,67,68,69,70] (Figure 3a). Studies comparing the maturity of dengue virions produced in mosquito versus primary human cells suggest that, following a mosquito bite, the first round of infection is mediated by viruses with high levels of prM protein, whereas the viruses released from infected human cells will contain lower levels of prM protein [70,71]. This is further supported by the apparent absence of prM protein on human plasma–derived virions [131]. The structural heterogeneity of DENV affects the potency of certain neutralising antibodies because their epitopes are differentially accessible in trimeric prM–E spikes and E protein dimers [68,132,133].

Secondly, while the surfaces of mature virions are smooth at the temperature of their mosquito hosts, certain strains of DENV2 irreversibly acquire an expanded ‘bumpy’ conformation upon exposure to human physiological temperature [39,134,135,136] (Figure 3b). In the bumpy particles, the elevated temperature has loosened the interactions within and between E protein dimers, and caused the E protein layer to move outward [134,135], thereby exposing previously hidden (‘cryptic’) antibody binding sites [134,137,138]. In contrast, bumpy surfaces are not observed for DENV1 and DENV4, presumably due to stronger E-M protein interactions in these viruses [39].

Thirdly, the conformational flexibility of E proteins on the virion surface causes them to be in continuous, dynamic motion—a phenomenon termed ‘viral breathing’—making cryptic epitopes transiently accessible for antibody binding [138,139] (Figure 3c). In contrast to the temperature-induced conformational changes, however, the structural rearrangements arising from viral breathing are reversible [133]. The rate of viral breathing, moreover, seems to vary between strains, as reflected by time-dependent differences in neutralisation sensitivity [140,141].

In summary, the varying degrees of maturity and the structural ensembles sampled by DENV must be considered when defining the targets of antibodies and the functional consequences of their binding.

### 2.3. Antibodies That Target the Fusion Loop Epitope in the E Protein

Several screening studies observed immunodominance of the EDI/II region in DENV-immune donors and found that it was mainly targeted by cross-reactive antibodies displaying weak neutralising activity [104,105,106,110,121,142,143,144,145]. The majority of these antibodies have been mapped to a region comprising the FL in EDII (amino acids 98 to 110), termed the fusion loop epitope (FLE) [71,104,105,110,123,142,144,146]. Trp101 has been identified as a key residue for the binding of various mAbs to the FLE, with many also being sensitive to substitution of the neighbouring residues Gly106, Leu107, and Phe108 [104,105,109,123,125,147,148,149,150]. High conservation at these amino acid positions enables FLE-specific mAbs to cross-react with all DENV serotypes as well as other flaviviruses [68,104,105,142,146,148,150,151,152]. Moreover, the FLE shows enhanced immunogenicity in secondary heterologous infections [123,144,152], which is likely to be a result of original antigenic sin.

Studies using the prototypic mouse mAbs 4G2 [153] and E53 [148] or panels of human mAbs have provided insight into the binding and functional properties of antibodies that target the FLE. FLE-specific mAbs were shown to preferentially bind to immature and partially mature virions [68,132], with cryo-EM reconstructions revealing that the FLE is partially solvent-exposed in trimeric prM–E spikes [68] (Figure 1a). Flaviviruses grown in cells with limited furin activity, and thus containing high levels of prM protein, are effectively neutralised by FLE-specific mAbs [71,132]. The virus–antibody interactions are more complex in the context of the mature virion, in which the FLE is largely inaccessible, lying buried between the two subunits of the E protein dimer [68] (Figure 1b). Antibodies to the FLE therefore depend on dimer dissociation for their binding. Prolonged incubation enabled FLE-specific mAbs to bind to fully mature virions, indicating that the FLE had become accessible through dynamic motion of the E proteins [133,138] (see Figure 3c). In line with these observations, escape from mAb 4G2 was found to be mediated by substitutions at E protein residues distant from the FLE [154], which presumably govern epitope exposure by modulating the rate of viral breathing. Given the cryptic nature of the FLE in mature virions, enough antibody binding sites may thus not be continuously available to reach the stoichiometric threshold required for neutralisation. Accordingly, FLE-specific mAbs were unable to fully neutralise viruses produced in primary human cells or cells overexpressing furin, even at high concentrations [71,132,155], but potently enhanced their infectious properties [71,132].

Another group of cross-reactive mAbs, isolated from patients with secondary infection, target conserved residues near the FL and in the bc loop of EDII [109,125]. These were of higher avidity than mAbs derived from individuals with primary infection—presumably due to affinity maturation [125]—and were shown to compete for binding against the poorly neutralising FLE-specific mAbs [109,125]. Moreover, these mAbs exhibited very potent neutralising activity when tested against standard preparations of each DENV serotype [109,125]. Despite being able to block the infectivity of cell culture–derived virions, the two mAbs studied, 1C19 and 1M7, could not effectively neutralise highly infectious, fully mature virions present in the plasma of viraemic patients [131]. This suggests a similar sensitivity to the virion maturation state as previously observed for FLE-specific mAbs and supports the view that in vitro neutralising activities, as they are currently measured, are not necessarily representative of the in vivo situation.

Antibodies binding within the FL or at proximal sites seem not to be the ideal response to be elicited with dengue vaccines and should therefore be avoided. All tetravalent live-attenuated dengue vaccines express wild-type E proteins and are therefore potentially capable of inducing this type of antibody response. Masking these epitopes by introducing substitutions into the FL is not an option for live-attenuated vaccines because such mutations can be lethal to the virus [156]. It is possible, however, to employ this strategy for recombinant subunit vaccines, which are not dependent on viral replication. For example, the use of prM-E–based DNA vaccines harbouring substitutions within the FL (G106R and L107D) that significantly reduced the induction of antibodies associated with immune enhancement relative to wild-type vaccines [157,158]. Similarly, a set of four mutations (T76R, Q77E, W101R, and L107R) in or near the FL was shown to reduce the induction of DENV-enhancing antibodies by a ZIKV prM-E mRNA vaccine [159].

### 2.4. PrM Protein–Specific Antibodies

Analyses of immune sera and memory B cell repertoires have identified the prM protein as another dominant target of the human antibody response to DENV infection [70,104,105,106,108,117,145]. Most prM protein–specific antibodies were found to recognise a single major antigenic site on the pr peptide [70,117,146,160,161,162,163], whereas others appeared to engage a complex quaternary epitope with shared sites on the prM–E heterodimer [105,163,164,165,166]. prM protein–specific antibodies generally display a high degree of cross-reactivity across the four serotypes but only limited neutralising activity, even at high concentrations [70,105,106,108,161,163,164,167,168]. Yet, one study demonstrated that some prM protein–specific mouse mAbs, though only weakly neutralising in vitro, could confer protection against lethal viral challenge in vivo and that this correlated with the ability to fix complement [169].

Fully mature virions are deficient in prM protein and therefore not susceptible to neutralisation by prM protein–specific antibodies, and neutralisation of partially mature particles is thought to require a threshold density of prM protein [70,170]. On the other hand, non-infectious fully immature virions as well as partially mature particles with below-threshold densities of prM protein are opsonised by prM protein–specific antibodies and taken up into FcγR-bearing cells, leading to increased viral replication [70,72,105,163,167,171,172]. The infectivity of fully immature virions appears to be restored in the endosome through furin-mediated cleavage of the prM protein [171] and subsequent low pH–induced displacement of the pr:antibody complex [173], finally exposing the FL for interaction with the endosomal membrane. The infection-enhancing properties of prM protein–specific antibodies have furthermore been demonstrated in a mouse model of severe dengue disease [26]. Several groups propose that inefficient prM protein cleavage, leading to the induction of poorly neutralising prM protein–specific antibodies, might be an immune evasion/enhancement strategy of DENV [70,170,174].

The majority of dengue vaccine candidates currently under preclinical investigation or in advanced clinical stages include expression of the prM protein and several of these are produced in Vero cells, in which prM protein cleavage is inefficient [67], thus yielding virus preparations containing particles with varying levels of unprocessed prM protein. Immunisation would therefore most certainly induce non-protective prM protein–specific antibody responses. This issue might, however, be overcome by generating dengue vaccines in furin-overexpressing cells with improved prM protein cleavage [175] or by introducing cleavage-enhancing substitutions into the prM protein of the vaccine strains [176]. Alternatively, one could exploit the minimal cross-reactivity of prM protein–specific antibodies between DENV and members of the JEV serocomplex for the production of improved chimeric vaccines [70,177]. In fact, replacement of the DENV pr peptide with its JEV counterpart or expression of DENV protein subunits in a JEV backbone proved to be an effective measure to reduce the enhancing activity of vaccine-induced antibodies while retaining full neutralising capacity [178,179,180].

### 2.5. Antibodies that Bind E Protein Domain III

#### 2.5.1. Insights from EDIII-Specific Mouse MAbs

**Figure 4:**
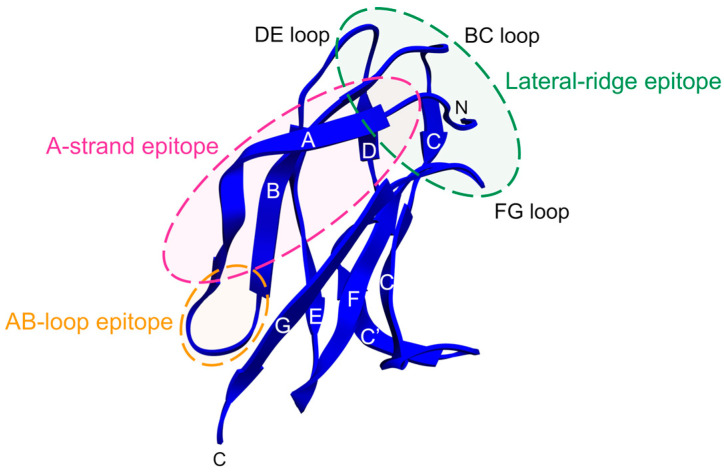
Major antigenic regions on EDIII. Ribbon diagram of EDIII, extracted from the cryo-EM structure of the mature DENV1 particle (PDB 4CCT), with the three main epitopes defined by mouse mAbs circled by dashed lines. Secondary structure assignments and labels according to previous models [182]. Molecular graphics were prepared with UCSF Chimera [75].

Most of our knowledge of the antibody response to EDIII has come from studies using mAbs isolated from DENV-infected or EDIII-immunised mice. Mouse mAbs specific for EDIII were shown to be more potent neutralisers of DENV than those recognising sites in EDI/II [45,140,181,182,183,184]. These antibodies generally neutralise by blocking viral attachment to the cell surface, in line with the proposed role of EDIII in receptor binding [45]. Moreover, neutralisation is mediated, to various extents, by both serotype-specific and cross-reactive antibodies recognising adjacent epitopes on EDIII, which are described below and illustrated in Figure 4.

Serotype-specific mouse mAbs to EDIII inhibit DENV infection most efficiently—often achieving 50% neutralisation in the sub-nanomolar range—and predominantly engage a sequence-unique epitope on the lateral ridge (BC, DE and FG loops) of EDIII [144,182,183,185,186,187,188]. Antibodies recognising the EDIII lateral ridge epitope generally exhibit a relatively low stoichiometric neutralisation threshold [130,132,185], presumably due to high accessibility of this site on mature virions. Moreover, exposure of the EDIII lateral ridge appears not to be affected by the presence of unprocessed prM protein, as antibodies to this epitope were found to neutralise viral particles regardless of their maturation state [132].

An epitope primarily containing residues of the A strand of EDIII, which is more conserved than the EDIII lateral ridge, is the target of numerous cross-reactive mouse mAbs with moderate, but broad, neutralising activity [144,182,184,186,189,190,191,192,193]. Well-characterised examples from this group are mAbs 1A1D-2 [181] and 4E11 [194]. Both antibodies bind and neutralise DENV1–3 [137,189], whereas 4E11 also weakly inhibits DENV4 infection by engaging additional conserved residues on the G strand [195]. The 1A1D-2 epitope is partially occluded in the smooth mature virion but becomes exposed through temperature-induced changes of the virion surface—note the larger solvent-accessible surface area of EDIII in Figure 3b—, and antibody binding eventually traps the E proteins in an intermediate conformation [137]. This is also thought to be the case for the 4E11 epitope [195,196]. Both antibodies interfere with viral attachment to the target cell surface [45,190], essentially because their binding disrupts the mature virion architecture [137,195]. It has been suggested that antibodies neutralising by this mechanism may have a lower occupancy requirement for neutralisation—thus reducing the risk of ADE—as compared to antibodies that neutralise solely by sterically blocking receptor engagement [137]. Variants of 4E11, engineered to neutralise DENV4 more strongly, have proven successful in both prophylactic and therapeutic settings in mice [197,198].

EDIII is also targeted by cross-reactive non-neutralising mouse mAbs that recognise an epitope in the highly conserved AB loop with limited exposure on the mature virion [182,199,200,201]. It is thought that the low accessibility of this epitope directly influences the stoichiometry of antibody binding, with the result that the threshold for neutralisation is often not reached [130,182]. Despite their inability to neutralise viral infectivity, these mAbs generally show no enhancing activity [199,201], possibly because their binding mode does not allow interaction with FcγRs on myeloid cells.

#### 2.5.2. Human Antibodies to EDIII

EDIII-specific human mAbs display similar characteristics as their mouse analogues in terms of potency and the antigenic sites targeted: (*i*) they neutralise DENV more strongly than EDI/II-reactive mAbs [105,110,142]; (*ii*) they bind serotype-specific and cross-reactive epitopes on the lateral ridge and the A strand of EDIII, respectively [105,106,152,202]. The EDIII-specific antibody response during primary infection is mostly directed to serotype-specific determinants, whereas a shift towards more conserved regions is observed during secondary infection [143,152,203]. However, EDIII-specific antibodies constitute only a minor proportion of the total antibody response to natural infection in humans [105,108,121,142,143]. Moreover, human immune sera depleted of EDIII-specific antibodies retained most of their neutralising activity [143,203,204] and recombinant viruses with mutations in neutralising epitopes of EDIII were still efficiently neutralised by untreated sera [202]. Together, these studies demonstrated that EDIII-specific antibodies contribute little to the neutralising potency of human polyclonal sera and that this is predominantly accounted for by other groups of antibodies (discussed later). This lack of response to EDIII during natural infection in humans, however, opens the door to subunit vaccines based on EDIII.

#### 2.5.3. EDIII as a Vaccine Candidate

Numerous groups have investigated the potential of EDIII-based vaccines in experimental animals (reviewed in [205]). Initially, EDIII was produced as a recombinant fusion protein in bacteria, but was found either not to be immunogenic in mice [206] or to depend on multiple immunisations for the induction of protective neutralising antibodies, which still waned over time [207]. To improve its immunogenicity, EDIII has since been expressed in the context of various immunological carriers, including meningococcal P64k protein [208], lipoproteins [209], plasmid vectors [210,211,212], viral vectors [213,214], and virus-like particles (VLPs) [215,216]. These vaccine candidates generally elicited serotype-specific, strongly neutralising antibody responses that were long-lasting. For some, protection against lethal viral challenge has been demonstrated in mice [210,217,218,219] or non-human primates [220,221,222]. Notably, EDIII-induced antibodies showed some infection-enhancing activity in vitro [219,223,224]—yet significantly less than DENV2 antisera and FLE-specific mAbs [219,224]—but this was not observed in vivo [219,225,226]. Furthermore, two groups have attempted to produce EDIII-based vaccines that induce broadly neutralising antibodies by using a consensus sequence approach [227] or by masking non-conserved epitopes [228]. However, antibody responses were non-protective following immunisation, thus, requiring further optimisation.

One potential drawback of immunisation with EDIII is that it not only elicits strongly neutralising antibodies to surface epitopes but also non-neutralising antibodies directed to antigenic sites that, though accessible on EDIII, are cryptic in the virion (e.g., the AB loop) [199,200], which might result in inefficient antibody responses to DENV. Some studies therefore suggest that EDIII would be better suited as a booster antigen in a heterologous prime–boost regimen with whole-virion vaccines, thereby focussing the antibody response to surface-exposed, critical neutralising sites on this domain [229,230]. Up to now, no EDIII-based vaccine candidate has advanced past the preclinical stage.

### 2.6. Antibodies that Target Quaternary Epitopes on the Virion

Though most DENV-neutralising antibodies have been mapped to the E protein ectodomain, it appears that a large fraction of DENV-neutralising antibodies in humans binds to higher-order E protein structures present only on intact virions [70,105,106,107,110,112,143]. To date, several strongly neutralising human mAbs recognising such complex quaternary epitopes have been isolated and characterised extensively.

#### 2.6.1. Human MAbs to Serotype-Specific Quaternary Epitopes

**Figure 5:**
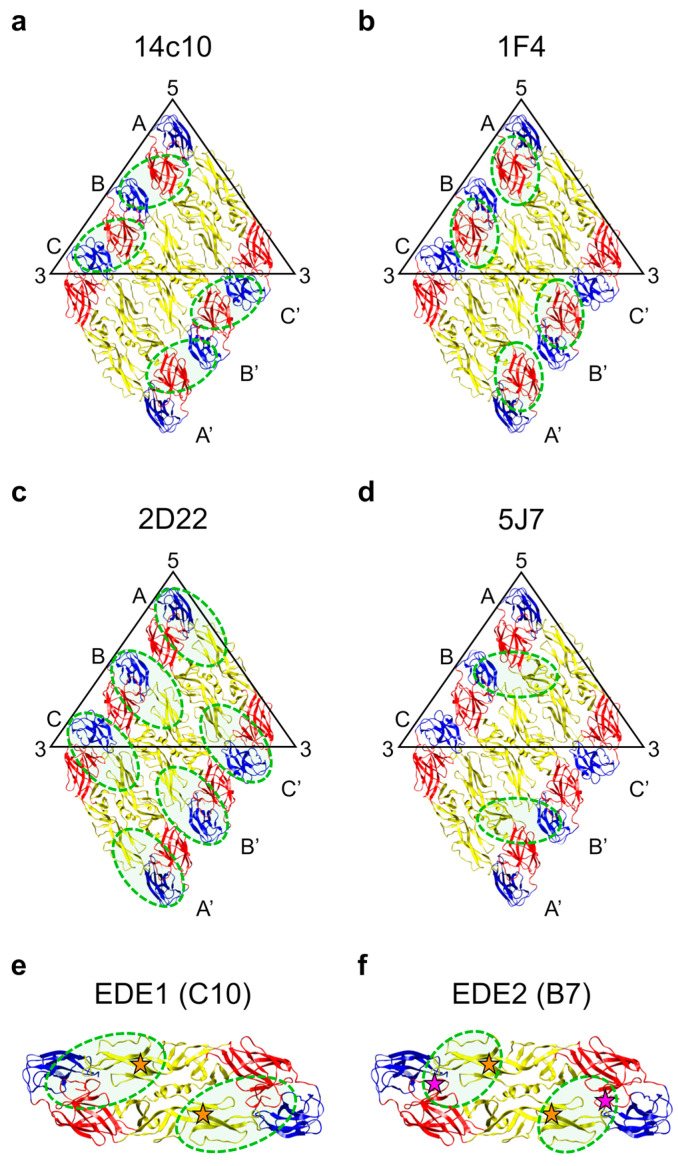
Quaternary epitopes recognised by strongly neutralising human mAbs. (**a**–**d**) Serotype-specific epitopes on the E protein raft bound by human mAbs 14c10 (DENV1) [231] (**a**), 1F4 (DENV1) [107,110,232] (**b**), 2D22 (DENV2) [107,108,233] (**c**), and 5J7 (DENV3) [107,108,235] (**d**). The epitopes are circled by green dashed lines. The black triangle represents an icosahedral asymmetric unit and the numbers indicate the vertices. The three E protein molecules in the asymmetric unit are labelled as A, B and C, respectively, and those in the neighbouring asymmetric unit, A’, B’ and C’, respectively. (**e**,**f**) Cross-reactive epitopes on the E protein dimer bound by E protein dimer epitope 1 (EDE1)-specific mAbs (here: mAb C10) (**e**) and EDE2-specific mAbs (here: mAb B7) (both [71,236]) (**f**). The epitopes are circled by green dashed lines. The glycosylation sites on Asn67 are marked as orange stars. Moreover, to highlight the sensitivity of EDE2-specific mAbs to glycosylation at Asn153, these sites are marked as pink stars only in **f**. Structures of the different E protein arrangements were extracted from the cryo-EM structure of the mature DENV1 particle (PDB 4CCT) and molecular graphics were prepared with UCSF Chimera [75] (**a**–**f**).

The first human mAb identified was 14c10, a potent neutraliser of DENV1 [231]. This antibody engages an epitope bridging two adjacent E protein dimers, with one half of its binding determinants located in EDIII, and the other half in EDI and the EDI–EDII hinge region of a neighbouring E protein (Figure 5a). 14c10 principally neutralises DENV1 by blocking viral attachment and was found to be highly protective in mice when administered prophylactically or therapeutically [231]. Its binding site partially overlaps with that of another DENV1-specific neutralising human mAb, 1F4 [107,110], at the EDI–EDII hinge, which indicates that this region may be important in eliciting serotype-specific antibody responses in humans [232]. Though neither of the two antibodies binds to recombinant E protein, the epitope of 1F4 could be mapped to a single E protein monomer, as displayed on the viral particle, and includes residues in EDI and the EDI–EDII hinge region (Figure 5b). The binding of 1F4 appeared to be dependent on the EDI–EDII hinge angle, which is conserved in E proteins on the virion surface but highly variable in recombinant E proteins. 1F4 is able to neutralise DENV1 not only by hindering interactions with its ancillary receptor DC-SIGN but also by preventing the E proteins from arranging into their post-fusion trimeric structure [232].

The DENV2-specific neutralising human mAb 2D22 [107,108] binds across E proteins within a dimer, making contacts with EDIII and the glycan loop of EDI in one subunit, and EDII, including the FL, in the other subunit [233] (Figure 5c). 2D22 blocks the E protein reorganisation necessary for fusion to occur by locking both ends of two thirds of or all dimers—depending on temperature and strain—on the viral particle. Its prophylactic and therapeutic activity against DENV2 has been demonstrated in vivo [233]. Two other human mAbs that potently inhibit DENV2 have been described: 1L12, which, based on competition assays, binds an epitope partially overlapping with that of 2D22, and 3F9, which engages an epitope centred on EDI that might represent a potential second major neutralising site on DENV2 [110,234].

5J7 is a human mAb that, though cross-reactive with all serotypes, specifically neutralises DENV3 at nanogram-range concentrations [107,108]. Its footprint spans three adjacent E proteins on the virion surface and involves the following polypeptides in the asymmetric unit: the EDI–EDII hinge region of molecule A, EDIII of molecule B and the tip of EDII of molecule B’, containing the FL (Figure 5d). This simultaneous binding of 5J7 to three E proteins enables full occupancy of the viral particle at only 60 copies, which is half the amount required by mAbs 14c10, 1F4, and 2D22 [231,232,233], in line with its higher neutralising potency [235].

Less attention had been paid to human antibodies that strongly neutralise DENV4. More recently, a set of DENV4-specific mAbs, D4-126 and D4-131, exhibiting potent neutralisation of several genotypes has been isolated from a single DENV4-immune subject [237]. Crystal structures of D4-126 and D4-131 in complex with DENV4 are yet to be resolved; however, both antibodies appear to target quaternary epitopes centred on the EDI–EDII hinge [237], consistent with the notion that this site contains serotype-specific determinants.

It is still unclear to which extent the integrity of these complex epitopes is affected by the structural heterogeneity of DENV, but the disruption of higher-order E protein structures on prM protein–containing virions certainly suggests lower reactivity. This should be investigated further.

#### 2.6.2. Antibody Responses to Serotype-Specific Quaternary Epitopes in Human Polyclonal Sera

While mAbs are powerful tools for epitope mapping, they are not representative of the polyclonal serum antibody response that mediates protection in humans. Partial exchange of epitopes between different serotypes using reverse genetics has been an effective strategy to measure serotype-specific antibodies in human polyclonal sera after natural infection and vaccination by gain or loss of neutralisation.

A recombinant DENV2 displaying the core of the DENV1 1F4 epitope (rDENV2/1) was used to screen sera collected after primary DENV1 infection [238]. Neutralising antibody titres to rDENV2/1 were significantly higher than the parental DENV2 titres, but significantly lower than those to the parental DENV1. Thus, the 1F4 epitope seems a major but not exclusive target of DENV1-specific neutralising antibodies, with additional quaternary epitopes on DENV1, such as the 14c10 epitope, potentially driving serotype-specific neutralisation [238]. Similar results were obtained when analysing neutralising antibodies induced by the monovalent rDEN1Δ30 vaccine [239].

The majority of neutralising antibodies in the sera of individuals that experienced primary DENV2 infection or that received the monovalent rDEN2/4Δ30 vaccine tracked with the 2D22 epitope displayed on a chimeric rDENV4/2, indicating that 2D22-like antibody responses are elicited by both natural infection and vaccination [114,234,239,240]. A smaller fraction of antibodies in these sera neutralised a chimeric rDENV4/2 containing core residues of the 3F9 epitope [234]. One study also observed reactivity of DENV2 immune sera with regions that did not map to the epitopes of mAbs 2D22 and 3F9, suggesting the presence of other neutralising sites on DENV2 [114].

To screen for DENV3-specific neutralising antibody responses, the 5J7 epitope has been transplanted into DENV1 and DENV4 backbones [239,241,242,243]. It was found that a proportion of neutralising antibodies in post-infection sera bound to the 5J7 epitope, suggesting that specific neutralisation of DENV3 is also mediated by antibodies directed to other sites on the virion [242,243]; indeed, a recent study has identified additional quaternary epitopes on DENV3 [244]. Interestingly, the chimeric DENV4/3 was not significantly neutralised by rDEN3Δ30 vaccine sera, demonstrating that the 5J7 epitope is not a major target of DENV3-specific neutralising antibodies following vaccination [239].

Using a chimeric rDENV4/3 that lacks the epitopes recognised by the DENV4-specific mAbs D4-126 and D4-131, a significant loss of neutralisation, relative to the parental DENV4, was observed for sera collected after primary DENV4 infection or rDEN4Δ30 vaccination [237,239]. This indicates that the binding sites of neutralising antibodies in natural infection sera and rDEN4Δ30 vaccine sera tightly overlap with the epitopes defined by mAbs to DENV4 [237,239].

Collectively, these studies demonstrate that DENV-neutralising antibodies in human polyclonal sera abundantly recognise serotype-specific quaternary epitopes, and that the antibody responses induced by the monovalent components of TV003/TV005—except for rDEN3Δ30—parallel those seen during natural infection. Future studies should determine the serum levels of these potent neutralising antibodies in proportion to those of enhancing FLE-specific and prM protein–specific antibodies following natural infection or immunisation with live-attenuated dengue vaccines, in order to define potential correlates of protection. The identification of the EDI–EDII hinge as a serotype-specific determinant in humans may aid the design of epitope mimetic peptide vaccines, which would avoid the induction of undesirable antibody responses associated with live-attenuated dengue vaccines.

#### 2.6.3. Antibodies that Bind the E Protein Dimer Epitope

Besides serotype-specific neutralising antibodies that target higher-order E protein structures, a class of broadly neutralising human mAbs has been described that bind a conformational epitope at the interface between the two subunits of each E protein dimer, termed the E protein dimer epitope (EDE) [71]. The EDE is highly conserved across the four serotypes because it is the interaction site of the prM protein during the virion maturation process in the TGN of the infected cell [63].

EDE-specific mAbs are divided into two subclasses based on their sensitivity to N-linked glycosylation at position 153 of the E protein, which is required by EDE2-specific mAbs but not by EDE1-specific mAbs [71]. Their footprints on the E protein dimer are as follows: on one subunit, both antibody subclasses target the same regions of EDII, in particular residues in the b strand (containing the Asn67-linked glycan), the FL and upstream residues, and the ij loop; on the other subunit, EDE2-specific mAbs bind the ‘150 loop’ of EDI and its Asn153-linked glycan, whereas EDE1-specific mAbs engage residues in EDI and EDIII and displace the 150 loop, allowing additional interactions with the conserved A strand of EDIII [236] (Figure 5e,f). The epitope of the DENV2-specific mAb 2D22 partially overlaps with that of EDE1-specific mAbs; however, it is shifted more towards EDIII (compare Figure 5c,e), accounting for the differential neutralisation breadth [233].

In contrast to FLE-specific mAbs, mAbs to EDE were found to potently block the infectivity of fully mature virions produced in primary human cells [71] or circulating in patients [131]. In addition, it was found that EDE-specific mAbs are fully capable of neutralising bumpy DENV2 particles [136]. EDE-specific mAbs were also shown to efficiently neutralise dengue virions with prM protein contents greater than 60% [71], presumably by displacing uncleaved prM proteins from the prM–E trimers and trapping the E proteins as dimers, in a process called conformational selection [71,236]. Interestingly, EDE-specific mAbs were able to outcompete FLE-specific mAbs for the binding to low prM protein–containing, but not high prM protein–containing, DENV particles [245], suggesting a role for these antibodies in reducing the risk posed by infection-enhancing antibodies directed to the FLE. Like the majority of DENV-neutralising antibodies, however, EDE-specific mAbs were also shown to cause ADE at sub-neutralising concentrations, though not as potently as FLE-specific mAbs [71].

Neutralisation by EDE-specific mAbs appears to extend beyond the DENV serocomplex, with studies reporting cross-neutralisation of the closely related ZIKV [246,247]. In this context, notable differences between EDE1-specific mAbs and EDE2-specific mAbs have been observed. EDE1-specific mAbs were found to neutralise ZIKV more strongly than EDE2-specific mAbs [151,246]; the latter, in turn, potently enhanced ZIKV infection over a wide range of concentrations [151]. Furthermore, EDE1-specific mAbs, but not EDE2-specific mAbs, could override ADE of ZIKV infection induced by polyclonal DENV immune sera [151] and the prophylactic or therapeutic administration of EDE1-specific mAbs could confer protection against ZIKV in vivo [247,248,249]. The protective efficacies of EDE1-specific mAbs against DENV infection are yet to be demonstrated in animal models, though certainly warranted given the promising findings of studies with ZIKV. Nonetheless, EDE1-specific mAbs represent a class of antibodies with favourable characteristics that should be elicited by next-generation vaccines.

#### 2.6.4. E Protein Dimer–Based Vaccine Candidates

Multiple dengue vaccine candidates based on monomeric E proteins or prM–E heterodimers, which are readily produced in various expression systems, have been described (reviewed in [250]). Generating dimeric E proteins, as they are found on viral particles, for vaccination purposes is however more challenging. Recombinant soluble E (sE) protein, lacking the stem region and C-terminal transmembrane anchor, crystallises as a dimer [41,43] but is mainly monomeric in solution. Interestingly, E protein dimer–dependent mAbs are able to drive the dimerisation of DENV sE proteins [236,245,251], allowing subsequent capture on biological matrices. Alternatively, sE protein dimers can be covalently stabilised by introducing cysteine substitutions at opposing residues [245,252]. Mutants connected via a single disulphide bond at the centre of the sE protein dimer (e.g., DENV2 sE A259C) are considerably dynamic, being able to rotate about the engineered bond, and thereby allow for the exposure of the FLE [245,252]. In contrast, mutants ‘locked’ by two inter-subunit disulphide bonds located at each end of the dimer (e.g., DENV2 sE L107C/A313C) do not expose the FLE but are efficiently recognised by EDE-specific mAbs [245]. Moreover, expression of the immunogens is possible in the absence of prM proteins, thus removing another undesirable antibody target. Locked E protein dimers may therefore be promising vaccine candidates, capable of inducing highly potent, broadly neutralising antibodies while largely eliminating the risk of ADE. Ideally, the immunogens were to be delivered in a format closely resembling the structural architecture of DENV particles, in order to avoid the induction of irrelevant antibodies to epitopes that are normally cryptic in the virion. Thus far, data on their immunogenicity or efficacy have not been published.

## 3. Antibodies to NS1

### 3.1. Structure and Pathogenic Roles of NS1

NS1 plays an important role in DENV replication, immune evasion and pathogenesis (reviewed in [253]). The protein is initially synthesised as a monomer that rapidly dimerises following post-translational modification in the lumen of the ER [254]. Each monomer is composed of three domains: a small β-roll domain (aa 1–29), the ‘wing’ domain (WD; aa 30–180), and a central β-ladder domain comprising the C-terminal half of NS1 (aa 181–352) [255]. The dimeric form of NS1 either becomes associated with organelle or cell membranes [256,257], or further assembles into a soluble hexamer that is secreted from infected cells [258,259]. Secreted NS1 (sNS1) circulates in the bloodstream of DENV-infected individuals where it is detectable throughout the entire febrile phase as well as on the first days of convalescence [259,260]. The plasma concentration of sNS1, often exceeding several micrograms per millilitre, has been shown to correlate with disease severity in dengue patients [261]. In fact, recent studies have demonstrated that sNS1 is directly involved in DENV-induced endothelial dysfunction and vascular hyperpermeability. Firstly, sNS1 activates Toll-like receptor 4 signalling in peripheral blood mononuclear cells (PBMCs) leading to the release of vasoactive mediators, such as interleukin-6 (IL-6) and tumour necrosis factor–alpha (TNF-α) [262,263]. Secondly, sNS1 binds to and is internalised by endothelial cells triggering degradation of the endothelial glycocalyx layer and disruption of intercellular junctions [262,264,265,266], independent of inflammatory cytokines [267]. Moreover, sNS1 appears to interfere with the coagulation cascade [268] and to induce platelet activation and apoptosis [269], thereby potentially contributing to haemorrhage and thrombocytopenia during DENV infection.

### 3.2. NS1-Specific Antibodies and Their Protective Effects

NS1 is highly immunogenic, with a considerable fraction of the human antibody response to DENV infection directed to this protein [70,105,111,117]. NS1-specific antibodies are detected in convalescent sera following primary infection, and in sera collected during the acute and convalescent phases of secondary infection [111,270,271,272,273,274,275]. Their levels are usually higher after secondary infection [104,270,271,272,273,276] but not significantly different between DF and DHF/DSS patients [270,271,272,273,274]. Moreover, a large proportion of NS1-specific antibodies in secondary infection sera is cross-reactive [70,105,117,275]—by virtue of high conservation of NS1 across the four serotypes [277]—, however, in contrast to most cross-reactive antibodies to the prM and E proteins, antibodies to NS1 are not implicated in ADE of heterologous infection, simply because their target is not virion-associated.

At least six antigenic regions of NS1—the majority of which contains linear, surface-exposed epitopes—have been identified in both NS1-immunised mice and DENV-infected mice as well as in naturally infected humans (see [253] for a thorough summary). A region comprising the disordered distal tip of the NS1 WD (aa 108–128) is worth mentioning, as it was found to be immunodominant in both species, and to induce antibodies that cross-react with NS1 from all serotypes [274,275,278,279,280,281,282]. Some studies also suggest conformational epitopes to be present on NS1 [275,283,284,285,286]. Little is, however, known about antibodies that recognise epitopes spanning two monomers in the NS1 dimer or that bind across dimers in the hexameric form of NS1. Sera of DENV-immune donors should be screened for the presence of such antibodies, and their functional roles should be determined.

**Figure 6:**
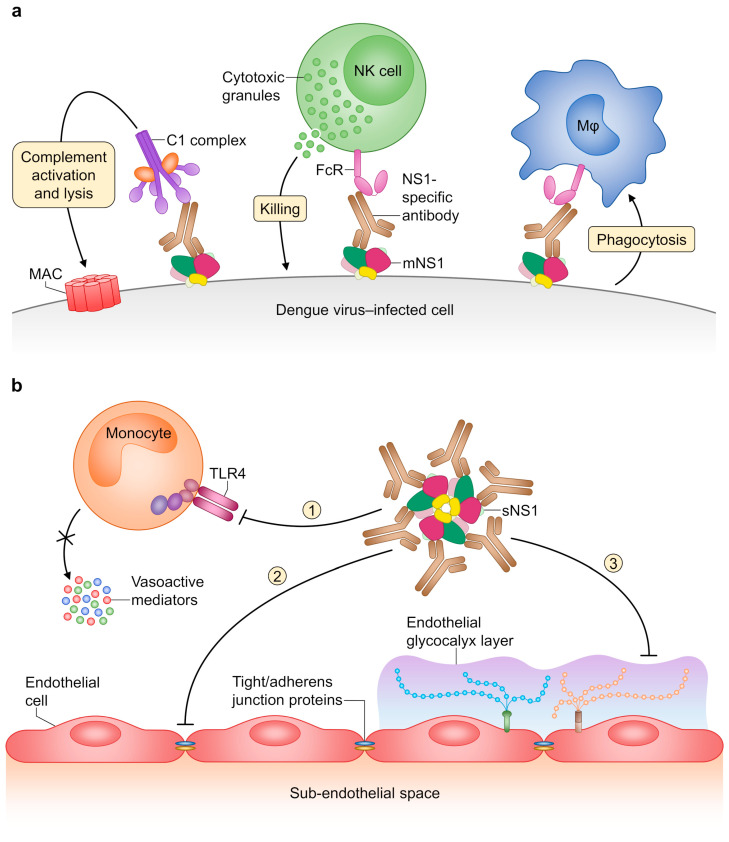
Proposed mechanisms of NS1-specific antibody–mediated protection. (**a**) Antibodies that recognise membrane-associated NS1 (mNS1) may promote lysis and clearance of DENV-infected cells by activating complement or recruiting effector cells via their Fc portion. (**b**) Antibodies that bind secreted NS1 (sNS1) in circulation may protect against NS1-induced endothelial hyperpermeability by preventing (1) the activation of innate immune cells and subsequent release of vasoactive mediators, (2) the disruption of endothelial intercellular junctions, and/or (3) the degradation of endothelial glycocalyx layer components. Membrane attack complex (MAC); macrophage (Mφ); natural killer (NK); Toll-like receptor 4 (TLR4).

Immunisation with purified NS1 [102] or passive transfer of NS1-specific antibodies [287] was shown to prevent lethal DENV2-induced encephalitis in mice. The protective effects of DENV NS1–specific antibodies were initially attributed to their ability to promote complement-mediated cytotoxicity, based on prior experiments with YFV NS1–specific antibodies [288] and the finding that antibodies unable to fix complement did not confer protection [102,287]. More recently, antibody recognition of membrane-associated NS1 has been shown to activate additional Fc-mediated effector functions, namely antibody-dependent cellular cytotoxicity [289,290] and antibody-dependent cellular phagocytosis [291], contributing to both lysis and clearance of infected cells (Figure 6a). Furthermore, NS1-specific antibodies also bind sNS1 in circulation and neutralise its vasoactive effects, as demonstrated in a mouse model of NS1-induced vascular leakage [262] (Figure 6b). Given the multiple roles of NS1 in dengue pathogenesis, additional studies are needed to delineate the exact mechanisms of protection mediated by NS1-specific antibodies.

### 3.3. Vaccine Candidates Based on Full-Length NS1

Over the past two decades, several groups have further explored the potential of NS1 as a vaccine candidate. Mice immunised with a recombinant vaccinia virus expressing NS1 of DENV2 or DENV4 showed complete survival upon intracerebral challenge with the homologous serotype [292]. DNA vaccines encoding NS1 fused to its natural signal sequence [293,294], derived from the C terminus of the E protein [295], or fused to the secretory signal sequence of human plasminogen activator [284,296] induced strong antibody responses and were highly protective in mice. Furthermore, NS1 vaccination in the presence of non-traditional adjuvants, such as a non-toxic heat-labile toxin derivative (LT_G33D_) [285] or monophosphoryl lipid A [274], resulted in increased protective efficacy than was observed with traditional adjuvants.

Though these NS1-based vaccine candidates protected against lethal infection with the homologous DENV serotype, cross-protection against challenge with heterologous serotypes was either not investigated or not observed. Recently, it has been demonstrated that vaccination with NS1 of DENV1, 3 or 4 could confer substantial protection against lethal DENV2 challenge in mice, which was found to be associated with high NS1-specific antibody titres [262,297]. Moreover, sera of individuals vaccinated with TAK-003, which also encodes DENV2 NS1 [298], not only prevented DENV2 NS1–induced human endothelial cell hyperpermeability, but also exerted strong cross-inhibitory effects against hyperpermeability caused by NS1 of the other serotypes [299]. Importantly, CYD-TDV, the only licenced dengue vaccine, does not generate DENV NS1–specific antibodies [300], which might be another explanation for the low-level protection observed in efficacy trials.

### 3.4. Molecular Mimicry between NS1 and Host Proteins

Despite the ability to induce protective antibody responses against DENV infection, NS1 also elicits antibodies cross-reactive with host self-antigens via molecular mimicry that might contribute to dengue pathogenesis. NS1-specific mouse mAbs were found to bind to human endothelial cells and coagulation factors in vitro, and to cause haemorrhage in mice, indicative of potentially detrimental properties [301]. Investigations using sera of DHF/DSS patients eventually provided a link between NS1-specific antibodies and DENV-induced vascular leakage [302]. It was demonstrated that antibodies in these sera cross-reacted with human endothelial cells and mediated their lysis in the presence of complement; and that this could be abolished by pre-absorption with recombinant NS1. Other effects found to be exerted by NS1-specific antibodies on human endothelial cells include the induction of apoptosis [302,303] and inflammatory activation [304]. In addition, some studies suggest a role for NS1-specific antibodies in thrombocytopenia and coagulopathy. This is partly due to their binding affinity for platelets, which either targets them for phagocytosis by macrophages [305], or inhibits their aggregation [306,307] causing prolonged bleeding times, at least in mice [307]. Furthermore, NS1-specific antibodies activate plasminogen and enhance its conversion to plasmin [308,309,310]; and likewise, recognition of thrombin by NS1-specific antibodies has been shown to prevent fibrin formation [309]. Finally, increased serum aminotransferase levels in mice passively transferred with antibodies from DENV-infected individuals or NS1-immunised mice [311] indicate that NS1-specific antibodies might also contribute to the hepatic damage observed in dengue patients. While the results of in vitro and mouse experiments point towards a direct involvement of NS1-specific antibodies in dengue pathogenesis, their role remains controversial in humans. For one, NS1-specific immunoglobulin G (IgG) is still circulating in patients long after symptom resolution without indications of autoimmune sequelae. In contrast, other studies suggest that autoreactive antibodies of the short-lived IgM isotype might be responsible for the observed pathogenic effects [312,313]. Further studies in dengue patients are needed to clarify whether NS1-specific antibodies that cross-react with host molecules are indeed driving disease progression.

**Figure 7:**
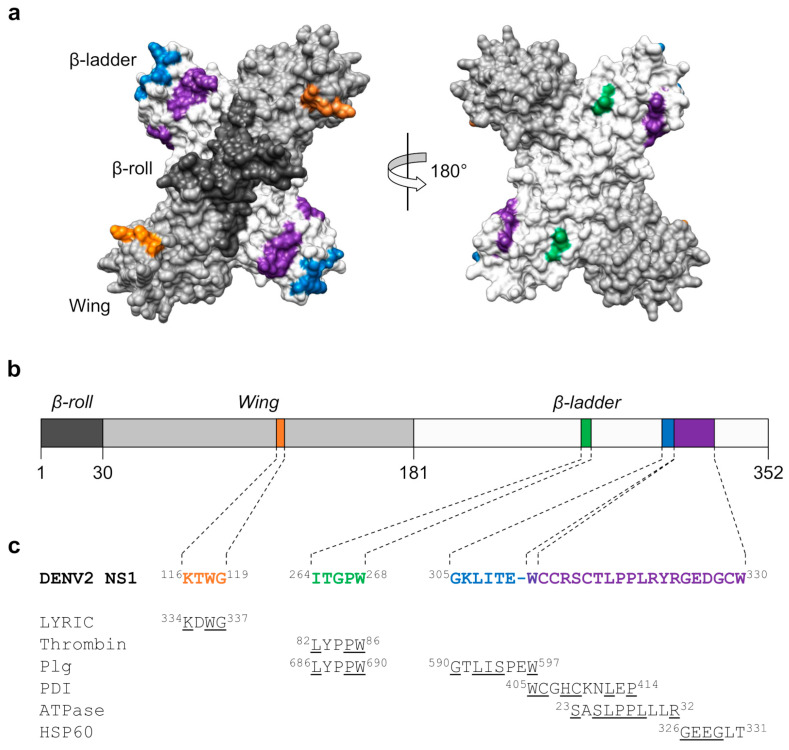
Antigenic determinants of molecular mimicry between NS1 and self-antigens. (**a**) Homology model of the DENV NS1 dimer highlighting the locations of cross-reactive epitopes. Based on the DENV2 NS1 structure (PDB 4O6B) with missing residues modelled according to the Zika virus (ZIKV) NS1 structure (PDB 5GS6). The model was built using the SWISS-MODEL server [317] and molecular graphics were prepared with UCSF Chimera [75]. The NS1 domains β-roll, wing and β-ladder are depicted in dark grey, light grey and white, respectively. (**b**) Schematic representation of DENV NS1 showing the positions of cross-reactive epitopes. (**c**) Alignment of partial amino acid sequences of DENV2 NS1 and the self-antigens recognised by cross-reactive NS1-specific antibodies. Amino acid residues in these proteins thought to be bound by NS1-specific antibodies are underlined. Lysine-rich CEACAM1 co-isolated (LYRIC); plasminogen (Plg); protein disulphide isomerase (PDI); heat shock protein 60 (HSP60).

Efforts were undertaken to identify the molecular determinants of host cross-reactivity (see Figure 7), in order to obviate such undesirable responses being induced by future NS1 subunit vaccines. Antibodies generated against ELK/KLE-type motifs in NS1 were shown to bind to similar epitopes in host molecules [280,301]. In addition, several host cross-reactive epitopes have been found in the C-terminal region of NS1 (aa 271–352) [307,314]. In particular, amino acid residues 311–330 of NS1 form an immunodominant region that shares sequence homology with a number of proteins expressed on human endothelial cells or platelets, such as protein disulphide isomerase, ATPase, and heat shock protein 60 [306,315]. In addition, two epitopes suggested to be responsible for antibody cross-reactivity with human coagulation factors have been mapped to amino acid residues 264–268 and 305–311 of NS1 [308,309,310]. Furthermore, a KXWG motif located in the immunodominant disordered loop of the NS1 WD (aa 116–119) has been shown to induce antibodies that cross-react with lysine-rich CEACAM1 co-isolated (LYRIC) on human endothelial cells [316].

### 3.5. Modified NS1-Based Vaccines

The identification of epitopes that induce antibodies cross-reactive with self-antigens eventually allowed for the design of alternative NS1-based vaccine candidates. Antibodies generated against modified forms of DENV NS1, in which the cross-reactive C terminus (aa 271–352) was either removed or replaced by the corresponding region of JEV NS1, showed lower binding affinity for human endothelial cells and platelets; furthermore, these antibodies reduced viral load and haemorrhage in the skin of DENV2-infected mice [290,318]. Another group produced a truncated form of DENV2 NS1 (DENV2 ΔNS1) containing deletions of two known cross-reactive epitopes (aa 116–119 and 311–330) and one potentially cross-reactive region (aa 221–266) [319]. No binding to human endothelial cells and platelets was observed for antibodies induced by DENV2 ΔNS1, and mice immunised with this vaccine candidate were considerably protected against a lethal DENV2 challenge [319]. The most promising results, thus far, were obtained using a modified NS1-WD peptide, which lacks the KXWG motif and an ELK/KLE-type motif, as immunogen [320]. Modified NS1-WD peptide immune sera recognised NS1 from all four DENV serotypes in the absence of cross-reactivity with human endothelial cells. Beyond that, both passive and active immunisation conferred nearly full protection against DENV-induced disease and mortality. The group also found that antibodies recognising this modified NS1-WD peptide are formed naturally during human DENV infection and that their relative titres are inversely correlated with disease severity. It was therefore concluded that such antibodies are also an important part of the protective immune response in humans [320].

## 4. T-Cell Responses to DENV Infection and Vaccination

### 4.1. DENV Proteins Recognised by T Cells

Comprehensive knowledge of the functional properties and antigen specificity of DENV-specific T cells is crucial to understand their role in the immune response to DENV infection.

Earlier studies identified especially NS3 as a target for virus-specific T-cell responses [33,321,322,323,324,325,326,327,328,329], in addition to a few antigenic regions located in other DENV proteins [330,331,332,333,334,335,336,337,338,339]. In order to define the specificity of the T-cell responses more precisely, overlapping peptide sets encompassing the entire viral proteome or prediction of epitopes based on binding affinity for a given human leukocyte antigen (HLA) molecule have been used. Using overlapping peptides spanning the DENV2 proteome, the T-cell response in Thai paediatric dengue patients was shown to be directed to NS3 most frequently, followed by NS5, E, and NS1 [34], while in adult dengue patients from Singapore, CD8^+^ T-cell responses were mainly directed to NS3 and NS5, and CD4^+^ T-cell responses to the C and E proteins and to a lesser extent to NS1 [340]. In another study, healthy blood donors from Sri Lanka were screened for T-cell reactivity against pools of predicted HLA-matched peptides covering all four serotypes of DENV, which revealed that virus-specific CD8^+^ T cells were predominantly directed to NS3, NS4B, and NS5 [35]. Subsequent analysis indicated serotype-dependent immunodominance hierarchy of DENV proteins. Specifically, DENV1, 2, and 4 elicited CD8^+^ T cells recognising the non-structural proteins predominantly, whereas DENV3-specific responses were found to be equally directed to structural and non-structural proteins [341], which confirmed previous findings in HLA-transgenic mice [342]. These studies were repeated in a Nicaraguan cohort, resulting in a DENV-specific ‘mega-pool’ of 268 CD8^+^ T-cell epitopes. Its reactivity was further validated in an independent cohort from Brazil, supporting global applicability [341]. The same approach was later used to map DENV-specific CD4^+^ T-cell responses restricted by common HLA-DRB1 alleles in the Sri Lankan [343] and Nicaraguan cohorts [344]. The C protein was found to be the most immunodominant target, followed by NS3, NS2A, and NS5 [343,344] and a mega-pool of 180 CD4^+^ T-cell epitopes was defined with broad reactivity in various cohorts of DENV-exposed donors worldwide [344]. Finally, a number of studies have pointed out that sequential exposures to DENV tend to skew T-cell responses towards recognition of highly conserved epitopes [33,34,35,340,342].

**Figure 8:**
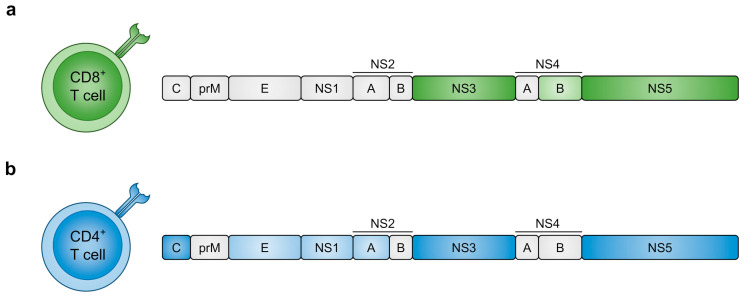
Immunodominant targets of DENV-specific T cells. Schematic highlighting the DENV proteins preferentially recognised by CD8^+^ T cells (**a**) and CD4^+^ T cells (**b**), where higher colour intensity indicates higher response frequency.

Collectively, the immunodominance patterns of DENV-specific CD8^+^ and CD4^+^ T cells have been identified (Figure 8)—which may guide rational vaccine design—and have provided the tools needed for dissecting T-cell responses to natural infection and vaccination.

### 4.2. Involvement of T Cells in Dengue Pathogenesis

The role of T cells during DENV infection has been subject to debate over the last few decades. Elevated serum levels of pro-inflammatory mediators (‘cytokine storm’) [345,346,347]—suspected to contribute to vascular permeability—increased expression of the early activation marker CD69 on CD8^+^ T cells [348], and higher frequencies of circulating DENV-specific CD8^+^ T cells [323] in patients with DHF than in those with DF initially supported a link between the T-cell response and disease severity. In addition, evidence was provided for T-cell original antigenic sin in the pathogenesis of severe dengue disease [33]. Using double tetramer staining for serotype-defined variants of the HLA-A*11:01–restricted NS3_133–142_ epitope, it was shown that the majority of epitope-specific CD8^+^ T cells in secondary DHF patients bound to tetramers loaded with peptides derived from the primary infecting serotype [33]. Furthermore, a considerable proportion of these cells cross-reacted with tetramers containing peptides of the currently infecting serotype; however, the affinity of their T-cell receptor (TCR) for these heterologous peptide–HLA tetramer complexes was shown to be significantly lower [33]. It was therefore proposed that cross-reactive CD8^+^ memory T cells may be selectively expanded during secondary DENV infection but that their low avidity for the heterologous infecting serotype may result in less efficient viral clearance, leading to increased viral burden and immunopathology [33]. Subsequent studies found that cross-reactive CD8^+^ T cells stimulated with heterologous peptides displayed altered cytokine profiles [34,321,325,328,336,337]. In particular, these cells produced high levels of pro-inflammatory cytokines, such as TNF-α, but did not markedly degranulate—as defined by CD107a expression—in patients with severe dengue disease [34,328], whereas others observed unaltered cytolytic activity but impaired interferon-gamma (IFN-γ) secretion and proliferative capacity [321]. Another study established a direct correlation between the strength of TCR stimulation and the types of cytokines produced by cross-reactive CD8^+^ T cells [325]. Weak TCR stimulation by variant peptides was found to initiate only CC-chemokine ligand 4 (CCL4) production, whereas the production of IFN-γ required strong TCR stimulation by the cognate peptide [325], providing a possible explanation for the observed qualitative changes in the cytokine responses to heterologous DENV serotypes.

Although not only HLA class I alleles [333,349,350,351,352,353] but also HLA class II alleles [350,351,354] have been associated with increased susceptibility to the severe forms of dengue, fewer studies had assessed pathogenic effects of CD4^+^ T cells. In one report, DENV-specific CD4^+^ T cells were found to lyse non-infected bystander cells and to secrete pro-inflammatory cytokines when pulsed with heterologous antigens [355]. Others observed only limited cytotoxic potential but high-level production of vasoactive mediators [34]. Similarly, it was demonstrated that stimulation of DENV-specific CD4^+^ T cells with heterologous peptides resulted in higher ratios of TNF-α to IFN-γ than were measured for homologous peptides [356,357], which was found to be associated with an increased risk of hospitalisation [356].

### 4.3. Protective Role of T Cells During DENV Infection

#### 4.3.1. Evidence from Murine Studies

Although various human studies suggest that DENV-specific T cells contribute to immunopathology, it has been difficult to reproduce these observations in experimental animals. Only two reports exist in which the inoculation of mice with DENV-specific CD8^+^ T cells had some pathogenic effects during a subsequent viral challenge [358,359]. In contrast, several murine studies have established the importance of T cells in controlling DENV infection and preventing severe outcomes. Mice deficient in CXC-chemokine receptor 3 (CXCR3) or its ligand, CXCL10, that received an intracerebral DENV2 challenge showed significantly reduced cerebral infiltration by T cells, especially by those of the CD8^+^ subset, which was accompanied by higher viral loads in the brain and lower survival rates [360]. Others have reported that mice were not protected against lethal DENV2 challenge in the absence of T cells, despite high levels of neutralising antibodies [361]. In addition, DENV-specific CD8^+^ T cells were shown to be essential for protection against viral challenge in mice, as depletion of these cells prior to infection led to significantly increased viral burden [103]. Furthermore, DENV-specific CD8^+^ T cells prevented antibody-induced severe dengue disease in mice [362]. Moreover, adoptive transfer of T cells from DENV1- or DENV4-immune mice to naïve recipient mice prior to a heterologous DENV2 challenge resulted in lower viral burden and mortality [363,364], suggesting a role for DENV-specific T cells in cross-protection.

#### 4.3.2. Evidence from Human Studies

The findings of mouse studies are complemented by data from immunological evaluations of DENV-infected individuals. Of note, a recent report of persistent DENV3 infection in a lymphopenic renal transplant patient stated that, despite consistently detectable levels of neutralising antibodies, resolution of infection was only observed when CD8^+^ T-cell counts increased to the lower normal limit [365]. In a paediatric cohort, both activation of canonical signalling pathways and expression of surface markers associated with an activated T-cell phenotype were increased in asymptomatic viraemic individuals as compared to clinical dengue patients [366]. Another group reported that the expansion of CD8^+^ effector memory T-cell subsets correlated with decreased viral loads in acute dengue patients [367]. Moreover, early appearance of IFN-γ–producing DENV-specific T cells in dengue patients was found to be associated with milder clinical disease and the resolution of viraemia [368]. In another study, higher frequencies of DENV-specific CD4^+^ and CD8^+^ T cells producing IFN-γ, TNF-α, and IL-2 were observed in children who subsequently developed subclinical secondary infection than in those who later experienced symptomatic secondary infection [369]. Furthermore, memory T cells of patients with past mild/subclinical DENV infection were found more likely to produce only the cytotoxic molecule granzyme B after NS3-specific peptide stimulation than those of patients who were hospitalised due to dengue [370]. Recently, it has been shown that NS3- and NS5-specific CD8^+^ T cells expressing multiple cytokines were more frequent in patients with DF than in those with DHF. In addition, the production of IFN-γ and TNF-α by these cells was significantly higher in DF patients [371].

Several observations specifically challenge the idea that dominant cross-reactive CD8^+^ T-cell responses, resulting from original antigenic sin, are strictly associated with the pathogenesis of secondary heterologous infection. One study found no increased CD8^+^ T-cell activation in the blood of DHF patients prior to the commencement of haemoconcentration [372]. This temporal mismatch was further confirmed by the late appearance of CD8^+^ T cells that stained positive for HLA-A*11:01 tetramers containing serotype-defined variants of the NS3_133–142_ epitope, suggesting a negligible role of these cells in triggering plasma leakage [372]. Using the same pool of tetramers, it was found that CD8^+^ T cells are cross-reactive regardless of DENV infection history and that tetramer-positive CD8^+^ T-cell frequencies did not correlate with disease severity in the patients studied [324]. In contrast to previous studies [33,34,328], it was demonstrated that NS3_133–142_-specific CD8^+^ T cells in patients with acute secondary DENV infection did not show any functional impairment, as these were highly activated and proliferating in vivo, and exhibited antiviral effector functions such as high-level production of IFN-γ and expression of the degranulation marker CD107a ex vivo [330]. Importantly, analysis of the CD8^+^ memory T-cell responses to pools of peptides representing serotype-specific and conserved epitopes in donors from hyperendemic regions, showed no appreciable difference in TCR avidity and cytokine expression patterns [35].

An interesting finding has been that individuals expressing HLA class I variants associated with reduced susceptibility to severe dengue disease, such as HLA-B*35:01 [350], generated CD8^+^ T-cell responses of higher magnitude and greater multi-functionality, supporting an HLA-linked protective role for DENV-specific CD8^+^ T cells [35]. The same correlation was also observed in another dengue-endemic population [373]. In addition, it was found that the majority of DENV-specific IFN-γ–producing CD8^+^ T cells in these study subjects were either effector memory (T_EM_, CCR7^−^CD45RA^−^) or terminally differentiated memory cells (T_EMRA_, CCR7^−^CD45RA^+^) [373]. Interestingly, these cells showed significant up-regulation of programmed cell death protein-1 (PD-1) in HLA-B*35:01–positive individuals, but not in those expressing the disease-susceptible allele HLA-A*24:02. Although PD-1 expression is a known marker of T-cell exhaustion, DENV-specific PD-1^+^CD8^+^ T cells demonstrated only low levels of other co-inhibitory receptors, such as CTLA-4 or TIM-3, and were not impaired in their proliferative capacity and cytotoxic function [373]. This suggests that the expression of PD-1 on CD8^+^ memory T cells is associated with antiviral effector functions rather than exhaustion and, thus, may serve as a potential correlate of protection.

Moreover, several HLA class II alleles have been associated with increased resistance to severe dengue disease [343,349,351,354]. For example, it was observed that subjects positive for certain HLA-DRB1 alleles had a reduced risk of hospitalisation due to dengue, which correlated with higher-magnitude CD4^+^ T-cell responses [343]. Specifically, a population of CD4^+^ T_EMRA_ cells was found to be expanded in healthy HLA-DRB1*04:01–positive individuals with a history of recurrent exposure to DENV [374]. Interestingly, CD4^+^ T_EMRA_ cells responding to DENV-specific stimulation did not express markers of conventional T helper cell subsets. Instead, these cells displayed a cytotoxic phenotype, up-regulated CX3-chemokine receptor 1 (CX3CR1), and mediated direct cytolytic activity ex vivo [374]. Further characterisation of this CD4^+^ T_EMRA_ population revealed that cells positive for the adhesion molecule G protein–coupled receptor 56 (GPR56), in particular, showed enhanced expression of cytotoxicity-related molecules such as CD244, perforin, and granzyme B [375,376]. Moreover, among subjects with secondary DENV infections, CD4^+^ T_EMRA_ cells that produced IFN-γ after antigenic stimulation predominantly exhibited a GPR56^+^ phenotype [375], corroborating the involvement of cytotoxic CD4^+^ T_EMRA_ subsets in HLA-dependent protection against severe dengue disease.

The protective effects of DENV-specific T cells may also depend on their ability to traffic to the skin, where DENV infection is initiated following a mosquito bite. A study analysing PBMCs of acute dengue patients showed that CC-chemokine receptor 5 (CCR5) and CXCR3, which direct migration to inflamed tissues, as well as the skin-homing receptor cutaneous lymphocyte-associated antigen (CLA) were up-regulated on NS3_133–142_-specific CD8^+^ T_EM_/T_EMRA_ cells [330]. Furthermore, CLA expression on circulating DENV-specific CD4^+^ and CD8^+^ T cells appeared to correlate with their skin-homing capacity, as these cells were detected at high frequencies in the skin of patients with acute dengue. In addition, virus-specific T cells isolated from skin blister fluid of these patients were found to produce IFN-γ after peptide stimulation, indicating their capability to exert antiviral functions in vivo [330]. Whether DENV-specific CLA-expressing T cells eventually differentiate into tissue-resident memory T cells in the skin, forming a first line of defence against reinfection [377], should be investigated further.

**Figure 9:**
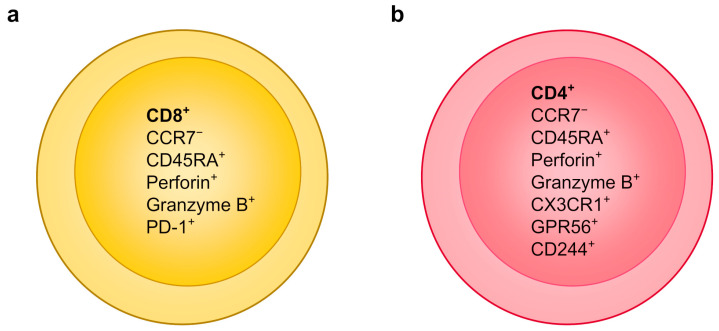
Memory T-cell phenotypes potentially associated with protection against severe dengue disease. Schematic depicting the markers expressed by cytotoxic CD8^+^ T_EMRA_ cells (**a**) and cytotoxic CD4^+^ T_EMRA_ cells (**b**) in DENV-exposed individuals carrying protective human leukocyte antigen (HLA) alleles. CC-chemokine receptor 7 (CCR7); CX3-chemokine receptor 1 (CX3CR1); G protein–coupled receptor 56 (GPR56); programmed cell death protein-1 (PD-1).

Overall, the findings of the studies discussed here strongly support the role of T cells in protection against (severe) dengue disease (Figure 9), thus, emphasising that the induction of potent antiviral T-cell responses should be a goal of dengue vaccines.

### 4.4. Importance of DENV-Specific T Cells in Vaccine-Mediated Protection

#### 4.4.1. T-Cell Responses to Live-Attenuated Dengue Vaccines

With most T-cell epitopes located in the C and non-structural proteins, which in the case of CYD-TDV are provided by its YFV-17D backbone, the induction of T-cell responses by this vaccine largely depends on T-cell cross-reactivity between YFV and DENV. However, CYD-TDV vaccination was shown to elicit CD4^+^ and CD8^+^ T cells specific for YFV NS3 but not DENV NS3 in flavivirus-naïve individuals [378,379], and in DENV-immune recipients, this vaccine did not significantly boost pre-existing DENV NS3–specific CD4^+^ or CD8^+^ T cells [378,379], indicating the absence of cross-reactivity between YFV/DENV NS3–specific T-cell responses. Importantly, it has recently been demonstrated that CD8^+^ T-cell responses induced by the YFV-17D vaccine are capable of recognising DENV-derived epitopes, but that these cross-reactive responses are significantly lower than those to homologous YFV-derived epitopes in terms of both magnitude and activation capacity [380]. Moreover, CD4^+^ and CD8^+^ short-term T-cell lines from YFV-17D vaccinees were shown to exhibit very limited cross-reactivity with DENV, and in the majority of cases more than 1000-fold lower antigen sensitivity [380]. This suggests that the YFV-17D backbone of CYD-TDV is unlikely to generate optimal T-cell responses to DENV, which might explain the low protective efficacy of this vaccine. Moreover, it is tempting to speculate that the lack of DENV-specific T-cell responses in seronegative vaccinees plays a role in the increased risk of severe dengue disease observed for this subgroup.

T-cell responses elicited by TAK-003 were found to be directed to both the structural proteins of DENV1–4 and the non-structural proteins of the DENV2 backbone, thus, spanning the entire DENV proteome [381]. TAK-003–induced CD4^+^ T cells have not yet been characterised, but CD8^+^ T cells were shown to be multi-functional and to target NS1, NS3, and NS5 of the DENV2 backbone, with a large proportion also being cross-reactive with NS3 of the other three serotypes [382]. The overall magnitude of the cross-reactive T-cell response to the non-structural proteins of DENV1, DENV3, and DENV4, however, appeared to be significantly lower than the magnitude of the response to the DENV2 backbone [381]. Whether this is a potential factor contributing to the lower protective efficacy of TAK-003 against DENV1 and DENV3 [92,93] has yet to be investigated.

In the case of TV003/TV005, administration of its monovalent vaccine components was found to induce serotype-specific CD8^+^ T-cell responses targeting both structural and non-structural proteins. Following tetravalent vaccination, however, the CD8^+^ T-cell response is skewed toward epitopes of the non-structural proteins that are highly conserved across the four serotypes, including a large number of field isolates [383]. These patterns of T-cell recognition appeared to be very similar to those observed after natural infection [341]. Furthermore, CD4^+^ T-cell responses were shown to be focussed mainly on the C protein, NS2A, and NS5 in individuals that received TV005. Moreover, the vaccine-induced CD4^+^ T-cell responses closely resembled those seen in humans naturally exposed to DENV, in terms of magnitude, antigen specificity, and functional properties [384]. Recently, it has been reported that the generation of multi-functional CD4^+^ and CD8^+^ T_EMRA_ cells after TV003 vaccination correlated with protection in a controlled human DENV2 infection model, providing additional evidence for the role of these DENV-specific T-cell subsets in protective immunity [385].

#### 4.4.2. T-Cell Responses to Experimental Dengue Vaccines

Over the past decade, various vaccine strategies specifically aiming at the induction of protective T-cell responses have been investigated. Peptide immunisation with dominant CD4^+^ or CD8^+^ T-cell epitopes prior to a DENV2 challenge significantly enhanced viral clearance in mice [103,386]. Depletion and adoptive transfer experiments showed that CD8^+^ T cells, induced by vaccination with a viral replicon particle expressing the DENV2 E protein ectodomain, were able to reduce viral load upon challenge, even in the presence of enhancing antibodies [387]. Moreover, a recombinant vaccine based on the immunodominant C protein of DENV2 was able to induce protective T-cell responses in non-human primates, without the contribution of neutralising antibodies [388]. One group tested various DNA-based vaccine candidates encoding either NS3 or one of its two functional domains, protease and helicase, respectively. Mice immunised with NS3 or the NS3 helicase domain developed high frequencies of NS3-specific IFN-γ–secreting CD8^+^ T cells and were considerably protected against a DENV2 challenge, whereas none of these effects were observed for the NS3 protease domain [389]. When used in combination with a DENV2 purified inactivated vaccine, the NS3 helicase domain but not the NS3 protease domain effectively induced IFN-γ–producing CD4^+^ T cells and markedly increased neutralising antibody titres in mice [390]. In another study, mice that were immunised with recombinant NS3 showed reduced viraemia and shorter bleeding times following DENV2 infection, which was associated with NS3-specific cytotoxic activities and CD107a expression on CD8^+^ T cells [391]. For NS1-based vaccine candidates, it was observed that the depletion of CD4^+^ T cells, and to a lesser extent that of CD8^+^ T cells, significantly impacted vaccine-mediated protection against lethal viral challenge in mice, suggesting a cooperative effect of cellular and humoral immunity [283,392,393]. Others showed that immunisation with recombinant DENV2 NS5 induced a multi-functional T-cell response in mice and provided at least partial protection against a subsequent DENV2 challenge [394]. More recently, researchers have constructed a poly-epitope mRNA vaccine incorporating highly conserved, immunodominant CD8^+^ T-cell epitopes derived from NS3, NS4B, and NS5 of DENV1, and tested its immunogenicity and protective efficacy in mice transgenic for HLA class I molecules associated with either low-magnitude (HLA-A*02:01 and HLA-A*24:02) or high-magnitude T-cell responses (HLA-B*07:02 and HLA-B*35:01) [395]. Irrespective of the HLA background, this vaccine candidate induced a strong T-cell response, which in some cases was cross-reactive for heterologous peptides, and conferred significant protection against infection with DENV1, as defined by the resolution of viraemia [395]. The extent to which such vaccine candidates afford cross-protection against heterologous challenges, however, is largely unknown. One study at least showed that vaccination of HLA-B*07:02–transgenic mice with heterologous CD8^+^ T-cell epitopes prior to a DENV2 challenge could enhance viral clearance [396]. Collectively, these studies highlight the benefit of including T cell–inducing components in dengue vaccine formulations.

## 5. Conclusions and Outlook

Currently, there remains an urgent need for dengue vaccines that can induce robust protective immunity to all four serotypes, irrespective of DENV serostatus at the time of vaccination. In order to develop such vaccines, solid understanding of adaptive immunity to DENV is pivotal. Here, we have reviewed the pathogenic and protective roles of neutralising antibodies, NS1-specific antibodies, and T cells induced by natural infection or vaccination.

Structural and functional studies have demonstrated that the sensitivity of DENV to antibody-mediated neutralisation is modulated by the degree of prM protein cleavage as well as the ensemble of conformational states sampled by virions, which has important implications for vaccine design. Antibodies recognising epitopes that are infrequently exposed on virions, such as the FLE, are more likely to enhance rather than neutralise infection and, therefore, their elicitation by vaccines should be avoided. Instead, vaccine-induced antibody responses should be directed to antigenic sites that are accessible in all known configurations of DENV, i.e., EDIII and the EDE, thereby allowing effective neutralisation at various stages of infection. Strategies to preferentially induce neutralising antibody responses to these epitopes are subject to further investigation. In addition, more relevant assays for the evaluation of vaccine performance need to be developed. The CYD-TDV trials have shown that the ability of vaccine-induced antibodies to neutralise cell culture–derived virions only poorly correlated with their in vivo efficacy [82]. Virus neutralisation tests should therefore include fully mature virions which better represent the structural properties of virions circulating in humans [131].

Recent findings concerning the involvement of NS1 in dengue pathogenesis and its ability to induce protective immune responses have sparked renewed interest in using this protein as a vaccine candidate—partly because it is not included in the currently licenced dengue vaccine. Moreover, the presence of several conserved epitopes on NS1 [274] as well as the cross-inhibitory effects of NS1 antisera observed in animal models [262,297] suggest that a single immunogen may afford protection against multiple serotypes. The main hurdle in developing NS1-based vaccines is, however, the entailed risk of triggering vascular pathology through administration of NS1 by itself or the subsequent induction of autoreactive antibodies. Future studies therefore need to address whether site-specific mutations are required to prevent potentially harmful effects of NS1 vaccination. At the same time, a more detailed analysis of the natural antibody response to NS1 may help identify novel vaccine targets, like the conserved disordered loop of the NS1 WD.

Although virus-specific T cells have been historically implied to contribute to dengue pathogenesis, it has become evident during the last decade that these cells play an important protective role during DENV infections, based on novel insights from murine and human studies. The identification of a large panel of DENV-derived T-cell epitopes presented by common HLA variants has enabled profiling of T-cell responses in naturally infected individuals and vaccinees. This has revealed an intriguing relationship between response magnitude, HLA status, and disease severity [35,343] that should be taken into account when designing new vaccines. Moreover, specific phenotypes of CD4^+^ and CD8^+^ T cells have been observed in individuals carrying HLA alleles associated with reduced disease susceptibility [373,374,375] which might serve as potential correlates of protection. In addition, detailed analyses of vaccine-induced T-cell responses have indicated that DENV-specific CD4^+^ and CD8^+^ T cells may be essential for protective immunity, providing another explanation for the poor performance of CYD-TDV. Finally, the use of individual DENV proteins or immunodominant epitopes as vaccine antigens should be investigated further.

**Figure 10:**
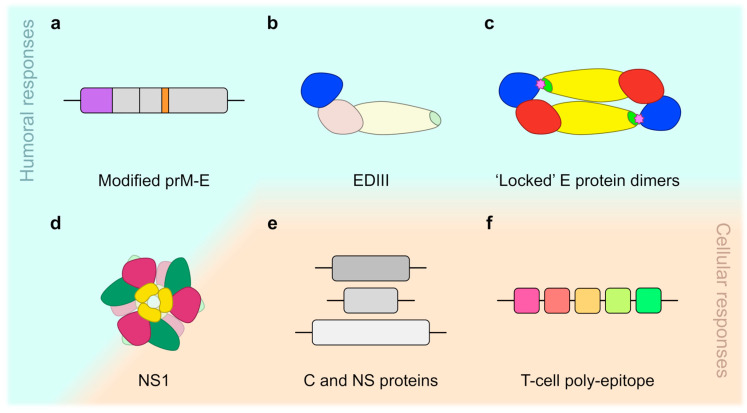
Next-generation dengue vaccines. (**a**) Nucleic acid–based vaccines encoding the prM and E genes—known to generate sub-viral particles [397]—that are modified by replacement of the pr peptide with that of a distantly related flavivirus (e.g., Japanese encephalitis virus (JEV)) (highlighted in purple) [180] and by introduction of mutations in or near the FL (highlighted in orange) [158] should reduce the induction of enhancing prM protein–specific and fusion loop epitope (FLE)-specific antibodies, respectively. (**b**) The proposed role of EDIII in receptor binding as well as its many serotype-specific determinants make it an attractive immunogen for the induction of neutralising antibodies in a tetravalent subunit vaccine approach. (**c**) Covalently locked E protein dimers that display the EDE but hide the FLE can be engineered by introducing inter-subunit disulphide bonds (shown as pink stars) at each end of the dimer [245]. These immunogens might generate protective antibody responses to all four serotypes—but focussing the response on the conserved EDE may require heterologous prime–boost strategies. (**d**) NS1 is a versatile vaccine candidate in that it elicits both cross-protective antibodies—without the risk of antibody-dependent enhancement (ADE)—and T cells. The protein may be modified by site-specific mutations to remove molecular determinants of NS1-mediated endothelial hyperpermeability [266] or to eliminate epitopes that induce antibodies cross-reactive with endothelial cell proteins or coagulation factors [319]. (**e**) DENV proteins that are known to be major targets of T cells should be included in future vaccine formulations to enhance antibody responses and promote viral clearance. (**f**) A more focussed vaccine approach, as compared to **e**, would include a set of well-defined immunodominant CD4^+^ and CD8^+^ T-cell epitopes that induce strong responses in recipients independent of their HLA background [395].

While the other two tetravalent live-attenuated dengue vaccine candidates, TAK-003 and TV003/TV005, are undergoing clinical evaluation, alternative vaccine strategies should be further explored (Figure 10). With these novel vaccines, it may be possible to achieve robust and balanced immunity to all four DENV serotypes.

## References

[1] Brady O.J., Gething P.W., Bhatt S., Messina J.P., Brownstein J.S., Hoen A.G., Moyes C.L., Farlow A.W., Scott T.W., Hay S.I. (2012). Refining the global spatial limits of dengue virus transmission by evidence-based consensus. PLoS Negl. Trop. Dis..

[2] Bhatt S., Gething P.W., Brady O.J., Messina J.P., Farlow A.W., Moyes C.L., Drake J.M., Brownstein J.S., Hoen A.G., Sankoh O. (2013). The global distribution and burden of dengue. Nature.

[3] Chen R., Vasilakis N. (2011). Dengue–Quo tu et quo vadis?. Viruses.

[4] Kraemer M.U., Sinka M.E., Duda K.A., Mylne A.Q., Shearer F.M., Barker C.M., Moore C.G., Carvalho R.G., Coelho G.E., Van Bortel W. (2015). The global distribution of the arbovirus vectors. Aedes Aegypti Ae. Albopictus. Elife.

[5] Simmons C.P., Farrar J.J., van Vinh Chau N., Wills B. (2012). Dengue. N. Engl. J. Med..

[6] Gossner C.M., Ducheyne E., Schaffner F. (2018). Increased risk for autochthonous vector-borne infections transmitted by *Aedes albopictus* in continental Europe. Eurosurveillance.

[7] Guzman M.G., Gubler D.J., Izquierdo A., Martinez E., Halstead S.B. (2016). Dengue infection. Nat. Rev. Dis. Prim..

[8] Martina B.E.E., Koraka P., Osterhaus A.D.M.E. (2009). Dengue virus pathogenesis: An integrated view. Clin. Microbiol. Rev..

[9] Stanaway J.D., Shepard D.S., Undurraga E.A., Halasa Y.A., Coffeng L.E., Brady O.J., Hay S.I., Bedi N., Bensenor I.M., Castañeda-Orjuela C.A. (2016). The global burden of dengue: An analysis from the Global Burden of Disease Study 2013. Lancet Infect. Dis..

[10] Sabin A.B. (1952). Research on dengue during World War II. Am. J. Trop. Med. Hyg..

[11] Imrie A., Meeks J., Gurary A., Sukhbaatar M., Truong T.T., Cropp C.B., Effler P. (2007). Antibody to dengue 1 detected more than 60 years after infection. Viral Immunol..

[12] Waggoner J.J., Balmaseda A., Gresh L., Sahoo M.K., Montoya M., Wang C., Abeynayake J., Kuan G., Pinsky B.A., Harris E. (2016). Homotypic dengue virus reinfections in Nicaraguan children. J. Infect. Dis..

[13] Forshey B.M., Reiner R.C., Olkowski S., Morrison A.C., Espinoza A., Long K.C., Vilcarromero S., Casanova W., Wearing H.J., Halsey E.S. (2016). Incomplete protection against dengue virus type 2 re-infection in Peru. PLoS Negl. Trop. Dis..

[14] Montoya M., Gresh L., Mercado J.C., Williams K.L., Vargas M.J. (2013). Symptomatic versus inapparent outcome in repeat dengue virus infections is influenced by the time interval between infections and study year. PLoS Negl. Trop. Dis..

[15] Anderson K.B., Gibbons R.V., Cummings D.A.T., Nisalak A., Green S., Libraty D.H., Jarman R.G., Srikiatkhachorn A., Mammen M.P., Darunee B. (2014). A shorter time interval between first and second dengue infections is associated with protection from clinical illness in a school-based cohort in Thailand. J. Infect. Dis..

[16] Sangkawibha N., Rojanasuphot S., Ahandrik S., Viriyapongse S., Salttul V., Phanthumachinda B., Halstead S.B., Ro S., Ahandrlk S., Vlrlyapongso S. (1984). Risk factors in dengue shock syndrome: A prospective epldemlologlc study in Rayong, Thailand. I. The 1980 outbreak. Am. J. Epidemiol..

[17] Vaughn D.W., Green S., Kalayanarooj S., Innis B.L., Nimmannitya S., Suntayakorn S., Rothman A.L., Ennis F.A., Nisalak A. (1997). Dengue in the early febrile phase: Viremia and antibody responses. J. Infect. Dis..

[18] Vaughn D.W., Green S., Kalayanarooj S., Innis B.L., Nimmannitya S., Suntayakorn S., Endy T.P., Raengsakulrach B., Rothman A.L., Ennis F.A. (2000). Dengue viremia titer, antibody response pattern, and virus serotype correlate with disease severity. J. Infect. Dis..

[19] OhAinle M., Balmaseda A., Macalalad A.R., Tellez Y., Zody M.C., Saborío S., Nuñez A., Lennon N.J., Birren B.W., Gordon A. (2011). Dynamics of dengue disease severity determined by the interplay between viral genetics and serotype-specific immunity. Sci. Transl. Med..

[20] Halstead S.B., O’Rourke E.J. (1977). Antibody-enhanced dengue virus infection in primate leukocytes. Nature.

[21] Halstead S.B. (1979). In vivo enhancement of dengue virus infection in rhesus monkeys by passively transferred antibody. J. Infect. Dis..

[22] Morens D.M., Venkateshan C.N., Halstead S.B. (1987). Dengue 4 virus monoclonal antibodies identify epitopes that mediate immune infection enhancement of dengue 2 viruses. J. Gen. Virol..

[23] Littaua R., Kurane I., Ennis F.A. (1990). Human IgG Fc receptor II mediates antibody-dependent enhancement of dengue virus infection. J. Immunol..

[24] Goncalvez A.P., Engle R.E., Claire M.S., Purcell R.H., Lai C.-J. (2007). Monoclonal antibody-mediated enhancement of dengue virus infection in vitro and in vivo and strategies for prevention. Proc. Natl. Acad. Sci. USA.

[25] Balsitis S.J., Williams K.L., Lachica R., Flores D., Kyle J.L., Mehlhop E., Johnson S., Diamond M.S., Beatty P.R., Harris E. (2010). Lethal antibody enhancement of dengue disease in mice is prevented by Fc modification. PLoS Pathog..

[26] Zellweger R.M., Prestwood T.R., Shresta S. (2010). Enhanced infection of liver sinusoidal endothelial cells in a mouse model of antibody-induced severe dengue disease. Cell Host Microbe.

[27] Williams K.L., Sukupolvi-Petty S., Beltramello M., Johnson S., Sallusto F., Lanzavecchia A., Diamond M.S., Harris E. (2013). Therapeutic efficacy of antibodies lacking FcγR against lethal dengue virus infection is due to neutralizing potency and blocking of enhancing antibodies. PLoS Pathog..

[28] Nimmanitya S., Kliks S.C., Burke D.S., Nisalak A. (1988). Evidence That maternal dengue antibodies are important in the development of dengue hemorrhagic fever in infants. Am. J. Trop. Med. Hyg..

[29] Chau T.N.B., Hieu N.T., Anders K.L., Wolbers M., Lien L.B., Lu H., Minh T., Hien T.T., Hung N.T., Farrar J. (2009). Dengue virus infections and maternal antibody decay in a prospective birth cohort study of Vietnamese Infants Europe PMC Funders Group. J. Infect. Dis..

[30] Katzelnick L.C., Gresh L., Halloran M.E., Mercado J.C., Kuan G., Gordon A., Balmaseda A., Harris E. (2017). Antibody-dependent enhancement of severe dengue disease in humans. Science.

[31] Waggoner J.J., Katzelnick L.C., Burger-Calderon R., Gallini J., Moore R.H., Kuan G., Balmaseda A., Pinsky B.A., Harris E. (2020). Antibody-dependent enhancement of severe disease is mediated by serum viral load in pediatric dengue virus infections. J. Infect. Dis..

[32] Francis T.J. (1960). On the doctrine of original antigenic sin. Proc. Am. Philos. Soc..

[33] Mongkolsapaya J., Dejnirattisai W., Xu X., Vasanawathana S., Tangthawornchaikul N., Chairunsri A., Sawasdivorn S., Duangchinda T., Dong T., Rowland-Jones S. (2003). Original antigenic sin and apoptosis in the pathogenesis of dengue hemorrhagic fever. Nat. Med..

[34] Duangchinda T., Dejnirattisai W., Vasanawathana S., Limpitikul W., Tangthawornchaikul N., Malasit P., Mongkolsapaya J., Screaton G. (2010). Immunodominant T-cell responses to dengue virus NS3 are associated with DHF. Proc. Natl. Acad. Sci. USA.

[35] Weiskopf D., Angelo M.A., De Azeredo E.L., Sidney J., Greenbaum J.A., Fernando A.N., Broadwater A., Kolla R.V., De Silva A.D., De Silva A.M. (2013). Comprehensive analysis of dengue virus-specific responses supports an HLA-linked protective role for CD8+ T cells. Proc. Natl. Acad. Sci. USA.

[36] Kuhn R.J., Zhang W., Rossmann M.G., Pletnev S.V., Corver J., Lenches E., Jones C.T., Mukhopadhyay S., Chipman P.R., Strauss E.G. (2002). Structure of dengue virus: Implications for flavivirus organization, maturation, and fusion. Cell.

[37] Zhang X., Ge P., Yu X., Brannan J.M., Bi G., Zhang Q., Schein S., Zhou Z.H. (2013). Cryo-EM structure of the mature dengue virus at 3.5-Å resolution. Nat. Struct. Mol. Biol..

[38] Kostyuchenko V.A., Zhang Q., Tan J.L., Ng T.-S., Lok S.-M. (2013). Immature and mature dengue serotype 1 virus structures provide insight into the maturation process. J. Virol..

[39] Kostyuchenko V.A., Chew P.L., Ng T.-S., Lok S.-M. (2014). Near-atomic resolution cryo-electron microscopic structure of dengue serotype 4 virus. J. Virol..

[40] Zhang W., Chipman P.R., Corver J., Johnson P.R., Zhang Y., Mukhopadhyay S., Baker T.S., Strauss J.H., Rossmann M.G., Kuhn R.J. (2003). Visualization of membrane protein domains by cryo-electron microscopy of dengue virus. Nat. Struct. Biol..

[41] Modis Y., Ogata S., Clements D., Harrison S.C. (2003). A ligand-binding pocket in the dengue virus envelope glycoprotein. Proc. Natl. Acad. Sci. USA..

[42] Modis Y., Ogata S., Clements D., Harrison S.C. (2005). Variable surface epitopes in the crystal structure of dengue virus type 3 envelope glycoprotein. J. Virol..

[43] Zhang Y., Zhang W., Ogata S., Clements D., Strauss J.H., Baker T.S., Kuhn R.J., Rossmann M.G. (2004). Conformational changes of the flavivirus E glycoprotein. Structure.

[44] Chen Y., Maguire T., Hileman R.E., Fromm J.R., Esko J.D., Linhardt R.J., Marks R.M. (1997). Dengue virus infectivity depends on envelope protein binding to target cell heparan sulfate. Nat. Med..

[45] Crill W.D., Roehrig J.T. (2001). Monoclonal antibodies that bind to domain III of dengue virus E glycoprotein are the most efficient blockers of virus adsorption to Vero cells. J. Virol..

[46] Hung J.-J., Hsieh M.-T., Young M.-J., Kao C.-L., King C.-C., Chang W. (2004). An external loop region of domain III of dengue virus type 2 envelope protein is involved in serotype-specific binding to mosquito but not mammalian cells. J. Virol..

[47] Chin J.F.L., Chu J.J.H., Ng M.L. (2007). The envelope glycoprotein domain III of dengue virus serotypes 1 and 2 inhibit virus entry. Microbes Infect..

[48] Watterson D., Kobe B., Young P.R. (2012). Residues in domain III of the dengue virus envelope glycoprotein involved in cell-surface glycosaminoglycan binding. J. Gen. Virol..

[49] Pokidysheva E., Zhang Y., Battisti A.J., Bator-Kelly C.M., Chipman P.R., Xiao C., Gregorio G.G., Hendrickson W.A., Kuhn R.J., Rossmann M.G. (2006). Cryo-EM reconstruction of dengue virus in complex with the carbohydrate recognition domain of DC-SIGN. Cell.

[50] Miller J.L., de Wet B.J.M., deWet B.J.M., Martinez-Pomares L., Radcliffe C.M., Dwek R.A., Rudd P.M., Gordon S. (2008). The mannose receptor mediates dengue virus infection of macrophages. PLoS Pathog..

[51] Navarro-Sanchez E., Altmeyer R., Amara A., Schwartz O., Fieschi F., Virelizier J.-L., Arenzana-Seisdedos F., Desprès P. (2003). Dendritic-cell-specific ICAM3-grabbing non-integrin is essential for the productive infection of human dendritic cells by mosquito-cell-derived dengue viruses. EMBO Rep..

[52] Tassaneetrithep B., Burgess T.H., Granelli-Piperno A., Trumpfheller C., Finke J., Sun W., Eller M.A., Pattanapanyasat K., Sarasombath S., Birx D.L. (2003). DC-SIGN (CD209) mediates dengue virus infection of human dendritic cells. J. Exp. Med..

[53] Meertens L., Carnec X., Lecoin M.P., Ramdasi R., Guivel-Benhassine F., Lew E., Lemke G., Schwartz O., Amara A. (2012). The TIM and TAM families of phosphatidylserine receptors mediate dengue virus entry. Cell Host Microbe.

[54] van der Schaar H.M., Rust M.J., Chen C., van der Ende-Metselaar H., Wilschut J., Zhuang X., Smit J.M. (2008). Dissecting the cell entry pathway of dengue virus by single-particle tracking in living cells. PLoS Pathog..

[55] Bressanelli S., Stiasny K., Allison S.L., Stura E.A., Duquerroy S., Lescar J., Heinz F.X., Rey F.A. (2004). Structure of a flavivirus envelope glycoprotein in its low-pH-induced membrane fusion conformation. EMBO J..

[56] Modis Y., Ogata S., Clements D., Harrison S.C. (2004). Structure of the dengue virus envelope protein after membrane fusion. Nature.

[57] Nour A.M., Li Y., Wolenski J., Modis Y. (2013). Viral membrane fusion and nucleocapsid delivery into the cytoplasm are distinct events in some flaviviruses. PLoS Pathog..

[58] Mackenzie J.M., Westaway E.G., Sakzewski A. (2001). Assembly and maturation of the flavivirus kunjin virus appear to occur in the rough endoplasmic reticulum and along the secretory pathway, respectively. J. Virol..

[59] Welsch S., Miller S., Romero-Brey I., Merz A., Bleck C.K.E., Walther P., Fuller S.D., Antony C., Krijnse-Locker J., Bartenschlager R. (2009). Composition and three-dimensional architecture of the dengue virus replication and assembly sites. Cell Host Microbe.

[60] Zhang Y., Corver J., Chipman P.R., Zhang W., Pletnev S.V., Sedlak D., Baker T.S., Strauss J.H., Kuhn R.J., Rossmann M.G. (2003). Structures of immature flavivirus particles. EMBO J..

[61] Li L., Lok S.-M., Yu I.-M., Zhang Y., Kuhn R.J., Chen J., Rossmann M.G. (2008). The flavivirus precursor membrane-envelope protein complex: Structure and maturation. Science.

[62] Stadler K., Allison S.L., Schalich J., Heinz F.X. (1997). Proteolytic activation of tick-borne encephalitis virus by furin. J. Virol..

[63] Yu I.-M., Zhang W., Holdaway H.A., Li L., Kostyuchenko V.A., Chipman P.R., Kuhn R.J., Rossmann M.G., Chen J. (2008). Structure of the immature dengue virus at low pH primes proteolytic maturation. Science.

[64] Guirakhoo F., Heinz F.X., Mandl C.W., Holzmann H., Kunz C. (1991). Fusion activity of flaviviruses: Comparison of mature and immature (prM-containing) tick-borne encephalitis virions. J. Gen. Virol..

[65] Yu I.-M., Holdaway H.A., Chipman P.R., Kuhn R.J., Rossmann M.G., Chen J. (2009). Association of the pr peptides with dengue virus at acidic pH blocks membrane fusion. J. Virol..

[66] Junjhon J., Lausumpao M., Supasa S., Noisakran S., Songjaeng A., Saraithong P., Chaichoun K., Utaipat U., Keelapang P., Kanjanahaluethai A. (2008). Differential modulation of prM cleavage, extracellular particle distribution, and virus infectivity by conserved residues at nonfurin consensus positions of the dengue virus pr-M junction. J. Virol..

[67] Zybert I.A., van der Ende-Metselaar H., Wilschut J., Smit J.M. (2008). Functional importance of dengue virus maturation: Infectious properties of immature virions. J. Gen. Virol..

[68] Cherrier M.V., Kaufmann B., Nybakken G.E., Lok S.-M., Warren J.T., Chen B.R., Nelson C.A., Kostyuchenko V.A., Holdaway H.A., Chipman P.R. (2009). Structural basis for the preferential recognition of immature flaviviruses by a fusion-loop antibody. EMBO J..

[69] Junjhon J., Edwards T.J., Utaipat U., Bowman V.D., Holdaway H.A., Zhang W., Keelapang P., Puttikhunt C., Perera R., Chipman P.R. (2010). Influence of pr-M Cleavage on the Heterogeneity of Extracellular Dengue Virus Particles. J. Virol..

[70] Dejnirattisai W., Jumnainsong A., Onsirisakul N., Fitton P., Vasanawathana S., Limpitikul W., Puttikhunt C., Edwards C., Duangchinda T., Supasa S. (2010). Cross-reacting antibodies enhance dengue virus infection in humans. Science.

[71] Dejnirattisai W., Wongwiwat W., Supasa S., Zhang X., Dai X., Rouvinski A., Jumnainsong A., Edwards C., Quyen N.T.H., Duangchinda T. (2015). A new class of highly potent, broadly neutralizing antibodies isolated from viremic patients infected with dengue virus. Nat. Immunol..

[72] Randolph V.B., Winkler G., Stollar V. (1990). Acidotropic amines inhibit proteolytic processing of flavivirus prM protein. Virology.

[73] Plevka P., Battisti A.J., Junjhon J., Winkler D.C., Holdaway H.A., Keelapang P., Sittisombut N., Kuhn R.J., Steven A.C., Rossmann M.G. (2011). Maturation of flaviviruses starts from one or more icosahedrally independent nucleation centres. EMBO Rep..

[74] Tomasello G., Armenia I., Molla G. (2020). The Protein Imager: A full-featured online molecular viewer interface with server-side HQ-rendering capabilities. Bioinformatics.

[75] Pettersen E.F., Goddard T.D., Huang C.C., Couch G.S., Greenblatt D.M., Meng E.C., Ferrin T.E. (2004). UCSF Chimera—A visualization system for exploratory research and analysis. J. Comput. Chem..

[76] Guirakhoo F., Arroyo J., Pugachev K.V., Miller C., Zhang Z.X., Weltzin R., Georgakopoulos K., Catalan J., Ocran S., Soike K. (2001). Construction, safety, and immunogenicity in nonhuman primates of a chimeric yellow fever-dengue virus tetravalent vaccine. J. Virol..

[77] Morrison D., Legg T.J., Billings C.W., Forrat R., Yoksan S., Lang J. (2010). A novel tetravalent dengue vaccine is well tolerated and immunogenic against all 4 serotypes in flavivirus -naive adults. J. Infect. Dis..

[78] Capeding R.Z., Luna I.A., Bomasang E., Lupisan S., Lang J., Forrat R., Wartel A., Crevat D. (2011). Live-attenuated, tetravalent dengue vaccine in children, adolescents and adults in a dengue endemic country: Randomized controlled phase I trial in the Philippines. Vaccine.

[79] Sabchareon A., Wallace D., Sirivichayakul C., Limkittikul K., Chanthavanich P., Suvannadabba S., Jiwariyavej V., Dulyachai W., Pengsaa K., Wartel T.A. (2012). Protective efficacy of the recombinant, live-attenuated, CYD tetravalent dengue vaccine in Thai schoolchildren: A randomised, controlled phase 2b trial. Lancet.

[80] Capeding M.R., Tran N.H., Hadinegoro S.R.S., Ismail H.I.H.M., Chotpitayasunondh T., Chua M.N., Luong C.Q., Rusmil K., Wirawan D.N., Nallusamy R. (2014). Clinical efficacy and safety of a novel tetravalent dengue vaccine in healthy children in Asia: A phase 3, randomised, observer-masked, placebo-controlled trial. Lancet.

[81] Villar L., Dayan G.H., Arredondo-García J.L., Rivera D.M., Cunha R., Deseda C., Reynales H., Costa M.S., Morales-Ramírez J.O., Carrasquilla G. (2015). Efficacy of a tetravalent dengue vaccine in children in Latin America. N. Engl. J. Med..

[82] Hadinegoro S.R., Arredondo-García J.L., Capeding M.R., Deseda C., Chotpitayasunondh T., Dietze R., Ismail H.I.H.M., Reynales H., Limkittikul K., Rivera-Medina D.M. (2015). Efficacy and long-term safety of a dengue vaccine in regions of endemic disease. N. Engl. J. Med..

[83] Guy B., Jackson N. (2016). Dengue vaccine: Hypotheses to understand CYD-TDV-induced protection. Nat. Rev. Microbiol..

[84] Halstead S.B. (2017). Dengvaxia sensitizes seronegatives to vaccine enhanced disease regardless of age. Vaccine.

[85] Sridhar S., Luedtke A., Langevin E., Zhu M., Bonaparte M., Machabert T., Savarino S., Zambrano B., Moureau A., Khromava A. (2018). Effect of dengue serostatus on dengue vaccine safety and efficacy. N. Engl. J. Med..

[86] Wilder-Smith A., Flasche S., Smith P.G. (2019). Vaccine-attributable severe dengue in the Philippines. Lancet.

[87] Wilder-Smith A., Hombach J., Ferguson N., Selgelid M., Brien K.O., Vannice K., Barrett A., Ferdinand E., Flasche S., Guzman M. (2019). Deliberations of the strategic advisory group of experts on immunization on the use of CYD-TDV dengue vaccine. Lancet Infect. Dis..

[88] Butrapet S., Huang C.Y., Pierro D.J., Bhamarapravati N., Gubler D.J., Kinney R.M. (2000). Attenuation markers of a candidate dengue type 2 vaccine virus, strain 16681 (PDK-53), are defined by mutations in the 5′ noncoding region and nonstructural proteins 1 and 3. J. Virol..

[89] Huang C.Y.-H., Butrapet S., Tsuchiya K.R., Bhamarapravati N., Gubler D.J., Kinney R.M. (2003). Dengue 2 PDK-53 virus as a chimeric carrier for tetravalent dengue vaccine development. J. Virol..

[90] Osorio J.E., Velez I.D., Thomson C., Lopez L., Jimenez A., Haller A.A., Silengo S., Scott J., Boroughs K.L., Stovall J.L. (2014). Safety and immunogenicity of a recombinant live attenuated tetravalent dengue vaccine (DENVax) in flavivirus-naive healthy adults in Colombia: A randomised, placebo-controlled, phase 1 study. Lancet Infect. Dis..

[91] Sirivichayakul C., Barranco-Santana E.A., Esquilin-Rivera I., Oh H.M.L., Raanan M., Sariol C.A., Shek L.P., Simasathien S., Smith M.K., Velez I.D. (2016). Safety and immunogenicity of a tetravalent dengue vaccine candidate in healthy children and adults in dengue-endemic regions: A randomized, placebo-controlled phase 2 study. J. Infect. Dis..

[92] Biswal S., Reynales H., Saez-Llorens X., Lopez P., Borja-Tabora C., Kosalaraksa P., Sirivichayakul C., Watanaveeradej V., Rivera L., Espinoza F. (2019). Efficacy of a tetravalent dengue vaccine in healthy children and adolescents. N. Engl. J. Med..

[93] Biswal S., Borja-Tabora C., Vargas L.M., Velásquez H., Alera M.T., Sierra V., Rodriguez-Arenales E.J., Yu D., Wickramasinghe V.P., Moreira E.D. (2020). Efficacy of a tetravalent dengue vaccine in healthy children aged 4–16 years: A randomised, placebo-controlled, phase 3 trial. Lancet.

[94] Blaney J.E., Durbin A.P., Murphy B.R., Whitehead S.S. (2006). Development of a live attenuated dengue virus vaccine using reverse genetics. Viral Immunol..

[95] Durbin A.P., Mcarthur J.H., Marron J.A., Blaney J.E., Thumar B., Wanionek K., Murphy B.R., Whitehead S.S. (2006). Chimeric dengue serotype 2 vaccine, is safe and highly immunogenic in healthy dengue-naïve adults. Hum. Vaccin..

[96] Durbin A.P., Kirkpatrick B.D., Pierce K.K., Elwood D., Larsson C.J., Lindow J.C., Tibery C., Sabundayo B.P., Shaffer D., Talaat K.R. (2013). A single dose of any of four different live attenuated tetravalent dengue vaccines is safe and immunogenic in flavivirus-naive adults: A randomized, double-blind clinical trial. J. Infect. Dis..

[97] Kirkpatrick B.D., Durbin A.P., Pierce K.K., Carmolli M.P., Tibery C.M., Grier P.L., Hynes N., Diehl S.A., Elwood D., Jarvis A.P. (2015). Robust and balanced immune responses to all 4 dengue virus serotypes following administration of a single dose of a live attenuated tetravalent dengue vaccine to healthy, flavivirus-naive adults. J. Infect. Dis..

[98] Whitehead S.S., Durbin A.P., Pierce K.K., Elwood D., McElvany B.D., Fraser E.A., Carmolli M.P., Tibery C.M., Hynes N.A., Jo M. (2017). In a randomized trial, the live attenuated tetravalent dengue vaccine TV003 is well-tolerated and highly immunogenic in subjects with flavivirus exposure prior to vaccination. PLoS Negl. Trop. Dis..

[99] Kirkpatrick B.D., Whitehead S.S., Pierce K.K., Tibery C.M., Grier P.L., Hynes N.A., Larsson C.J., Sabundayo B.P., Talaat K.R., Janiak A. (2016). The live attenuated dengue vaccine TV003 elicits complete protection against dengue in a human challenge model. Sci. Transl. Med..

[100] Halstead S.B., Russell P.K. (2016). Protective and immunological behavior of chimeric yellow fever dengue vaccine. Vaccine.

[101] Henein S., Swanstrom J., Byers A.M., Moser J.M., Shaik S.F., Bonaparte M., Jackson N., Guy B., Baric R., de Silva A.M. (2016). Dissecting antibodies induced by a chimeric yellow fever-dengue, live-attenuated, tetravalent dengue vaccine (CYD-TDV) in naïve and dengue exposed individuals. J. Infect. Dis..

[102] Schlesinger J.J., Brandriss M.W., Walsh E.E. (1987). Protection of mice against dengue 2 virus encephalitis by immunization with the dengue 2 virus non-structural glycoprotein NS1. J. Gen. Virol..

[103] Yauch L.E., Zellweger R.M., Kotturi M.F., Qutubuddin A., Sidney J., Peters B., Prestwood T.R., Sette A., Shresta S. (2009). A protective role for dengue virus-specific CD8 + T Cells. J. Immunol..

[104] Lai C.-Y., Tsai W.-Y., Lin S.-R., Kao C.-L., Hu H.-P., King C.-C., Wu H.-C., Chang G.-J., Wang W.-K. (2008). Antibodies to envelope glycoprotein of dengue virus during the natural course of infection are predominantly cross-reactive and recognize epitopes containing highly conserved residues at the fusion loop of domain II. J. Virol..

[105] Beltramello M., Williams K.L., Simmons C.P., Macagno A., Simonelli L., Than N., Quyen H., Sukupolvi-Petty S., Navarro-Sanchez E., Young P.R. (2010). The human immune response to dengue virus is dominated by highly cross-reactive antibodies endowed with neutralizing and enhancing activity. Cell Host Microbe.

[106] de Alwis R., Beltramello M., Messer W.B., Sukupolvi-Petty S., Wahala W.M.P.B., Kraus A., Olivarez N.P., Pham Q., Brian J., Tsai W.-Y. (2011). In-depth analysis of the antibody response of individuals exposed to primary dengue virus infection. PLoS Negl. Trop. Dis..

[107] de Alwis R., Smith S.A., Olivarez N.P., Messer W.B., Huynh J.P., Wahala W.M.P.B., White L.J., Diamond M.S., Baric R.S., Crowe J.E. (2012). Identification of human neutralizing antibodies that bind to complex epitopes on dengue virions. Proc. Natl. Acad. Sci. USA.

[108] Smith S.A., Zhou Y., Olivarez N.P., Broadwater A.H., de Silva A.M., Crowe J.E. (2012). Persistence of circulating memory B cell clones with potential for dengue virus disease enhancement for decades following infection. J. Virol..

[109] Smith S.A., de Alwis A.R., Kose N., Harris E., Ibarra K.D., Kahle K.M., Pfaff J.M., Xiang X., Doranz B.J., de Silva A.M. (2013). The potent and broadly neutralizing human dengue virus-specific monoclonal antibody 1C19 reveals a unique cross-reactive epitope on the bc loop of domain II of the envelope protein. mBio.

[110] Smith S.A., de Alwis A.R., Kose N., Jadi R.S., de Silva A.M., Crowe J.E. (2014). Isolation of dengue virus-specific memory B cells with live virus antigen from human subjects following natural infection reveals the presence of diverse novel functional groups of antibody clones. J. Virol..

[111] Appanna R., KG S., Xu M.H., Toh Y.-X., Velumani S., Carbajo D., Lee C.Y., Zuest R., Balakrishnan T., Xu W. (2016). Plasmablasts during acute dengue infection represent a small subset of a broader virus-specific memory B cell pool. EBioMedicine.

[112] Priyamvada L., Cho A., Onlamoon N., Zheng N.-Y., Huang M., Kovalenkov Y., Chokephaibulkit K., Angkasekwinai N., Pattanapanyasat K., Ahmed R. (2016). B cell responses during secondary dengue virus infection are dominated by highly cross-reactive, memory-derived plasmablasts. J. Virol..

[113] Patel B., Longo P., Miley M.J., Montoya M., Harris E., de Silva A.M. (2017). Dissecting the human serum antibody response to secondary dengue virus infections. PLoS Negl. Trop. Dis..

[114] Nivarthi U.K., Tu H.A., Delacruz M.J., Swanstrom J., Patel B., Durbin A.P., Whitehead S.S., Pierce K.K., Kirkpatrick B.D., Baric R.S. (2019). Longitudinal analysis of acute and convalescent B cell responses in a human primary dengue serotype 2 infection model. EBioMedicine.

[115] Andrade P., Narvekar P., Montoya M., Michlmayr D., Balmaseda A., Coloma J., Harris E. (2020). Primary and secondary dengue virus infections elicit similar memory B cell responses but breadth to other serotypes and cross-reactivity to Zika virus is higher in secondary dengue. J. Infect. Dis..

[116] Chan K.R., Zhang S.L.-X., Tan H.C., Chan Y.K., Chow A., Lim A.P.C., Vasudevan S.G., Hanson B.J., Ooi E.E. (2011). Ligation of Fc gamma receptor IIB inhibits antibody-dependent enhancement of dengue virus infection. Proc. Natl. Acad. Sci. USA.

[117] Mathew A., West K., Kalayanarooj S., Gibbons R.V., Srikiatkhachorn A., Green S., Libraty D., Jaiswal S., Rothman A.L. (2011). B-cell responses during primary and secondary dengue virus infections in humans. J. Infect. Dis..

[118] Zompi S., Montoya M., Pohl M.O., Balmaseda A., Harris E. (2012). Dominant cross-reactive B cell response during secondary acute dengue virus infection in humans. PLoS Negl. Trop. Dis..

[119] Xu M., Hadinoto V., Appanna R., Joensson K., Toh Y.X., Balakrishnan T., Ong S.H., Warter L., Leo Y.S., Wang C.-I. (2012). Plasmablasts generated during repeated dengue infection are virus glycoprotein-specific and bind to multiple virus serotypes. J. Immunol..

[120] Wrammert J., Onlamoon N., Akondy R.S., Perng G.C., Polsrila K., Chandele A., Kwissa M., Pulendran B., Wilson P.C., Wittawatmongkol O. (2012). Rapid and massive virus-specific plasmablast responses during acute dengue virus infection in humans. J. Virol..

[121] Toh Y.X., Gan V., Balakrishnan T., Zuest R., Poidinger M., Wilson S., Appanna R., Thein T.L., Ong A.K.-Y., Ng L.C. (2014). Dengue serotype cross-reactive, anti-E protein antibodies confound specific immune memory for 1 year after infection. Front. Immunol..

[122] Woda M., Friberg H., Currier J.R., Srikiatkhachorn A., Macareo L.R., Green S., Jarman R.G., Rothman A.L., Mathew A. (2016). Dynamics of dengue virus (DENV)–specific B cells in the response to DENV serotype 1 infections, using flow cytometry with labeled virions. J. Infect. Dis..

[123] Xu M., Züst R., Toh Y.X., Pfaff J.M., Kahle K.M., Davidson E., Doranz B.J., Velumani S., Tukijan F., Wang C.-I. (2016). Protective capacity of the human anamnestic antibody response during acute dengue virus infection. J. Virol..

[124] Lai C.-Y., Williams K.L., Wu Y.-C., Knight S., Balmaseda A., Harris E., Wang W.-K. (2013). Analysis of cross-reactive antibodies recognizing the fusion loop of envelope protein and correlation with neutralizing antibody titers in Nicaraguan dengue cases. PLoS Negl. Trop. Dis..

[125] Tsai W.-Y., Lai C.-Y., Wu Y.-C., Lin H.-E., Edwards C., Jumnainsong A., Kliks S., Halstead S., Mongkolsapaya J., Screaton G.R. (2013). High-avidity and potently neutralizing cross-reactive human monoclonal antibodies derived from secondary dengue virus infection. J. Virol..

[126] Tsai W.-Y., Durbin A., Tsai J.-J., Hsieh S.-C., Whitehead S., Wang W.-K. (2015). Complexity of neutralizing antibodies against multiple dengue virus serotypes after heterotypic immunization and secondary infection revealed by in-depth analysis of cross-reactive antibodies. J. Virol..

[127] Gibbons R.V., Kalanarooj S., Jarman R.G., Nisalak A., Vaughn D.W., Endy T.P., Mammen M.P., Srikiatkhachorn A. (2007). Analysis of repeat hospital admissions for dengue to estimate the frequency of third or fourth dengue infections resulting in admissions and dengue hemorrhagic fever, and serotype sequences. Am. J. Trop. Med. Hyg..

[128] Bhoomiboonchoo P., Nisalak A., Chansatiporn N., Yoon I.-K., Kalayanarooj S., Thipayamongkolgul M., Endy T., Rothman A.L., Green S., Srikiatkhachorn A. (2010). Sequential dengue virus infections detected in active and passive surveillance programs in Thailand, 1994–2010. BMC Public Health.

[129] Olkowski S., Forshey B.M., Morrison A.C., Rocha C., Vilcarromero S., Halsey E.S., Kochel T.J., Scott T.W., Stoddard S.T. (2013). Reduced risk of disease during postsecondary dengue virus infections. J. Infect. Dis..

[130] Pierson T.C., Xu Q., Nelson S., Oliphant T., Nybakken G.E., Fremont D.H., Diamond M.S. (2007). The stoichiometry of antibody-mediated neutralization and enhancement of West Nile virus infection. Cell Host Microbe.

[131] Raut R., Corbett K.S., Tennekoon R.N., Premawansa S., Wijewickrama A., Premawansa G., Mieczkowski P., Rückert C., Ebel G.D., De Silva A.D. (2019). Dengue type 1 viruses circulating in humans are highly infectious and poorly neutralized by human antibodies. Proc. Natl. Acad. Sci. USA.

[132] Nelson S., Jost C.A., Xu Q., Ess J., Martin J.E., Oliphant T., Whitehead S.S., Durbin A.P., Graham B.S., Dimaond M.S. (2008). Maturation of West Nile virus modulates sensitivity to antibody-mediated neutralization. PLoS Pathog..

[133] Dowd K.A., Mukherjee S., Kuhn R.J., Pierson T.C. (2014). Combined effects of the structural heterogeneity and dynamics of flaviviruses on antibody recognition. J. Virol..

[134] Fibriansah G., Ng T.-S., Kostyuchenko V.A., Lee J., Lee S., Wang J., Lok S.-M. (2013). Structural changes in dengue virus when exposed to a temperature of 37 °C. J. Virol..

[135] Zhang X., Sheng J., Plevka P., Kuhn R.J., Diamond M.S., Rossmann M.G. (2013). Dengue structure differs at the temperatures of its human and mosquito hosts. Proc. Natl. Acad. Sci. USA.

[136] Lim X.-N., Shan C., Marzinek J.K., Dong H., Ng T.S., Ooi J.S.G., Fibriansah G., Wang J., Verma C.S., Bond P.J. (2019). Molecular basis of dengue virus serotype 2 morphological switch from 29 °C to 37 °C. PLoS Pathog..

[137] Lok S.-M., Kostyuchenko V., Nybakken G.E., Holdaway H.A., Battisti A.J., Sukupolvi-Petty S., Sedlak D., Fremont D.H., Chipman P.R., Roehrig J.T. (2008). Binding of a neutralizing antibody to dengue virus alters the arrangement of surface glycoproteins. Nat. Struct. Mol. Biol..

[138] Dowd K.A., Jost C.A., Durbin A.P., Whitehead S.S., Pierson T.C. (2011). A dynamic landscape for antibody binding modulates antibody-mediated neutralization of West Nile virus. PLoS Pathog..

[139] Austin S.K., Dowd K.A., Shrestha B., Nelson C.A., Edeling M.A., Johnson S., Pierson T.C., Diamond M.S., Fremont D.H. (2012). Structural basis of differential neutralization of DENV-1 genotypes by an antibody that recognizes a cryptic epitope. PLoS Pathog..

[140] Sukupolvi-Petty S., Brien J.D., Austin S.K., Shrestha B., Swayne S., Kahle K., Doranz B.J., Johnson S., Pierson T.C., Fremont D.H. (2013). Functional analysis of antibodies against dengue virus type 4 reveals strain-dependent epitope exposure that impacts neutralization and protection. J. Virol..

[141] Dowd K.A., DeMaso C.R., Pierson T.C. (2015). Genotypic differences in dengue virus neutralization are explained by a single amino acid mutation that modulates virus breathing. mBio.

[142] Crill W.D., Hughes H.R., Delorey M.J., Chang G.-J.J. (2009). Humoral immune responses of dengue fever patients using epitope-specific serotype-2 virus-like particle antigens. PLoS ONE.

[143] Wahala W.M.P.B., Kraus A.A., Haymore L.B., Accavitti-Loper M.A., De Silva A.M. (2009). Dengue virus neutralization by human immune sera: Role of envelope protein domain III-reactive antibody. Virology.

[144] Lin H.-E., Tsai W.-Y., Liu I.-J., Li P.-C., Liao M.-Y., Tsai J.-J., Wu Y.-C., Lai C.-Y., Lu C.-H., Huang J.-H. (2012). Analysis of epitopes on dengue virus envelope protein recognized by monoclonal antibodies and polyclonal human sera by a high throughput assay. PLoS Negl. Trop. Dis..

[145] Smith S.A., de Alwis R., Kose N., Durbin A.P., Whitehead S.S., de Silva A.M., Crowe J.E. (2013). Human monoclonal antibodies derived from memory B cells following live attenuated dengue virus vaccination or natural infection exhibit similar characteristics. J. Infect. Dis..

[146] de Alwis R., Williams K.L., Schmid M.A., Lai C.-Y., Patel B. (2014). Dengue viruses are enhanced by distinct populations of serotype cross-reactive antibodies in human immune sera. PLoS Pathog..

[147] Crill W.D., Chang G.-J.J. (2004). Localization and characterization of flavivirus envelope glycoprotein cross-reactive epitopes. J. Virol..

[148] Oliphant T., Nybakken G.E., Engle M., Xu Q., Nelson C.A., Sukupolvi-Petty S., Marri A., Lachmi B.-E., Olshevsky U., Fremont D.H. (2006). Antibody recognition and neutralization determinants on domains I and II of West Nile Virus envelope protein. J. Virol..

[149] Costin J.M., Zaitseva E., Kahle K.M., Nicholson C.O., Rowe D.K., Graham A.S., Bazzone L.E., Hogancamp G., Sierra M.F., Fong R.H. (2013). Mechanistic study of broadly neutralizing human monoclonal antibodies against dengue virus that target the fusion loop. J. Virol..

[150] Dai L., Song J., Lu X., Qin C.-F., Qi J., Gao G.F. (2016). Structures of the Zika virus envelope protein and its complex with a flavivirus broadly protective antibody. Cell Host Microbe.

[151] Dejnirattisai W., Supasa P., Wongwiwat W., Rouvinski A., Barba-Spaeth G., Duangchinda T., Sakuntabhai A., Cao-Lormeau V.-M., Malasit P., Rey F.A. (2016). Dengue virus sero-cross-reactivity drives antibody-dependent enhancement of infection with zika virus. Nat. Immunol..

[152] Chaudhury S., Gromowski G.D., Ripoll D.R., Khavrutskii I.V., Desai V., Wallqvist A. (2017). Dengue virus antibody database: Systematically linking serotype-specificity with epitope mapping in dengue virus. PLoS Negl. Trop. Dis..

[153] Gentry M.K., Henchal E.A., Mccown J.M., Brandt W.E., Dalrymple J.M. (1982). Identification of distinct antigenic determinants on dengue-2 virus using monoclonal antibodies. Am. J. Trop. Med. Hyg..

[154] Serafin I.L., Aaskov J.G. (2001). Identification of epitopes on the envelope (E) protein of dengue 2 and dengue 3 viruses using monoclonal antibodies. Arch. Virol..

[155] Tsai W.-Y., Chen H.-L., Tsai J.-J., Dejnirattisai W., Jumnainsong A., Mongkolsapaya J., Screaton G., Crowe J.E., Wang W.-K., Wang W.-K. (2018). Potent neutralizing human monoclonal antibodies preferentially target mature dengue virus particles: Implication for novel strategy for dengue vaccine. J. Virol..

[156] Huang C.Y.-H., Butrapet S., Moss K.J., Childers T., Erb S.M., Calvert A.E., Silengo S.J., Kinney R.M., Blair C.D., Roehrig J.T. (2010). The dengue virus type 2 envelope protein fusion peptide is essential for membrane fusion. Virology.

[157] Hughes H.R., Crill W.D., Chang G.-J.J. (2012). Manipulation of immunodominant dengue virus E protein epitopes reduces potential antibody-dependent enhancement. Virol. J..

[158] Crill W.D., Hughes H.R., Trainor N.B., Davis B.S., Whitney M.T., Chang G.-J.J. (2012). Sculpting humoral immunity through dengue vaccination to enhance protective immunity. Front. Immunol..

[159] Richner J.M., Himansu S., Dowd K.A., Pierson T.C., Ciaramella G., Diamond M.S. (2017). Modified mRNA vaccines protect against Zika virus infection. Cell.

[160] Huang K.-J., Yang Y.-C., Lin Y.-S., Huang J.-H., Liu H.-S., Yeh T.-M., Chen S.-H., Liu C.-C., Lei H.-Y. (2006). The dual-specific binding of dengue virus and target cells for the antibody-dependent enhancement of dengue virus infection. J. Immunol..

[161] Luo Y.-Y., Feng J.-J., Zhou J.-M., Yu Z.-Z., Fang D.-Y., Yan H.-J., Zeng G.-C., Jiang L.-F. (2013). Identification of a novel infection-enhancing epitope on dengue prM using a dengue cross-reacting monoclonal antibody. BMC Microbiol..

[162] Wang Z., Li L., Pennington J.G., Sheng J., Yap M.L., Plevka P., Meng G., Sun L., Jiang W., Rossmann M.G. (2013). Obstruction of dengue virus maturation by Fab fragments of the 2H2 antibody. J. Virol..

[163] Smith S.A., Nivarthi U.K., de Alwis R., Kose N., Sapparapu G., Bombardi R., Kahle K.M., Pfaff J.M., Lieberman S., Doranz B.J. (2015). Dengue virus prM-specific human monoclonal antibodies with virus replication-enhancing properties recognize a single immunodominant antigenic site. J. Virol..

[164] Falconar A.K.I. (1999). Identification of an epitope on the dengue virus membrane (M) protein defined by cross-protective monoclonal antibodies: Design of an improved epitope sequence based on common determinants present in both envelope (E and M) proteins. Arch. Virol..

[165] Puttikhunt C., Keelapang P., Khemnu N., Sittisombut N., Kasinrerk W., Malasit P. (2008). Novel anti-dengue monoclonal antibody recognizing conformational structure of the prM-E heterodimeric complex of dengue virus. J. Med. Virol..

[166] Chan A.H.Y., Tan H.C., Chow A.Y., Lim A.P.C., Lok S.M., Moreland N.J., Vasudevan S.G., MacAry P.A., Ooi E.E., Hanson B.J. (2012). A Human PrM antibody that recognizes a novel cryptic epitope on dengue E glycoprotein. PLoS ONE.

[167] Henchal E.A., Mccown J.M., Burke D.S., Seguin M.C., Brandt W.E., Brandt W.E. (1985). Epitopic analysis of antigenic determinants on the surface of dengue-2 virions using monoclonal antibodies. Am. J. Trop. Med. Hyg..

[168] Vázquez S., Guzmán M.G., Guillen G., Chinea G., Pérez A.B., Pupo M., Rodriguez R., Reyes O., Garay H.E., Delgado I. (2002). Immune response to synthetic peptides of dengue prM protein. Vaccine.

[169] Kaufman B.M., Summers P.L., Dubois D.R., Cohen W.H., Gentry M.K., Timchak R.L., Burke D.S., Eckels K.H. (1989). Monoclonal antibodies for dengue virus prM glycoprotein protect mice against lethal dengue infection. Am. J. Trop. Med. Hyg..

[170] Rodenhuis-Zybert I.A., Wilschut J., Smit J.M. (2011). Partial maturation: An immune-evasion strategy of dengue virus?. Trends Microbiol..

[171] Rodenhuis-Zybert I.A., van der Schaar H.M., da Silva Voorham J.M., van der Ende-Metselaar H., Lei H.-Y., Wilschut J., Smit J.M. (2010). Immature dengue virus: A veiled pathogen?. PLoS Pathog..

[172] da Silva Voorham J.M., Rodenhuis-Zybert I.A., Nuñez N.V.A., Colpitts T.M., van der Ende-Metselaar H., Fikrig E., Diamond M.S., Wilschut J., Smit J.M. (2012). Antibodies against the envelope glycoprotein promote infectivity of immature dengue virus serotype 2. PLoS ONE.

[173] Wirawan M., Fibriansah G., Marzinek J.K., Lim X.X., Ng T.-S., Sim A.Y.L., Zhang Q., Kostyuchenko V.A., Shi J., Smith S.A. (2019). Mechanism of enhanced immature dengue virus attachment to endosomal membrane induced by prM antibody. Structure.

[174] Yam-Puc J.C., García-Cordero J., Calderón-Amador J., Donis-Maturano L., Cedillo-Barrón L., Flores-Romo L. (2015). Germinal center reaction following cutaneous dengue virus infection in immune-competent mice. Front. Immunol..

[175] Mukherjee S., Sirohi D., Dowd K.A., Chen Z., Diamond M.S., Kuhn R.J., Pierson T.C. (2016). Enhancing dengue virus maturation using a stable furin over-expressing cell line. Virology.

[176] Keelapang P., Nitatpattana N., Suphatrakul A., Punyahathaikul S., Sriburi R., Pulmanausahakul R., Pichyangkul S., Malasit P., Yoksan S., Sittisombut N. (2013). Generation and preclinical evaluation of a DENV-1/2 prM + E chimeric live attenuated vaccine candidate with enhanced prM cleavage. Vaccine.

[177] Oceguera L.F., Patiris P.J., Chiles R.E., Busch M.P., Tobler L.H., Hanson C. (2007). V Flavivirus serology by Western blot analysis. Am. J. Trop. Med. Hyg..

[178] Sjatha F., Kuwahara M., Sudiro T.M., Kameoka M., Konishi E. (2014). Evaluation of chimeric DNA vaccines consisting of premembrane and envelope genes of Japanese encephalitis and dengue viruses as a strategy for reducing induction of dengue virus infection-enhancing antibody response. Microbiol. Immunol..

[179] Wang Y., Si L., Luo Y., Guo X., Zhou J., Fang D., Yan H., Zeng G., Jiang L. (2015). Replacement of pr gene with Japanese encephalitis virus pr using reverse genetics reduces antibody-dependent enhancement of dengue virus 2 infection. Appl. Microbiol. Biotechnol..

[180] Wang Y., Si L.-L., Guo X.-L., Cui G., Fang D.-Y., Zhou J.-M., Yan H.-J., Jiang L.-F. (2017). Substitution of the precursor peptide prevents anti-prM antibody-mediated antibody-dependent enhancement of dengue virus infection. Virus Res..

[181] Roehrig J.T., Bolin R.A., Kelly R.G. (1998). Monoclonal antibody mapping of the envelope glycoprotein of the dengue 2 virus, Jamaica. Virology.

[182] Sukupolvi-Petty S., Austin S.K., Purtha W.E., Oliphant T., Nybakken G.E., Schlesinger J.J., Roehrig J.T., Gromowski G.D., Barrett A.D., Fremont D.H. (2007). Type- and subcomplex-specific neutralizing antibodies against domain III of dengue virus type 2 envelope protein recognize adjacent epitopes. J. Virol..

[183] Shrestha B., Brien J.D., Sukupolvi-Petty S., Austin S.K., Edeling M.A., Kim T., O’Brien K.M., Nelson C.A., Johnson S., Fremont D.H. (2010). The development of therapeutic antibodies that neutralize homologous and heterologous genotypes of dengue virus type 1. PLoS Pathog..

[184] Sukupolvi-Petty S., Austin S.K., Engle M., Brien J.D., Dowd K.A., Williams K.L., Johnson S., Rico-Hesse R., Harris E., Pierson T.C. (2010). Structure and function analysis of therapeutic monoclonal antibodies against dengue virus type 2. J. Virol..

[185] Gromowski G.D., Barrett A.D.T. (2007). Characterization of an antigenic site that contains a dominant, type-specific neutralization determinant on the envelope protein domain III (ED3) of dengue 2 virus. Virology.

[186] Brien J.D., Austin S.K., Sukupolvi-Petty S., O’Brien K.M., Johnson S., Fremont D.H., Diamond M.S. (2010). Genotype-specific neutralization and protection by antibodies against dengue virus type 3. J. Virol..

[187] Chen W.-H., Chou F.-P., Wang Y.-K., Huang S.-C., Cheng C.-H., Wu T.-K. (2017). Characterization and epitope mapping of Dengue virus type 1 specific monoclonal antibodies. Virol. J..

[188] Renner M., Flanagan A., Dejnirattisai W., Puttikhunt C., Kasinrerk W., Supasa P., Wongwiwat W., Chawansuntati K., Duangchinda T., Cowper A. (2018). Characterization of a potent and highly unusual minimally enhancing antibody directed against dengue virus. Nat. Immunol..

[189] Thullier P., Lafaye P., Mégret F., Deubel V., Jouan A., Mazié J. (1999). A recombinant Fab neutralizes dengue virus in vitro. J. Biotechnol..

[190] Thullier P., Demangel C., Bedouelle H., Me F., Jouan A., Deubel V., Mazie J.-C., Lafaye P. (2001). Mapping of a dengue virus neutralizing epitope critical for the infectivity of all serotypes: Insight into the neutralization mechanism. J. Gen. Virol..

[191] Lisova O., Hardy F., Petit V., Bedouelle H. (2007). Mapping to completeness and transplantation of a group-specific, discontinuous, neutralizing epitope in the envelope protein of dengue virus. J. Gen. Virol..

[192] Rajamanonmani R., Nkenfou C., Clancy P., Yau Y.H., Shochat S.G., Sukupolvi-Petty S., Schul W., Diamond M.S., Vasudevan S.G., Lescar J. (2009). On a mouse monoclonal antibody that neutralizes all four dengue virus serotypes. J. Gen. Virol..

[193] Gromowski G.D., Roehrig J.T., Diamond M.S., Lee J.C., Pitcher T.J., Barrett A.D.T. (2010). Mutations of an antibody binding energy hot spot on domain III of the dengue 2 envelope glycoprotein exploited for neutralization escape. Virology.

[194] Megret F., Hugnot J.P., Falconar A., Gentry M.K., Morens D.M., Murray J.M., Schlesinger J.I., Wright P.J., Young P., Van Regenmortel M.H.V. (1992). Use of recombinant fusion proteins and monoclonal antibodies to define linear and discontinuous antigenic sites on the dengue virus envelope glycoprotein. Virology.

[195] Cockburn J.J.B., Sanchez M.E.N., Fretes N., Urvoas A., Staropoli I., Kikuti C.M., Coffey L.L., Seisdedos F.A., Bedouelle H., Rey F.A. (2012). Mechanism of dengue virus broad cross-neutralization by a monoclonal antibody. Structure.

[196] Pierson T.C., Kuhn R.J. (2012). Capturing a virus while it catches its breath. Structure.

[197] Tharakaraman K., Robinson L.N., Hatas A., Chen Y.L., Siyue L., Raguram S., Sasisekharan V., Wogan G.N., Sasisekharan R. (2013). Redesign of a cross-reactive antibody to dengue virus with broad-spectrum activity and increased in vivo potency. Proc. Natl. Acad. Sci. USA.

[198] Robinson L.N., Tharakaraman K., Rowley K.J., Costa V.V., Chan K.R., Wong Y.H., Ong L.C., Tan H.C., Koch T., Cain D. (2015). Structure-guided design of an anti-dengue antibody directed to a non-immunodominant epitope. Cell.

[199] Midgley C.M., Flanagan A., Tran H.B., Dejnirattisai W., Chawansuntati K., Jumnainsong A., Wongwiwat W., Duangchinda T., Mongkolsapaya J., Grimes J.M. (2012). Structural analysis of a dengue cross-reactive antibody complexed with envelope domain III reveals the molecular basis of cross-reactivity. J. Immunol..

[200] Li X.-Q., Qiu L.-W., Chen Y., Wen K., Cai J.-P., Chen J., Pan Y.-X., Li J., Hu D.-M., Huang Y.-F. (2013). Dengue virus envelope domain III immunization elicits predominantly cross-reactive, poorly neutralizing antibodies localized to the AB loop: Implications for dengue vaccine design. J. Gen. Virol..

[201] Li J., Watterson D., Chang C.-W., Che X.-Y., Li X.-Q., Ericsson D.J., Qiu L.-W., Cai J.-P., Chen J., Fry S.R. (2018). Structural and functional characterization of a cross-reactive dengue virus neutralizing antibody that recognizes a cryptic epitope. Structure.

[202] Wahala W.M.P.B., Huang C., Butrapet S., White L.J., de Silva A.M. (2012). Recombinant dengue type 2 viruses with altered E protein domain III epitopes are efficiently neutralized by human immune sera. J. Virol..

[203] Midgley C.M., Bajwa-Joseph M., Vasanawathana S., Limpitikul W., Wills B., Flanagan A., Waiyaiya E., Tran H.B., Cowper A.E., Chotiyarnwong P. (2011). An in-depth analysis of original antigenic sin in dengue virus infection. J. Virol..

[204] Williams K.L., Wahala W.M.P.B., Orozco S., de Silva A.M., Harris E. (2012). Antibodies targeting dengue virus envelope domain III are not required for serotype-specific protection or prevention of enhancement in vivo. Virology.

[205] Guzman M.G., Hermida L., Bernardo L., Ramirez R., Guillén G. (2010). Domain III of the envelope protein as a dengue vaccine target. Expert Rev. Vaccines.

[206] Fonseca B.A.L., Khoshnood K., Shope R.E., Mason P.W. (1991). Flavivirus type-specific antigens produced from fusions of a portion of the E protein gene with the Escherichia coli trpe gene. Am. J. Trop. Med. Hyg..

[207] Simmons M., Hayes C.G., Wu S.J., Nelson W.M. (1998). Evaluation of the protective efficacy of a recombinant dengue envelope B domain fusion protein against dengue 2 virus infection in mice. Am. J. Trop. Med. Hyg..

[208] Hermida L., Rodríguez R., Lazo L., Silva R., Zulueta A., Chinea G., López C., Guzmán M.G., Guillén G. (2004). A dengue-2 Envelope fragment inserted within the structure of the P64k meningococcal protein carrier enables a functional immune response against the virus in mice. J. Virol. Methods.

[209] Chiang C.-Y., Liu S.-J., Tsai J.-P., Li Y.-S., Chen M.-Y., Liu H.-H., Chong P., Leng C.-H., Chen H.-W. (2011). A Novel single-dose dengue subunit vaccine induces memory immune responses. PLoS ONE.

[210] Mota J., Acosta M., Argotte R., Figueroa R., Méndez A., Ramos C. (2005). Induction of protective antibodies against dengue virus by tetravalent DNA immunization of mice with domain III of the envelope protein. Vaccine.

[211] Azevedo A.S., Yamamura A.M.Y., Freire M.S., Trindade G.F., Bonaldo M., Galler R., Alves A.M.B. (2011). DNA vaccines against dengue virus type 2 based on truncate envelope protein or its domain III. PLoS ONE.

[212] Poggianella M., Campos J.L.S., Chan K.R., Tan H.C., Bestagno M., Ooi E.E., Burrone O.R. (2015). Dengue E protein domain III-based DNA immunisation induces strong antibody responses to all four viral serotypes. PLoS Negl. Trop. Dis..

[213] Khanam S., Khanna N., Swaminathan S. (2006). Induction of neutralizing antibodies and T cell responses by dengue virus type 2 envelope domain III encoded by plasmid and adenoviral vectors. Vaccine.

[214] Brandler S., Lucas-Hourani M., Moris A., Frenkiel M.-P., Combredet C., Février M., Bedouelle H., Schwartz O., Desprès P., Tangy F. (2007). Pediatric measles vaccine expressing a dengue antigen induces durable serotype-specific neutralizing antibodies to dengue virus. PLoS Negl. Trop. Dis..

[215] Arora U., Tyagi P., Swaminathan S., Khanna N. (2013). Virus-like particles displaying envelope domain III of dengue virus type 2 induce virus-specific antibody response in mice. Vaccine.

[216] Chua A.J., Vituret C., Tan M.L., Gonzalez G., Boulanger P., Ng M.-L., Hong S.-S. (2013). A novel platform for virus-like particle-display of flaviviral envelope domain III: Induction of Dengue and West Nile virus neutralizing antibodies. Virol. J..

[217] Lazo L., Izquierdo A., Suzarte E., Gil L., Valdés I., Marcos E., Álvarez M., Romero Y., Guzmán M.G., Guillén G. (2014). Evaluation in mice of the immunogenicity and protective efficacy of a tetravalent subunit vaccine candidate against dengue virus. Microbiol. Immunol..

[218] Zhao H., Jiang T., Zhou X.-Z., Deng Y.-Q., Li X.-F., Chen S.-P., Zhu S.-Y., Zhou X., Qin E.-D., Qin C.-F. (2014). Induction of neutralizing antibodies against four serotypes of dengue viruses by MixBiEDIII, a tetravalent dengue vaccine. PLoS ONE.

[219] Ramasamy V., Arora U., Shukla R., Poddar A., Shanmugam R.K., White L.J., Mattocks M.M., Raut R., Perween A., Tyagi P. (2018). A tetravalent virus-like particle vaccine designed to display domain III of dengue envelope proteins induces multi-serotype neutralizing antibodies in mice and macaques which confer protection against antibody dependent enhancement in AG129 mice. PLoS Negl. Trop. Dis..

[220] Hermida L., Bernardo L., Martín J., Alvarez M., Prado I., López C., Sierra B.D.L.C., Martínez R., Rodríguez R., Zulueta A. (2006). A recombinant fusion protein containing the domain III of the dengue-2 envelope protein is immunogenic and protective in nonhuman primates. Vaccine.

[221] Valdés I., Hermida L., Martín J., Menéndez T., Gil L., Lazo L., Castro J., Niebla O., López C., Bernardo L. (2009). Immunological evaluation in nonhuman primates of formulations based on the chimeric protein P64k-domain III of dengue 2 and two components of Neisseria meningitidis. Vaccine.

[222] McBurney S.P., Sunshine J.E., Gabriel S., Huynh J.P., Sutton W.F., Fuller D.H., Haigwood N.L., Messer W.B. (2016). Evaluation of protection induced by a dengue virus serotype 2 envelope domain III protein scaffold/DNA vaccine in non-human primates. Vaccine.

[223] Block O.K.T., Shanaka W.W., Rodrigo I., Quinn M., Jin X., Rose R.C., Schlesinger J.J. (2010). A tetravalent recombinant dengue domain III protein vaccine stimulates neutralizing and enhancing antibodies in mice. Vaccine.

[224] Chiang C.-Y., Pan C.-H., Hsieh C.-H., Tsai J.-P., Chen M.-Y., Liu H.-H., Liu S.-J., Chong P., Leng C.-H., Chen H.-W. (2013). Lipidated dengue-2 envelope protein domain III independently stimulates long-lasting neutralizing antibodies and reduces the risk of antibody-dependent enhancement. PLoS Negl. Trop. Dis..

[225] Rajpoot R.K., Shukla R., Arora U., Swaminathan S., Khanna N. (2018). Dengue envelope-based ‘four-in-one’ virus-like particles produced using *Pichia pastoris* induce enhancement-lacking, domain III-directed tetravalent neutralising antibodies in mice. Sci. Rep..

[226] Shukla R., Rajpoot R.K., Arora U., Poddar A., Swaminathan S., Khanna N. (2018). *Pichia pastoris*-expressed bivalent virus-like particulate vaccine induces domain III-focused bivalent neutralizing antibodies without antibody-dependent enhancement in vivo. Front. Microbiol..

[227] Leng C.-H., Liu S.-J., Tsai J.-P., Li Y.-S., Chen M.-Y., Liu H.-H., Lien S.-P., Yueh A., Hsiao K.-N., Lai L.-W. (2009). A novel dengue vaccine candidate that induces cross-neutralizing antibodies and memory immunity. Microbes Infect..

[228] Frei J.C., Wirchnianski A.S., Govero J., Vergnolle O., Dowd K.A., Pierson T.C., Kielian M., Girvin M.E., Diamond M.S., Lai J.R. (2018). Engineered dengue virus domain III proteins elicit cross-neutralizing antibody responses in mice. J. Virol..

[229] Valdés I., Hermida L., Gil L., Lazo L., Castro J., Martín J., Bernardo L., López C., Niebla O., Menéndez T. (2010). Heterologous prime-boost strategy in non-human primates combining the infective dengue virus and a recombinant protein in a formulation suitable for human use. Int. J. Infect. Dis..

[230] Zlatkovic J., Stiasny K., Heinz F.X. (2011). Immunodominance and functional activities of antibody responses to inactivated West Nile virus and recombinant subunit vaccines in mice. J. Virol..

[231] Teoh E.P., Kukkaro P., Teo E.W., Lim A.P.C., Tan T.T., Yip A., Schul W., Aung M., Kostyuchenko V.A., Leo Y.S. (2012). The structural basis for serotype-specific neutralization of dengue virus by a human antibody. Sci. Transl. Med..

[232] Fibriansah G., Tan J.L., Smith S.A., Alwis A.R., Ng T., Kostyuchenko V.A., Ibarra K.D., Wang J., Harris E., Silva A. (2014). A potent anti-dengue human antibody preferentially recognizes the conformation of E protein monomers assembled on the virus surface. EMBO Mol. Med..

[233] Fibriansah G., Ibarra K.D., Ng T.-S., Smith S.A., Tan J.L., Lim X.-N., Ooi J.S.G., Kostyuchenko V.A., Wang J., de Silva A.M. (2015). Cryo-EM structure of an antibody that neutralizes dengue virus type 2 by locking E protein dimers. Science.

[234] Gallichotte E.N., Baric T.J., Yount B.L., Widman D.G., Durbin A., Whitehead S., Baric R.S., de Silva A.M. (2018). Human dengue virus serotype 2 neutralizing antibodies target two distinct quaternary epitopes. PLoS Pathog..

[235] Fibriansah G., Tan J.L., Smith S.A., de Alwis R., Ng T.-S., Kostyuchenko V.A., Jadi R.S., Kukkaro P., de Silva A.M., Crowe J.E. (2015). A highly potent human antibody neutralizes dengue virus serotype 3 by binding across three surface proteins. Nat. Commun..

[236] Rouvinski A., Guardado-Calvo P., Barba-Spaeth G., Duquerroy S., Vaney M.-C., Kikuti C.M., Sanchez M.E.N., Dejnirattisai W., Wongwiwat W., Haouz A. (2015). Recognition determinants of broadly neutralizing human antibodies against dengue viruses. Nature.

[237] Nivarthi U.K., Kose N., Sapparapu G., Widman D., Gallichotte E., Pfaff J.M., Doranz B.J., Weiskopf D., Sette A., Durbin A.P. (2017). Mapping the human memory B cell and serum neutralizing antibody responses to dengue virus serotype 4 infection and vaccination. J. Virol..

[238] Andrade D.V., Warnes C., Young E., Katzelnick L.C., Balmaseda A., de Silva A.M., Baric R.S., Harris E. (2019). Tracking the polyclonal neutralizing antibody response to a dengue virus serotype 1 type-specific epitope across two populations in Asia and the Americas. Sci. Rep..

[239] Swanstrom J.A., Nivarthi U.K., Patel B., Delacruz M.J., Yount B., Widman D.G., Durbin A.P., Whitehead S.S., De Silva A.M., Baric R.S. (2019). Beyond neutralizing antibody levels: The epitope specificity of antibodies induced by national institutes of health monovalent dengue virus vaccines. J. Infect. Dis..

[240] Gallichotte E.N., Widman D.G., Yount B.L., Wahala W.M., Durbin A., Whitehead S., Sariol C.A., Crowe J.E., de Silva A.M., Baric R.S. (2015). A new quaternary structure epitope on dengue virus serotype 2 is the target of durable type-specific neutralizing antibodies. mBio.

[241] Messer W.B., Yount B.L., Royal S.R., de Alwis R., Widman D.G., Smith S.A., Crowe J.E., Pfaff J.M., Kahle K.M., Doranz B.J. (2016). Functional transplant of a dengue virus serotype 3 (DENV3)-specific human monoclonal antibody epitope into DENV1. J. Virol..

[242] Andrade D.V., Katzelnick L.C., Widman D.G., Balmaseda A., de Silva A.M., Baric R.S., Harris E. (2017). Analysis of individuals from a dengue-endemic region helps define the footprint and repertoire of antibodies targeting dengue virus 3 type-specific epitopes. mBio.

[243] Widman D.G., Young E., Nivarthi U., Swanstrom J.A., Royal S.R., Yount B.L., Debbink K., Begley M., Marcet S., Durbin A. (2017). Transplantation of a quaternary structure neutralizing antibody epitope from dengue virus serotype 3 into serotype 4. Sci. Rep..

[244] Young E., Carnahan R.H., Andrade D.V., Kose N., Nargi R.S., Fritch E.J., Munt J.E., Doyle M.P., White L., Baric T.J. (2020). Identification of dengue virus serotype 3 specific antigenic sites targeted by neutralizing human antibodies. Cell Host Microbe.

[245] Rouvinski A., Dejnirattisai W., Guardado-Calvo P., Vaney M.-C., Sharma A., Duquerroy S., Supasa P., Wongwiwat W., Haouz A., Barba-Spaeth G. (2017). Covalently linked dengue virus envelope glycoprotein dimers reduce exposure of the immunodominant fusion loop epitope. Nat. Commun..

[246] Barba-Spaeth G., Dejnirattisai W., Rouvinski A., Vaney M.-C., Medits I., Sharma A., Simon-Lorière E., Sakuntabhai A., Cao-Lormeau V.-M., Haouz A. (2016). Structural basis of potent Zika–dengue virus antibody cross-neutralization. Nature.

[247] Swanstrom J.A., Plante J.A., Plante K.S., Young E.F., McGowan E., Gallichotte E.N., Widman D.G., Heise M.T., de Silva A.M., Baric R.S. (2016). Dengue virus envelope dimer epitope monoclonal antibodies isolated from dengue patients are protective against Zika virus. mBio.

[248] Fernandez E., Dejnirattisai W., Cao B., Scheaffer S.M., Supasa P., Wongwiwat W., Esakky P., Drury A., Mongkolsapaya J., Moley K.H. (2017). Human antibodies to the dengue virus E-dimer epitope have therapeutic activity against Zika virus infection. Nat. Immunol..

[249] Abbink P., Larocca R.A., Dejnirattisai W., Peterson R., Nkolola J.P., Borducchi E.N., Supasa P., Mongkolsapaya J., Screaton G.R., Barouch D.H. (2018). Therapeutic and protective efficacy of a dengue antibody against Zika infection in rhesus monkeys. Nat. Med..

[250] Tripathi N.K., Shrivastava A. (2018). Recent developments in recombinant protein-based dengue vaccines. Front. Immunol..

[251] Metz S.W., Gallichotte E.N., Brackbill A., Premkumar L., Miley M.J., Baric R., de Silva A.M. (2017). In vitro assembly and stabilization of dengue and Zika virus envelope protein homo-dimers. Sci. Rep..

[252] Campos J.L.C., Marchese S., Rana J., Mossenta M., Poggianella M., Bestagno M., Burrone O.R. (2017). Temperature-dependent folding allows stable dimerization of secretory and virus-associated E proteins of Dengue and Zika viruses in mammalian cells. Sci. Rep..

[253] Glasner D.R., Puerta-Guardo H., Beatty P.R., Harris E. (2018). The good, the bad, and the shocking: The multiple roles of dengue virus nonstructural protein 1 in protection and pathogenesis. Annu. Rev. Virol..

[254] Winkler G., Randolph V.B., Cleaves G.R., Ryan T.E., Stollar V. (1988). Evidence that the mature form of the flavivirus nonstructural protein NS1 is a dimer. Virology.

[255] Akey D.L., Brown W.C., Dutta S., Konwerski J., Jose J., Jurkiw T.J., Delproposto J., Ogata C.M., Skiniotis G., Kuhn R.J. (2014). Flavivirus NS1 crystal structures reveal a surface for membrane association and regions of interaction with the immune system. Science.

[256] Winkler G., Maxwell S.E., Ruemmler C., Stollar V. (1989). Newly synthesized dengue-2 virus nonstructural protein NS1 is a soluble protein but becomes partially hydrophobic and membrane-associated after dimerization. Virology.

[257] Jacobs M.G., Robinson P.J., Bletchly C., Mackenzie J.M., Young P.R. (2000). Dengue virus nonstructural protein 1 is expressed in a glycosyl-phosphatidylinositol-linked form that is capable of signal transduction. FASEB J..

[258] Flamand M., Megret F., Mathieu M., Lepault J., Rey F.A., Deubel V. (1999). Dengue virus type 1 nonstructural glycoprotein NS1 is secreted from mammalian cells as a soluble hexamer in a glycosylation-dependent fashion. J. Virol..

[259] Young P.R., Hilditch P.A., Bletchly C., Halloran W. (2000). An antigen capture enzyme-linked immunosorbent assay reveals high levels of the dengue virus protein NS1 in the sera of infected patients. J. Clin. Microbiol..

[260] Alcon S., Talarmin A., Debruyne M., Falconar A., Deubel V., Flamand M. (2002). Enzyme-linked immunosorbent assay specific to Dengue virus type 1 nonstructural protein NS1 reveals circulation of the antigen in the blood during the acute phase of disease in patients experiencing primary or secondary infections. J. Clin. Microbiol..

[261] Libraty D.H., Young P.R., Pickering D., Endy T.P., Kalayanarooj S., Green S., Vaughn D.W., Nisalak A., Ennis F.A., Rothman A.L. (2002). High circulating levels of the dengue virus nonstructural protein NS1 early in dengue illness correlate with the development of dengue hemorrhagic fever. J. Infect. Dis..

[262] Beatty P.R., Puerta-Guardo H., Killingbeck S.S., Glasner D.R., Hopkins K., Harris E. (2015). Dengue virus NS1 triggers endothelial permeability and vascular leak that is prevented by NS1 vaccination. Sci. Transl. Med..

[263] Modhiran N., Watterson D., Muller D.A., Panetta A.K., Sester D.P., Liu L., Hume D.A., Stacey K.J., Young P.R. (2015). Dengue virus NS1 protein activates cells via Toll-like receptor 4 and disrupts endothelial cell monolayer integrity. Sci. Transl. Med..

[264] Puerta-Guardo H., Glasner D.R., Harris E. (2016). Dengue virus NS1 disrupts the endothelial glycocalyx, leading to hyperpermeability. PLoS Pathog..

[265] Puerta-Guardo H., Glasner D.R., Espinosa D.A., Biering S.B., Patana M., Ratnasiri K., Wang C., Beatty P.R., Harris E. (2019). Flavivirus NS1 triggers tissue-specific vascular endothelial dysfunction reflecting disease tropism. Cell Rep..

[266] Wang C., Puerta-Guardo H., Biering S.B., Glasner D.R., Tran E.B., Patana M., Gomberg T.A., Malvar C., Lo N.T., Espinosa D.A. (2019). Endocytosis of flavivirus NS1 is required for NS1-mediated endothelial hyperpermeability and is abolished by a single N-glycosylation site mutation. PLoS Pathog..

[267] Glasner D.R., Ratnasiri K., Puerta-Guardo H., Espinosa D.A., Beatty P.R., Harris E. (2017). Dengue virus NS1 cytokine-independent vascular leak is dependent on endothelial glycocalyx components. PLoS Pathog..

[268] Lin S.W., Chuang Y.C., Lin Y.S., Lei H.Y., Liu H.S., Yeh T.M. (2012). Dengue virus nonstructural protein NS1 binds to prothrombin/thrombin and inhibits prothrombin activation. J. Infect..

[269] Chao C.-H., Wu W.-C., Lai Y.-C., Tsai P.-J., Perng G.-C., Lin Y.-S., Yeh T.-M. (2019). Dengue virus nonstructural protein 1 activates platelets via Toll-like receptor 4, leading to thrombocytopenia and hemorrhage. PLoS Pathog..

[270] Kuno G., Vorndam A.V., Gubler D.J., Gómez I. (1990). Study of anti-dengue NS1 antibody by Western blot. J. Med. Virol..

[271] Churdboonchart V., Bhamarapravati N., Peampramprecha S., Sirinavin S. (1991). Antibodies against dengue viral proteins in primary and secondary dengue hemorrhagic fever. Am. J. Trop. Med. Hyg..

[272] Shu P.Y., Chen L.K., Chang S.F., Yueh Y.Y., Chow L., Chien L.J., Chin C., Lin T.H., Huang J.H. (2000). Dengue NS1-specific antibody responses: Isotype distribution and serotyping in patients with Dengue fever and Dengue hemorrhagic fever. J. Med. Virol..

[273] Valdés K., Alvarez M., Pupo M., Vázquez S., Rodríguez R., Guzmán M.G. (2000). Human Dengue antibodies against structural and nonstructural proteins. Clin. Diagn. Lab. Immunol..

[274] Hertz T., Beatty P.R., MacMillen Z., Killingbeck S.S., Wang C., Harris E. (2017). Antibody epitopes identified in critical regions of dengue virus nonstructural 1 protein in mouse vaccination and natural human infections. J. Immunol..

[275] Jayathilaka D., Gomes L., Jeewandara C., Jayarathna G.S.B., Herath D., Perera P.A., Fernando S., Wijewickrama A., Hardman C.S., Ogg G.S. (2018). Role of NS1 antibodies in the pathogenesis of acute secondary dengue infection. Nat. Commun..

[276] Falkler W.A., Diwan A.R., Halstead S.B. (1973). Human antibody to dengue soluble complement-fixing (SCF) antigens. J. Immunol..

[277] Twiddy S.S., Woelk C.H., Holmes E.C. (2002). Phylogenetic evidence for adaptive evolution of dengue viruses in nature. J. Gen. Virol..

[278] Falconar A.K.I., Young P.R., Miles M.A. (1994). Precise location of sequential dengue virus subcomplex and complex B cell epitopes on the nonstructural-1 glycoprotein. Arch. Virol..

[279] García G., Vaughn D.W., Del Angel R.M. (1997). Recognition of synthetic oligopeptides from nonstructural proteins NS1 and NS3 of Dengue-4 virus by sera from Dengue virus—Infected children. Am. J. Trop. Med. Hyg..

[280] Falconar A.K.I. (2007). Antibody responses are generated to immunodominant ELK/KLE-type motifs on the nonstructural-1 glycoprotein during live dengue virus infections in mice and humans: Implications for diagnosis, pathogenesis, and vaccine design. Clin. Vaccine Immunol..

[281] Jiang L., Zhou J.-M., Yin Y., Fang D.-Y., Tang Y.-X., Jiang L.-F. (2010). Selection and identification of B-cell epitope on NS1 protein of dengue virus type 2. Virus Res..

[282] Chen Y., Pan Y., Guo Y., Qiu L., Ding X., Che X. (2010). Comprehensive mapping of immunodominant and conserved serotype- and group-specific B-cell epitopes of nonstructural protein 1 from dengue virus type 1. Virology.

[283] Henriques H.R., Rampazo E.V., Gonçalves A.J.S., Vicentin E.C.M., Amorim J.H., Panatieri R.H., Amorim K.N.S., Yamamoto M.M., Ferreira L.C.S., Alves A.M.B. (2013). Targeting the non-structural protein 1 from dengue virus to a dendritic cell population confers protective immunity to lethal virus challenge. PLoS Negl. Trop. Dis..

[284] Costa S.M., Paes M.V., Barreto D.F., Pinhão A.T., Barth O.M., Queiroz J.L.S., Armôa G.R.G., Freire M.S., Alves A.M.B. (2006). Protection against dengue type 2 virus induced in mice immunized with a DNA plasmid encoding the non-structural 1 (NS1) gene fused to the tissue plasminogen activator signal sequence. Vaccine.

[285] Amorim J.H., Diniz M.O., Cariri F.A.M.O., Rodrigues J.F., Bizerra R.S.P., Gonçalves A.J.S., de Barcelos Alves A.M., de Souza Ferreira L.C. (2012). Protective immunity to DENV2 after immunization with a recombinant NS1 protein using a genetically detoxified heat-labile toxin as an adjuvant. Vaccine.

[286] Rocha L., Alves R., Caetano B., Pereira L., Mitsunari T., Amorim J., Polatto J., Botosso V., Gallina N., Palacios R. (2017). Epitope sequences in Dengue virus NS1 protein identified by monoclonal antibodies. Antibodies.

[287] Henchal E.A., Henchal L.S., Schlesinger J.J. (1988). Synergistic interactions of Anti-NS1 monoclonal antibodies protect passively immunized mice from lethal challenge with Dengue 2 virus. J. Gen. Virol..

[288] Schlesinger J.J., Brandriss M.W., Walsh E.E. (1985). Protection against 17D yellow fever encephalitis in mice by passive transfer of monoclonal antibodies to the nonstructural glycoprotein gp48 and by active immunization with gp48. J. Immunol..

[289] García G., Arango M., Pérez A.B., Fonte L., Sierra B., Rodríguez-Roche R., Aguirre E., Fiterre I., Guzmán M.G. (2006). Antibodies from patients with dengue viral infection mediate cellular cytotoxicity. J. Clin. Virol..

[290] Wan S.-W., Chen P.-W., Chen C.-Y., Lai Y.-C., Chu Y.-T., Hung C.-Y., Lee H., Wu H.F., Chuang Y.-C., Lin J. (2017). Therapeutic effects of monoclonal antibody against Dengue virus NS1 in a STAT1 knockout mouse model of dengue infection. J. Immunol..

[291] Chung K.M., Thompson B.S., Fremont D.H., Diamond M.S. (2007). Antibody recognition of cell surface-associated NS1 triggers Fc-gamma receptor-mediated phagocytosis and clearance of West Nile Virus-infected cells. J. Virol..

[292] Falgout B., Bray M., Schlesinger J.J., Lai C.-J. (1990). Immunization of mice with recombinant vaccinia virus expressing authentic dengue virus nonstructural protein NS1 protects against lethal dengue virus encephalitis. J. Virol..

[293] Wu S.-F., Liao C.-L., Lin Y.-L., Yeh C.-T., Chen L.-K., Huang Y.-F., Chou H.-Y., Huang J.-L., Shaio M.-F., Sytwu H.-K. (2003). Evaluation of protective efficacy and immune mechanisms of using a non-structural protein NS1 in DNA vaccine against dengue 2 virus in mice. Vaccine.

[294] Costa S.M., Azevedo A.S., Paes M.V., Sarges F.S., Freire M.S., Alves A.M.B. (2007). DNA vaccines against dengue virus based on the ns1 gene: The influence of different signal sequences on the protein expression and its correlation to the immune response elicited in mice. Virology.

[295] Falgout B., Chanock R., Lai C.-J. (1989). Proper processing of dengue virus nonstructural glycoprotein NS1 Requires the N-terminal hydrophobic signal sequence and the downstream nonstructural protein NS2a. J. Virol..

[296] Costa S.M., Freire M.S., Alves A.M.B. (2006). DNA vaccine against the non-structural 1 protein (NS1) of dengue 2 virus. Vaccine.

[297] Espinosa D.A., Beatty P.R., Reiner G.L., Sivick K.E., Glickman L.H., Dubensky T.W., Harris E. (2019). Cyclic dinucleotide-adjuvanted dengue virus nonstructural protein 1 induces protective antibody and T cell responses. J. Immunol..

[298] Ambuel Y., Young G., Brewoo J.N., Paykel J., Weisgrau K.L., Rakasz E.G., Haller A.A., Royals M., Huang C.Y.-H., Capuano S. (2014). A rapid immunization strategy with a live-attenuated tetravalent dengue vaccine elicits protective neutralizing antibody responses in non-human primates. Front. Immunol..

[299] Sharma M., Glasner D.R., Watkins H., Puerta-Guardo H., Kassa Y., Egan M.A., Dean H., Harris E. (2020). Magnitude and functionality of the NS1-specific antibody response elicited by a live-attenuated tetravalent dengue vaccine candidate. J. Infect. Dis..

[300] Nascimento E.J.M., George J.K., Velasco M., Bonaparte M.I., Zheng L., DiazGranados C.A., Marques E.T.A., Huleatt J.W. (2018). Development of an anti-dengue NS1 IgG ELISA to evaluate exposure to dengue virus. J. Virol. Methods.

[301] Falconar A.K.I. (1997). The dengue virus nonstructural-1 protein (NS1) generates antibodies to common epitopes on human blood clotting, integrin/adhesin proteins and binds to human endothelial cells: Potential implications in haemorrhagic fever pathogenesis. Arch. Virol..

[302] Lin C.-F., Lei H.-Y., Shiau A.-L., Liu C.-C., Liu H.-S., Yeh T.-M., Chen S.-H., Lin Y.-S. (2003). Antibodies from dengue patient sera cross-react with endothelial cells and induce damage. J. Med. Virol..

[303] Lin C.-F., Lei H.-Y., Shiau A.-L., Liu H.-S., Yeh T.-M., Chen S.-H., Liu C.-C., Chiu S.-C., Lin Y.-S. (2002). Endothelial cell apoptosis induced by antibodies against dengue virus nonstructural protein 1 via production of nitric oxide. J. Immunol..

[304] Lin C.-F., Chiu S.-C., Hsiao Y.-L., Wan S.-W., Lei H.-Y., Shiau A.-L., Liu H.-S., Yeh T.-M., Chen S.-H., Liu C.-C. (2005). Expression of cytokine, chemokine, and adhesion molecules during endothelial cell activation induced by antibodies against dengue virus nonstructural protein 1. J. Immunol..

[305] Wan S.W., Yang Y.W., Chu Y.T., Lin C.F., Chang C.P., Yeh T.M., Anderson R., Lin Y.S. (2016). Anti-dengue virus nonstructural protein 1 antibodies contribute to platelet phagocytosis by macrophages. Thromb. Haemost..

[306] Cheng H.-J., Lei H.-Y., Lin C.-F., Luo Y.-H., Wan S.-W., Liu H.-S., Yeh T.-M., Lin Y.-S. (2009). Anti-dengue virus nonstructural protein 1 antibodies recognize protein disulfide isomerase on platelets and inhibit platelet aggregation. Mol. Immunol..

[307] Chen M.-C., Lin C.-F., Lei H.-Y., Lin S.-C., Liu H.-S., Yeh T.-M., Anderson R., Lin Y.-S. (2009). Deletion of the C-terminal region of dengue virus nonstructural protein 1 (NS1) abolishes anti-NS1-mediated platelet dysfunction and bleeding tendency. J. Immunol..

[308] Chuang Y.-C., Lei H.-Y., Lin Y.-S., Liu H.-S., Wu H.-L., Yeh T.-M. (2011). Dengue virus-induced autoantibodies bind to plasminogen and enhance its activation. J. Immunol..

[309] Chuang Y.-C., Lin Y.-S., Liu H.-S., Yeh T.-M. (2014). Molecular mimicry between dengue virus and coagulation factors induces antibodies to inhibit thrombin activity and enhance fibrinolysis. J. Virol..

[310] Chuang Y.-C., Lin J., Lin Y.-S., Wang S., Yeh T.-M. (2016). Dengue virus nonstructural protein 1-induced antibodies cross-react with human plasminogen and enhance its activation. J. Immunol..

[311] Lin C.-F., Wan S.-W., Chen M.-C., Lin S.-C., Cheng C.-C., Chiu S.-C., Hsiao Y.-L., Lei H.-Y., Liu H.-S., Yeh T.-M. (2008). Liver injury caused by antibodies against dengue virus nonstructural protein 1 in a murine model. Lab. Investig..

[312] Lin C.-F., Lei H.-Y., Liu C.-C., Liu H.-S., Yeh T.-M., Wang S.-T., Yang T.-I., Sheu F.-C., Kuo C.-F., Lin Y.-S. (2001). Generation of IgM anti-platelet autoantibody in dengue patients. J. Med. Virol..

[313] Saito M., Oishi K., Inoue S., Dimaano E.M., Alera M.T.P., Robles A.M.P., Estrella B.D., Kumatori A., Moji K., Alonzo M.T. (2004). Association of increased platelet-associated immunoglobulins with thrombocytopenia and the severity of disease in secondary dengue virus infections. Clin. Exp. Immunol..

[314] Wan S.-W., Lin C.-F., Chen M.-C., Lei H.-Y., Liu H.-S., Yeh T.-M., Liu C.-C., Lin Y.-S., Wan S.-W., Lin C.-F. (2008). C-terminal region of dengue virus nonstructural protein 1 is involved in endothelial cell cross-reactivity via molecular mimicry. Am. J. Infect. Dis..

[315] Cheng H.-J., Lin C.-F., Lei H.-Y., Liu H.-S., Yeh T.-M., Luo Y.-H., Lin Y.-S. (2009). Proteomic analysis of endothelial cell autoantigens recognized by anti-dengue virus nonstructural protein 1 antibodies. Exp. Biol. Med..

[316] Liu I.-J., Chiu C.-Y., Chen Y.-C., Wu H.-C. (2011). Molecular mimicry of human endothelial cell antigen by autoantibodies to nonstructural protein 1 of dengue virus. J. Biol. Chem..

[317] Waterhouse A., Bertoni M., Bienert S., Studer G., Tauriello G., Gumienny R., Heer F.T., de Beer T.A.P., Rempfer C., Bordoli L. (2018). SWISS-MODEL: Homology modelling of protein structures and complexes. Nucleic Acids Res..

[318] Wan S.-W., Lu Y.-T., Huang C.-H., Lin C.-F., Anderson R., Liu H.-S., Yeh T.-M., Yen Y.-T., Wu-Hsieh B.A., Lin Y.-S. (2014). Protection against dengue virus infection in mice by administration of antibodies against modified nonstructural protein 1. PLoS ONE.

[319] Liu J., Liu Y., Nie K., Du S., Qiu J., Pang X., Wang P., Cheng G. (2016). Flavivirus NS1 protein in infected host sera enhances viral acquisition by mosquitoes. Nat. Microbiol..

[320] Lai Y.-C., Chuang Y.-C., Liu C.-C., Ho T.-S., Lin Y.-S., Anderson R., Yeh T.-M. (2017). Antibodies against modified NS1 wing domain peptide protect against dengue virus infection. Sci. Rep..

[321] Zivny J., DeFronzo M., Jarry W., Jameson J., Cruz J., Ennis F.A., Rothman A.L. (1999). Partial agonist effect influences the CTL response to a heterologous dengue virus serotype. J. Immunol..

[322] Zivny J., Kurane I., Leporati A.M., Ibe M., Takiguchi M., Zeng L.L., Brinton M.A., Ennis F.A. (1995). A single nine-amino acid peptide induces virus-specific, CD8+ human cytotoxic T lymphocyte clones of heterogeneous serotype specificities. J. Exp. Med..

[323] Zivna I., Green S., Vaughn D.W., Kalayanarooj S., Stephens H.A.F., Chandanayingyong D., Nisalak A., Ennis F.A., Rothman A.L. (2002). T cell responses to an HLA-B*07-restricted epitope on the dengue NS3 protein correlate with disease severity. J. Immunol..

[324] Friberg H., Bashyam H., Toyosaki-Maeda T., Potts J.A., Greenough T., Kalayanarooj S., Gibbons R.V., Nisalak A., Srikiatkhachorn A., Green S. (2011). Cross-reactivity and expansion of dengue-specific T cells during acute primary and secondary infections in humans. Sci. Rep..

[325] Friberg H., Burns L., Woda M., Kalayanarooj S., Endy T.P., Stephens H.A., Green S., Rothman A.L., Mathew A. (2011). Memory CD8^+^ T cells from naturally acquired primary dengue virus infection are highly cross-reactive. Immunol. Cell Biol..

[326] Zeng L., Kurane I., Okamoto Y., Ennis F.A., Brinton M.A. (1996). Identification of amino acids involved in recognition by dengue virus NS3-specific, HLA-DR15-restricted cytotoxic CD4+ T-cell clones. J. Virol..

[327] Livingston P.G., Kurane I., Dai L.C., Okamoto Y., Lai C.J., Men R., Karaki S., Takiguchi M., Ennis F.A. (1995). Dengue virus-specific, HLA-B35-restricted, human CD8+ cytotoxic T lymphocyte (CTL) clones. Recognition of NS3 amino acids 500 to 508 by CTL clones of two different serotype specificities. J. Immunol..

[328] Mongkolsapaya J., Duangchinda T., Dejnirattisai W., Vasanawathana S., Avirutnan P., Jairungsri A., Khemnu N., Tangthawornchaikul N., Chotiyarnwong P., Sae-Jang K. (2006). T cell responses in dengue hemorrhagic fever: Are cross-reactive T cells suboptimal?. J. Immunol..

[329] Kurane I., Zeng L., Brinton M.A., Ennis F.A. (1998). Definition of an epitope on NS3 recognized by human CD4+Cytotoxic T lymphocyte clones cross-reactive for dengue virus types 2, 3, and 4. Virology.

[330] Rivino L., Kumaran E.A., Thein T.-L., Too C.T., Gan V.C.H., Hanson B.J., Wilder-Smith A., Bertoletti A., Gascoigne N.R.J., Lye D.C. (2015). Virus-specific T lymphocytes home to the skin during natural dengue infection. Sci. Transl. Med..

[331] Malavige G.N., Jeewandara C., Alles K.M.L., Salimi M., Gomes L., Kamaladasa A., Jayaratne S.D., Ogg G.S. (2013). Suppression of virus specific immune responses by IL-10 in acute dengue infection. PLoS Negl. Trop. Dis..

[332] Gagnon S.J., Zeng W., Kurane I., Ennis F.A. (1996). Identification of two epitopes on the dengue 4 virus capsid protein recognized by a serotype-specific and a panel of serotype-cross-reactive human CD4+ cytotoxic T-lymphocyte clones. J. Virol..

[333] Loke H., Bethell D.B., Phuong C.X.T., Dung M., Schneider J., White N.J., Day N.P., Farrar J., Hill A.V.S. (2001). Strong HLA class I–restricted T Cell responses in dengue hemorrhagic fever: A double-edged sword?. J. Infect. Dis..

[334] Duan Z.-L., Li Q., Wang Z.-B., Xia K.-D., Guo J.-L., Liu W.-Q., Wen J.-S. (2012). HLA-A*0201-restricted CD8+ T-cell epitopes identified in dengue viruses. Virol. J..

[335] Nascimento E.J.M., Mailliard R.B., Khan A.M., Sidney J., Sette A., Guzman N., Paulaitis M., de Melo A.B., Cordeiro M.T., Gil L.V.G. (2013). Identification of conserved and HLA promiscuous DENV3 T-cell epitopes. PLoS Negl. Trop. Dis..

[336] Bashyam H.S., Green S., Rothman A.L. (2006). Dengue virus-reactive CD8+ T cells display quantitative and qualitative differences in their response to variant epitopes of heterologous viral serotypes. J. Immunol..

[337] Imrie A., Meeks J., Gurary A., Sukhbataar M., Kitsutani P., Effler P., Zhao Z. (2007). Differential functional avidity of dengue virus-specific T-cell clones for variant peptides representing heterologous and previously encountered serotypes. J. Virol..

[338] Simmons C.P., Dong T., Chau N.V., Thi N., Dung P., Nguyen T., Chau B., Thi L., Thao T., Dung N.T. (2005). Early T-cell responses to dengue virus epitopes in Vietnamese adults with secondary dengue virus infections. J. Virol..

[339] Duan Z., Guo J., Huang X., Liu H., Chen X., Jiang M., Wen J. (2015). Identification of cytotoxic T lymphocyte epitopes in dengue virus serotype 1. J. Med. Virol..

[340] Rivino L., Kumaran E.A.P., Jovanovic V., Nadua K., Teo E.W., Pang S.W., Teo G.H., Gan V.C.H., Lye D.C., Leo Y.S. (2013). Differential targeting of viral components by CD4+ versus CD8+ T lymphocytes in dengue virus infection. J. Virol..

[341] Weiskopf D., Cerpas C., Angelo M.A., Bangs D.J., Sidney J., Paul S., Peters B., Sanches F.P., Silvera C.G.T., Costa P.R. (2015). Human CD8+ T-cell responses against the 4 dengue virus serotypes are associated with distinct patterns of protein targets. J. Infect. Dis..

[342] Weiskopf D., Angelo M.A., Sidney J., Peters B., Shresta S., Sette A. (2014). Immunodominance changes as a function of the infecting dengue virus serotype and primary versus secondary infection. J. Virol..

[343] Weiskopf D., Angelo M.A., Grifoni A., O’Rourke P.H., Sidney J., Paul S., De Silva A.D., Phillips E., Mallal S., Premawansa S. (2016). HLA-DRB1 alleles are associated with different magnitudes of dengue virus–specific CD4 + T-cell responses. J. Infect. Dis..

[344] Grifoni A., Angelo M.A., Lopez B., O’Rourke P.H., Sidney J., Cerpas C., Balmaseda A., Silveira C.G.T., Maestri A., Costa P.R. (2017). Global assessment of dengue virus-specific CD4+ T cell responses in dengue-endemic areas. Front. Immunol..

[345] Kurane I., Innis B.L., Nimmannitya S., Nisalak A., Meager A., Janus J., Ennis F.A. (1991). Activation of T lymphocytes in dengue virus infections. High levels of soluble interleukin 2 receptor, soluble CD4, soluble CD8, interleukin 2, and interferon-gamma in sera of children with dengue. J. Clin. Invest..

[346] Green S., Vaughn D.W., Kalayanarooj S., Nimmannitya S., Suntayakorn S., Nisalak A., Lew R., Innis B.L., Kurane I., Rothman A.L. (1999). Early immune activation in acute dengue illness is related to development of plasma leakage and disease severity. J. Infect. Dis..

[347] Libraty D.H., Endy T.P., Houng H.H., Green S., Kalayanarooj S., Suntayakorn S., Chansiriwongs W., Vaughn D.W., Nisalak A., Ennis F.A. (2002). Differing influences of virus burden and immune activation on disease severity in secondary dengue-3 virus infections. J. Infect. Dis..

[348] Green S., Pichyangkul S., Vaughn D.W., Kalayanarooj S., Nimmannitya S., Nisalak A., Kurane I., Rothman A.L., Ennis F.A. (1999). Early CD69 expression on peripheral blood lymphocytes from children with dengue hemorrhagic fever. J. Infect. Dis..

[349] Lan N.T.P., Kikuchi M., Huong V.T.Q., Ha D.Q., Thuy T.T., Tham V.D., Tuan H.M., Tuong V.V., Nga C.T.P., Van Dat T. (2008). Protective and enhancing HLA alleles, HLA-DRB1*0901 and HLA-A*24, for severe forms of dengue virus infection, dengue hemorrhagic fever and dengue shock syndrome. PLoS Negl. Trop. Dis..

[350] Falcón-Lezama J.A., Ramos C., Zuñiga J., Juárez-Palma L., Rangel-Flores H., García-Trejo A.R., Acunha-Alonzo V., Granados J., Vargas-Alarcón G. (2009). HLA class I and II polymorphisms in Mexican Mestizo patients with dengue fever. Acta Trop..

[351] Malavige G.N., Rostron T., Rohanachandra L.T., Jayaratne S.D., Fernando N., de Silva A.D., Liyanage M., Ogg G. (2011). HLA class I and class II associations in Dengue viral infections in a Sri Lankan population. PLoS ONE.

[352] Monteiro S.P., Brasil P.E.A.A.D., Cabello G.M.K., Souza R.V.B., Brasil P., Georg I., Cabello P.H., De Castro L. (2012). HLA-A*01 allele: A risk factor for dengue haemorrhagic fever in Brazil’s population. Mem. Inst. Oswaldo Cruz.

[353] de Alencar L.X.E., de Mendonça Braga-Neto U., do Nascimento E.J.M., Cordeiro M.T., Silva A.M., de Brito C.A.A., da Silva M.d.P.C., Gil L.H.V.G., Montenegro S.M.L., Marques E.T.D.A. (2013). HLA-B*44 is associated with dengue severity caused by DENV-3 in a brazilian population. J. Trop. Med..

[354] LaFleur C., Granados J., Vargas-Alarcon G., Ruíz-Morales J., Villarreal-Garza C., Higuera L., Hernández-Pacheco G., Cutiño-Moguel T., Rangel H., Figueroa R. (2002). HLA-DR antigen frequencies in Mexican patients with dengue virus infection: HLA-DR4 as a possible genetic resistance factor for dengue hemorrhagic fever. Hum. Immunol..

[355] Gagnon S.J., Ennis F.A., Rothman A.L. (1999). Bystander target cell lysis and cytokine production by dengue virus-specific human CD4^+^ cytotoxic T-lymphocyte clones. J. Virol..

[356] Mangada M.M., Endy T.P., Nisalak A., Chunsuttiwat S., Vaughn D.W., Libraty D.H., Green S., Ennis F.A., Rothman A.L. (2002). Dengue-Specific T Cell responses in peripheral blood mononuclear cells obtained prior to secondary dengue virus infections in Thai schoolchildren. J. Infect. Dis..

[357] Mangada M.M., Rothman A.L. (2005). Altered cytokine responses of dengue-specific CD4+ T cells to heterologous serotypes. J. Immunol..

[358] An J., Zhou D.-S., Zhang J.-L., Morida H., Wang J.-L., Yasui K. (2004). Dengue-specific CD8+ T cells have both protective and pathogenic roles in dengue virus infection. Immunol. Lett..

[359] Talarico L.B., Batalle J.P., Byrne A.B., Brahamian J.M., Ferretti A., García A.G., Mauri A., Simonetto C., Hijano D.R., Lawrence A. (2017). The role of heterotypic DENV-specific CD8+ T lymphocytes in an immunocompetent mouse model of secondary dengue virus infection. EBioMedicine.

[360] Hsieh M.-F., Lai S.-L., Chen J.-P., Sung J.-M., Lin Y.-L., Wu-Hsieh B.A., Gerard C., Luster A., Liao F. (2006). Both CXCR3 and CXCL10/IFN-inducible protein 10 are required for resistance to primary infection by dengue virus. J. Immunol..

[361] Amorim J.H., dos Santos Alves R.P., Bizerra R., Pereira S.A., Pereira L.R., Fabris D.L.N., Santos R.A., Romano C.M., de Souza Ferreira L.C. (2016). Antibodies are not required to a protective immune response against dengue virus elicited in a mouse encephalitis model. Virology.

[362] Zellweger R.M., Eddy W.E., Tang W.W., Miller R., Shresta S. (2014). CD8+ T cells prevent antigen-induced antibody-dependent enhancement of dengue disease in mice. J. Immunol..

[363] Zompi S., Santich B.H., Beatty P.R., Harris E. (2012). Protection from secondary dengue virus infection in a mouse model reveals the role of serotype cross-reactive B and T cells. J. Immunol..

[364] Zellweger R.M., Tang W.W., Eddy W.E., King K., Sanchez M.C., Shresta S. (2015). CD8+ T cells can mediate short-term protection against heterotypic dengue virus reinfection in mice. J. Virol..

[365] Ng K.-H., Zhang S.L., Tan H.C., Kwek S.S., Sessions O.M., Chan C.-Y., Liu I.D., Lee C.K., Tambyah P.A., Ooi E.E. (2019). Persistent dengue infection in an immunosuppressed patient reveals the roles of humoral and cellular immune responses in virus clearance. Cell Host Microbe.

[366] Simon-Lorière E., Duong V., Tawfik A., Ung S., Ly S., Casadémont I., Prot M., Courtejoie N., Bleakley K., Buchy P. (2017). Increased adaptive immune responses and proper feedback regulation protect against clinical dengue. Sci. Transl. Med..

[367] de Matos A.M., Carvalho K.I., Rosa D.S., Villas-Boas L.S., da Silva W.C., de Lima Rodrigues C.L., Oliveira O.M.N.P.F., Levi J.E., Araújo E.S.A., Pannuti C.S. (2015). CD8+ T lymphocyte expansion, proliferation and activation in dengue fever. PLoS Negl. Trop. Dis..

[368] Wijeratne D.T., Fernando S., Gomes L., Jeewandara C., Ginneliya A., Samarasekara S., Wijewickrama A., Hardman C.S., Ogg G.S., Malavige G.N. (2018). Quantification of dengue virus specific T cell responses and correlation with viral load and clinical disease severity in acute dengue infection. PLoS Negl. Trop. Dis..

[369] Hatch S., Endy T.P., Thomas S., Mathew A., Potts J., Pazoles P., Libraty D.H., Gibbons R., Rothman A.L. (2011). Intracellular cytokine production by dengue virus–specific T cells correlates with subclinical secondary infection. J. Infect. Dis..

[370] Jeewandara C., Adikari T.N., Gomes L., Fernando S., Fernando R.H., Perera M.K.T., Ariyaratne D., Kamaladasa A., Salimi M., Prathapan S. (2015). Functionality of dengue virus specific memory T cell responses in individuals who were hospitalized or who had mild or subclinical dengue infection. PLoS Negl. Trop. Dis..

[371] Wijeratne D.T., Fernando S., Gomes L., Jeewandara C., Jayarathna G., Perera Y., Wickramanayake S., Wijewickrama A., Ogg G.S., Malavige G.N. (2019). Association of dengue virus-specific polyfunctional T-cell responses with clinical disease severity in acute dengue infection. Immun. Inflamm. Dis..

[372] Dung N.T.P., Le Duyen H.T., Van Thuy N.T., Van Ngoc T., Chau N.V.V., Hien T.T., Rowland-Jones S.L., Dong T., Farrar J., Wills B. (2010). Timing of CD8+ T cell responses in relation to commencement of capillary leakage in children with dengue. J. Immunol..

[373] de Alwis R., Bangs D.J., Angelo M.A., Cerpas C., Fernando A., Sidney J., Peters B., Gresh L., Balmaseda A., de Silva A.D. (2016). Immunodominant dengue virus-specific CD8+ T cell responses are associated with a memory PD-1+ phenotype. J. Virol..

[374] Weiskopf D., Bangs D.J., Sidney J., Kolla R.V., De Silva A.D., de Silva A.M., Crotty S., Peters B., Sette A. (2015). Dengue virus infection elicits highly polarized CX3CR1+ cytotoxic CD4+ T cells associated with protective immunity. Proc. Natl. Acad. Sci. USA.

[375] Tian Y., Babor M., Lane J., Schulten V., Patil V.S., Seumois G., Rosales S.L., Fu Z., Picarda G., Burel J. (2017). Unique phenotypes and clonal expansions of human CD4 effector memory T cells re-expressing CD45RA. Nat. Commun..

[376] Patil V.S., Madrigal A., Schmiedel B.J., Clarke J., O’Rourke P., de Silva A.D., Harris E., Peters B., Seumois G., Weiskopf D. (2018). Precursors of human CD4+ cytotoxic T lymphocytes identified by single-cell transcriptome analysis. Sci. Immunol..

[377] Gebhardt T., Wakim L.M., Eidsmo L., Reading P.C., Heath W.R., Carbone F.R. (2009). Memory T cells in nonlymphoid tissue that provide enhanced local immunity during infection with herpes simplex virus. Nat. Immunol..

[378] Guy B., Nougarede N., Begue S., Sanchez V., Souag N., Carre M., Chambonneau L., Morrisson D.N., Shaw D., Qiao M. (2008). Cell-mediated immunity induced by chimeric tetravalent dengue vaccine in naive or flavivirus-primed subjects. Vaccine.

[379] Harenberg A., Begue S., Mamessier A., Gimenez-Fourage S., Ching Seah C., Wei Liang A., Li Ng J., Yun Toh X., Archuleta S., Wilder-Smith A. (2013). Persistence of Th1/Tc1 responses one year after tetravalent dengue vaccination in adults and adolescents in Singapore. Hum. Vaccin. Immunother..

[380] Grifoni A., Voic H., Dhanda S.K., Kidd C.K., Brien J.D., Buus S., Stryhn A., Durbin A.P., Whitehead S., Diehl S.A. (2020). T cell responses induced by attenuated flavivirus vaccination are specific and show limited cross-reactivity with other flavivirus species. J. Virol..

[381] Waickman A.T., Friberg H., Gargulak M., Kong A., Polhemus M., Endy T., Thomas S.J., Jarman R.G., Currier J.R. (2019). Assessing the diversity and stability of cellular immunity generated in response to the candidate live-attenuated dengue virus vaccine TAK-003. Front. Immunol..

[382] Chu H., George S.L., Stinchcomb D.T., Osorio J.E., Partidos C.D. (2015). CD8+ T-cell responses in flavivirus-naive individuals following immunization with a live-attenuated tetravalent dengue vaccine candidate. J. Infect. Dis..

[383] Weiskopf D., Angelo M.A., Bangs D.J., Sidney J., Paul S., Peters B., de Silva A.D., Lindow J.C., Diehl S.A., Whitehead S. (2015). The human CD8+ T cell responses induced by a live attenuated tetravalent dengue vaccine are directed against highly conserved epitopes. J. Virol..

[384] Angelo M.A., Grifoni A., O’Rourke P.H., Sidney J., Paul S., Peters B., de Silva A.D., Phillips E., Mallal S., Diehl S.A. (2017). Human CD4+ T cell responses to an attenuated tetravalent dengue vaccine parallel those induced by natural infection in magnitude, HLA restriction, and antigen specificity. J. Virol..

[385] Graham N., Eisenhauer P., Diehl S.A., Pierce K.K., Whitehead S.S., Durbin A.P., Kirkpatrick B.D., Sette A., Weiskopf D., Boyson J.E. (2020). Rapid induction and maintenance of virus-specific CD8^+^ T_EMRA_ and CD4^+^ T_EM_ cells following protective vaccination against dengue virus challenge in humans. Front. Immunol..

[386] Yauch L.E., Prestwood T.R., May M.M., Morar M.M., Zellweger R.M., Peters B., Sette A., Shresta S. (2010). CD4 + T Cells Are not required for the induction of dengue virus-specific CD8 + T cell or antibody responses but contribute to protection after vaccination. J. Immunol..

[387] Zellweger R.M., Miller R., Eddy W.E., White L.J., Johnston R.E., Shresta S. (2013). Role of humoral versus cellular responses induced by a protective dengue vaccine candidate. PLoS Pathog..

[388] Gil L., Izquierdo A., Lazo L., Valdés I., Ambala P., Ochola L., Marcos E., Suzarte E., Kariuki T., Guzmán G. (2014). Capsid protein: Evidences about the partial protective role of neutralizing antibody-independent immunity against dengue in monkeys. Virology.

[389] Costa S.M., Yorio A.P., Gonçalves A.J.S., Vidale M.M., Costa E.C.B., Mohana-Borges R., Motta M.A., Freire M.S., Alves A.M.B. (2011). Induction of a protective response in mice by the dengue virus NS3 protein using DNA vaccines. PLoS ONE.

[390] Simmons M., Sun P., Putnak R. (2016). Recombinant dengue 2 virus NS3 helicase protein enhances antibody and T-cell response of purified inactivated vaccine. PLoS ONE.

[391] Kao Y.-S., Yu C.-Y., Huang H.-J., Tien S.-M., Wang W.-Y., Yang M., Anderson R., Yeh T.-M., Lin Y.-S., Wan S.-W. (2019). Combination of modified NS1 and NS3 as a novel vaccine strategy against dengue virus infection. J. Immunol..

[392] Gonçalves A.J.S., Oliveira E.R.A., Costa S.M., Paes M.V., Silva J.F.A., Azevedo A.S., Mantuano-Barradas M., Nogueira A.C.M.A., Almeida C.J., Alves A.M.B. (2015). Cooperation between CD4+ T cells and humoral immunity is critical for protection against dengue using a DNA vaccine based on the NS1 antigen. PLoS Negl. Trop. Dis..

[393] Pinto P.B.A., Assis M.L., Vallochi A.L., Pacheco A.R., Lima L.M., Quaresma K.R.L., Pereira B.A.S., Costa S.M., Alves A.M.B. (2019). T cell responses induced by DNA vaccines based on the DENV2 E and NS1 proteins in mice: Importance in protection and immunodominant epitope identification. Front. Immunol..

[394] dos Santos Alves R.P., Pereira L.R., Fabris D.L.N., Salvador F.S., Santos R.A., de Andrade Zanotto P.M., Romano C.M., Amorim J.H., de Souza Ferreira L.C. (2016). Production of a recombinant dengue virus 2 NS5 protein and potential use as a vaccine antigen. Clin. Vaccine Immunol..

[395] Roth C., Cantaert T., Colas C., Prot M., Casadémont I., Levillayer L., Thalmensi J., Langlade-Demoyen P., Gerke C., Bahl K. (2019). A modified mRNA vaccine targeting immunodominant NS Epitopes protects against dengue virus infection in HLA Class I transgenic mice. Front. Immunol..

[396] Ngono A.E., Chen H.-W., Tang W.W., Joo Y., King K., Weiskopf D., Sidney J., Sette A., Shresta S. (2016). Protective role of cross-reactive CD8 T cells against dengue virus infection. EBioMedicine.

[397] Wang P.-G., Kudelko M., Lo J., Siu L.Y.L., Kwok K.T.H., Sachse M., Nicholls J.M., Bruzzone R., Altmeyer R.M., Nal B. (2009). Efficient assembly and secretion of recombinant subviral particles of the four dengue serotypes using native prM and E proteins. PLoS ONE.

